# Transmitted/founder (T/F) HIV-1 derived from sexual contact exhibits greater transmission fitness in human cervical tissue than T/F HIV-1 from blood-to-blood contact: Unique glycan profiles on T/F envelopes associated with transmission phenotypes

**DOI:** 10.1371/journal.ppat.1013177

**Published:** 2025-05-23

**Authors:** Yiying Zhang, Katja Klein, Annette Ratcliff, Sashini Loku Galappaththi, Nicholas Hathaway, Nicholas Twells, Mukti Patel, Stephen Temesy, Jeffrey A. Bailey, Lara K. Mahal, Carole Creuzenet, Eric J. Arts

**Affiliations:** 1 Department of Microbiology and Immunology, University of Western Ontario, London, Canada; 2 Bristol Veterinary School, University of Bristol, Bristol, United Kingdom; 3 Department of Molecular Biology and Microbiology and Division of Infectious Diseases, Case Western Reserve University, Cleveland, United States of America; 4 Department of Pathology and Laboratory Medicine, Brown University, Providence, United States of America; 5 Department of Chemistry, University of Alberta, Edmonton, Canada; Vaccine Research Center, UNITED STATES OF AMERICA

## Abstract

Human immunodeficiency virus 1 (HIV-1) risk groups include, but are not limited to, heterosexual individuals (HET), men-who-have-sex-with-men (MSM), and people who inject drugs (PWID). Although genetically diverse HIV-1 populations are transferred from donor to recipient, systemic infection is often established by a single clone, the transmitted/founder (T/F) virus. This phenomenon is especially prevalent in sexual transmission, but less stringent in blood-to-blood contact transmission. Specific traits that permit successful transmission have not been well characterized. Thus, HIV-1 containing the chimeric T/F envelope (Env) from different transmission routes was assessed for ex vivo transmission fitness by performing mixed competition assays (also referred to as mixed competitions) on human cervical tissues. We found that chimeric T/F viruses isolated from the PWID exhibit limited replication capacity in cervical tissues when compared to those from MSM and HET, diminishing their chances of transmission to T helper type 1 (Th1) and Th17 cells. This reduced transmission fitness of T/F HIV-1 from PWID was not observed when infecting Th1 and Th17 cells directly, bypassing cervical tissues. Phenotypic assays showed that the chimeric T/F viruses from PWID differed from other groups by having an enhanced ability to utilize diverse CCR5 conformations, while Env expression level, CD4/CCR5 utilization, and entry speed did not differ. Different glycosylation profiles were detected on T/F compared to chronic Env with increased complex, fucosylated N- and O-glycans found more frequently on the T/F Env. Furthermore, the increased presence of these fucosylated glycans correlated with replication fitness in cervical tissues. In contrast, bisecting branched N-glycan found more frequently on chronic Env was associated with decreased entry efficiency and more stringent usage of CCR5. These findings suggest that glycosylation patterns/levels and/or Env structure greatly impact the differences in transmission fitness of T/F HIV-1.

## Author summary

Our investigation focused on the human immunodeficiency virus 1 (HIV-1) strains that establish infections, known as transmitted/founder (T/F) viruses. These viruses differ in their ability to infect new hosts depending on the transmission route, such as through unprotected sex or drug injection. Using the explant cervical tissue for a model of penile-to-vaginal transmission, multi-T/F-virus competitions revealed that the sexually transmitted HIV-1 had higher transmission fitness than HIV-1 derived from blood-to-blood contact in people who inject drugs (PWID). T/F viruses from PWID also displayed more efficient utilization of maraviroc-bound CCR5. Additionally, N-glycan levels on the viral envelope (Env) were found to correlate with multiple phenotypic traits, including the ability to replicate on human cervical tissues. These findings suggest that the glycosylation patterns/levels on viral Env and Env structures may affect the virus’ ability to establish infection. This study highlights the differences of T/F viruses from various transmission routes and suggests that glycan content may play a crucial role in influencing HIV-1 transmission fitness.

## Introduction

Human immunodeficiency virus-1 (HIV-1) is transmitted by contact with infected fluids, including genital secretions (during sex) or blood. HIV-1 risk groups include, but are not limited to, heterosexual individuals (HET), men-who-have-sex-with-men (MSM), and people who inject drugs (PWID). In the process of transmission, only a single HIV-1 clone, or in rare circumstances, a few clones establish infection within the recipients despite the fact that almost all infected donors harbour a diverse HIV-1 population (quasispecies or swarm) within the transmitted fluid and regardless of the mode of transmission [1,2]. It is unclear whether this HIV-1 transmission bottleneck leading to primary infection by transmitted/founder (T/F) virus(es) [3,4] from the donor is a stochastic event, involves selection for specific viral traits, or a combination of both [5–7]. The chance of single-clone transmission is approximately 80% in HET, 75% in MSM, and 40–80% in PWID [1,2], suggesting that the bottleneck is more stringent in transmission across mucosal tissue.

Multiple bottlenecks likely exist, as compartmentalized HIV-1 must infect sequential tissues while moving from donor to recipient, especially during heterosexual and homosexual modes of transmission. During chronic disease, infection of tissues (e.g., brain/CNS, lung, genital tissues) often leads to compartmentalization of HIV-1, characterized by divergent HIV-1 sub-populations with higher or lower genetic diversity than observed in the blood and primary/secondary lymphoid tissues [8–13]. In the case of a transmission stemming from donor semen deposition into the recipient vaginal tract, the HIV-1 inoculum is a combination of the diverse compartmentalized virus delivered from seminal plasma and infected seminal T cells [14–16]. Newly infected women/females are typically infected by multiple HIV-1 clones in their genital tract and yet have only one HIV-1 clone establishing disseminated infection in their blood [17]. Thus, in the majority of cases, multiple genetic bottlenecks related to various host factors are likely involved in “filtering” the inoculum HIV-1 population to arrive at a single HIV-1 clone establishing viremia from male to female penile-vaginal intercourse. For example, inflammation (including that caused by sexually transmitted diseases) [18,19] in the genital tract can recruit HIV-1 susceptible cells, such as CD4 + T cells and myeloid cells (especially dendritic cells [DCs]), and therefore cause a burst of viral replication, which may diversify the genetic population in the genital tract during transmission (in the donor or recipient) [14,20,21]. Many of these processes including HIV-1 trans-infection of T cells via DCs have been visualized in macaque and human vaginal/cervical tissue [22–25]

In blood-to-blood transmission, as is the case with PWID, physical barriers are minimal or absent but an HIV-1 bottleneck remains, albeit reduced when compared to that observed with primary HET and MSM infections [1,2]. It has been suggested that the presence of a genetic bottleneck during PWID transmission may relate to the transfer of small amounts of “contaminating” blood (from re-used needles) resulting in fewer HIV-1 variants being introduced, dispersed and diluted into the donor blood [2]. Nonetheless, systemic HIV-1 infection may still be mediated by DCs capturing inoculating HIV-1 for transport to the draining lymph nodes, where HIV-1 antigen presentation and infection of resident, activated CD4 + T cells occurs almost simultaneously [26,27]. Thus, the transmission bottleneck may also be related to the binding affinity of HIV-1 envelope (Env) to CD4 and/or CCR5 [28,29]. Many T/F HIV-1 clones have higher avidity/affinity for CD4 and may require strict CCR5 conformation for entry, as compared to HIV-1 derived from chronic infection [30].

In addition to physical barriers, numerous host and tissue specific factors reduce the sexual transmission of HIV. There are innate immune factors such as interferon-𝛼 that induce an antiviral state to constrain HIV-1 replication [31]. The mucus on the surface of vaginal/cervical and rectal mucosal epithelia is abundant with the microbiome, mucins, various antiviral molecules, and C-type lectins, all involved in keeping pathogens out and as such, reducing viral transmissibility [24,32–37]. In addition, small intercellular spaces and structures, such as desmosome and lipoidal material, make it hard for virions to get through the intact multi-layer mucosal epithelium [38]. Once HIV-1 has breached the mucosal epithelium, possibly aided by microabrasions, Langerhans cells (LCs), tissue-resident macrophages, and DCs, such as cDC2 whose findings were largely in line with, can capture HIV-1 mainly via Langerin, Siglec-1, and C-type lectin receptors (such as DC-SIGN on macrophages [39–41]), facilitating trans-infection of tissue resident CD4 + T cells [39,42–52]. Infected CD4 + T cells and DCs can migrate into mucosal lymphoid follicles or draining lymph nodes, initiating the systemic dissemination of HIV-1 within the human host [53,54]. The virus can also directly infect tissue resident CD4 + T cells (mainly memory T cells) in the mucosa, but this is more likely to occur in the sub-mucosal layers, e.g., lamina propria [2,17,54–56]. DC-mediated trans-infection of these mucosal or sub-mucosal T cells is more likely [25,38–41,54,56].

In a previous study using mixed HIV-1 infections, T/F HIV-1 were transmitted through cervical and penile mucosal tissue significantly better than HIV-1 isolates derived from chronic infections [24]. In this study, we assessed the transmission fitness and phenotypic properties of T/F HIV-1 derived from newly infected individuals exposed to HIV-1 by different modes/routes of transmission. This study involved a series of assays to probe the efficiency of HIV-1 entry and infection related to the different modes of transmission. Through mixed competition assays (also referred to as mixed competitions) in human cervical tissues with T helper type 1 (Th1) and Th17 cell lines, we evaluated the ex vivo transmission fitness of the chimeric HIV-1 strains with Env derived from HET, MSM, and PWID transmission. Env expression, CCR5 conformation utilization, cellular entry speed, CD4/CCR5 utilization efficiency, and glycosylation (via lectin binding array) were also measured in the same viruses. The following describes clear phenotypic differences and glycosylation profiles of HIV-1 based on transmission routes and/or stage of disease. Variations in HIV-1 Env glycosylation may be a key factor influencing the type of HIV-1 selected during different modes of transmission.

## Results

### Chimeric HIV-1 construction and Env expression evaluation

**Fig 1:**
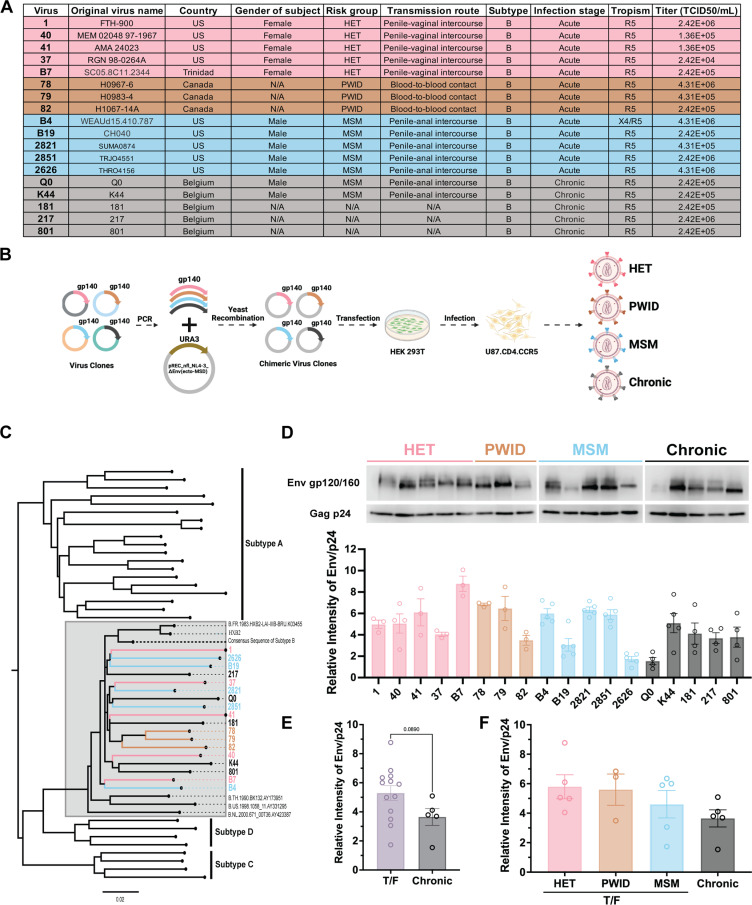
Construction of Chimeric HIV-1. **(A)** Characteristics of Transmitted/Founder (T/F) and chronic HIV-1 viruses. N/A, not available. The table is colour-coded by risk group (pink: HET, brown: PWID, blue: MSM, grey: chronic viruses). **(B)** Schematic representation of chimeric virus construction (created with BioRender.com). The gp140 of *env* was cloned into pREC_nfl_NL4-3_Δenv (ecto-MSD) using yeast-based recombination followed by transfection into HEK 293T cells to generate functional chimeric viruses expressing different envelope (Env) gp140 proteins. Each chimeric virus was propagated and titrated on U87.CD4.CCR5 using 50% tissue culture infective dose (TCID_50_) assay. **(C)** A phylogenetic tree generated from DNA sequences of gp140 from chimeric viruses and reference sequences from different HIV subtypes using the neighbour-joining method. **(D)** Evaluation of Env expression levels by Western blotting. SDS-PAGE and Western blot probing p24 (D45F) and gp120 (B13) were performed on chimeric viruses and the signal intensity was quantified by ImageJ. Based on the uniformity of the p24 expression, the Env/p24 ratio was then calculated to compare the Env expression level. **(E)** Relative Env expression level comparison between T/F and chronic viruses. The student t-test was performed. **(F)** Relative Env expression levels of viruses from different risk groups. One-way ANOVA followed by Tukey’s multiple comparisons was performed.

Previous studies suggest that HIV-1 derived from different routes of primary infection may exhibit different phenotypic properties, despite the absence of defined genotypic sequences [24,29,57,58]. For example, HIV-1 isolates derived from acute/early infection showed greater variations in replicative fitness and CD4/CCR5 affinity than HIV-1 obtained at different times during chronic infection (in the absence of treatment) [24,57]. However, studies on transmission fitness based on different modes of primary infection are limited by the availability of samples from the primary infections with the same HIV-1 subtype and uncertainties about the modes of transmission. Thus, for this study, we constructed Env chimeric T/F subtype B HIV-1 from risk populations (HET, MSM, and PWID) exposed via different transmission routes (sexual or blood contact transmission). These samples were obtained as part of primary infection cohorts in Seattle, Los Angeles, San Francisco, New York, and Boston [59–67] (Fig 1A). Previous studies revealed that the dominant traits of replicative and transmission fitness of HIV-1 are mapped to the *env* gene [3,29,68,69]. As illustrated in Fig 1A and 1B, the ectodomains of Env gp120 and gp41 (gp140) of these acute HIV-1 and HIV-1 from chronic infections were cloned with the Env gp41 transmembrane and intracellular domains of NL4–3 within an NL4–3 backbone (pREC_nfl_NL4–3_ΔEnv_ecto-MSD) using the yeast recombination/gap repair methods and complement rescue system [70]. For this study, we employed 5 T/F Env chimeric viruses from HET (infected through penile-vaginal transmission), 3 T/F viruses from PWID (infected through blood-blood contact transmission), 5 T/F viruses from MSM (infected through penile-anal transmission) and 5 Env chimeric viruses from chronic subtype B infections.

Despite chimeric viruses representing multiple individuals in each transmission group, the specific coding sequence of different T/F Env did not appear to have any specific sequences associated with different modes of transmission (Fig 1C). Levels of Env within each chimeric HIV-1 isolate, relative to the NL4–3 capsid/p24 proteins, were measured by Western blotting with antibodies targeting invariant epitopes in gp120 and p24 (Fig 1D, full blot provided in S1 Fig) quantitation of band intensity of gp120 and p24 is shown in Fig 1E and 1F. There was no significant difference in the level of HIV-1 Env on any of the chimeric viruses. Even though the anti-Env B13 antibody recognizes a relatively conserved linear epitope in gp120, there is still more or less recognition of these distinct HIV-1 Env glycoproteins by this antibody, even if present at the same levels on virus particles. The B13 antibody still has better cross-recognition of gp120 that other anti-gp120 antibodies such as 2G12, B12, and VRC03. The NL4–3 HIV-1 Gag is present in all of these chimeric HIV-1 which provides a reasonable reference. Please note that in previous studies, variations in HIV-1 host cell entry efficiency were typically related to differences in Env structure/sequence rather than variations in Env levels per particle [24,71,72].

### Multi-virus competitions on human cervical tissue explants

**Fig 2:**
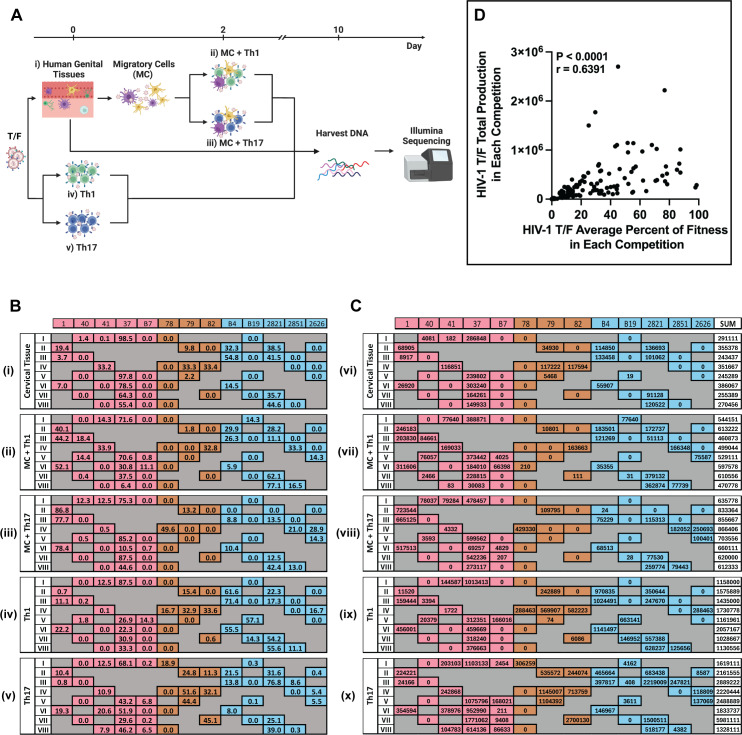
HIV-1 multi-virus competitions in human genital tissues. **(A)** Schematic representation of multi-virus competitions (created with BioRender.com). A mix of 6 T/F viruses from different risk groups, each at 3750 IUs, was added to human explant genital tissue pieces for 6 hours. The tissue pieces were then washed with PBS and cultured in fresh medium for 10 days. Migratory cells (MCs) leaving the tissue were collected 48 hours post-infection and co-cultured with human Th1 or Th17 cell lines for 10 days. Concurrently, the mixture of T/F viruses was also added to Th1 or Th17 cell lines directly to compare the effect of tissue on virus competition. DNA was extracted from tissue, MC and Th1 co-cultures (MC + Th1), MC and Th1 co-cultures (MC + Th17), Th1, and Th17. The C2-V3-C3 region of *env* was PCR amplified for next-generation sequencing using Illumina. **(B)** The percent of replication fitness for the individual virus in each competition on cervical tissue (i), MC + Th1 (ii), MC + Th17 (iii), Th1 (iv) and Th17 (v). Eight competitions were designed, each involving 6 different chimeric T/F viruses, allowing each virus to compete with every other virus at least once. The viruses that participated in each competition were coloured by transmission group. Each competition was performed in tissue from 3 donors (normally in triplicate) or on Th1/Th17 cells 3 times in triplicate. The results were generated from SeekDeep analysis on Illumina sequencing results. **(C)** Total production of individual virus in each competition on cervical tissue (vi), MC + Th1 (vii), MC + Th17 (viii), Th1 (ix) and Th17 (x). Total production was calculated by multiplying the relative copy number per mitochondria DNA (from S12 Fig qPCR results) by the average percentage fitness in competitions. **(D)** Pearson correlation analysis between the average percent of fitness (data from B) and the total production (data from C) of each HIV-1 T/F in mixed infections.

Our primary comparator group for transmission fitness was the HET chimeric T/F HIV-1 derived from female recipient blood following penile-vaginal transmission. As such, our primary tissue culture model system involved human cervical tissue explants as the receptacle for infection followed by subsequent collection of the tissue migratory cells (MCs) for co-incubation with human Th1, and Th17 cell lines [7,24]. Healthy cervical tissue was obtained from hysterectomies, frozen, thawed and cut into 5 mm^3^ sections, which included the mucosa, a single layer of columnar epithelial cells, and the lamina propria as described in our previous confocal microscopy analyses [24]. Our previous studies and those from other groups have identified multiple immune cell populations in these explant cervical (and penile) tissues including resident memory CD4 + T cells along with mDC populations derived that are 28% for CD1c positive, 12% CD207 (langerin) positive, and 7.69% CD209 (DC-SIGN) positive (Fig 2), which is in accordance with previous findings [24,73–75] Th1 and Th17 cells are abundant in both the blood and mucosa and are highly permissive to HIV-1 infection [76–79]. Competitive infections with multiple HIV-1 isolates provide the ability to determine the relative production of each virus against the others. In mono-infections, virus replication is absolute and not internally controlled, resulting in higher variability per replicate [80,81]. In addition, viral mixtures recapitulate competitions between several HIV-1 clones within the quasispecies in the inoculum during transmission [82,83].

Eight mixtures (competition I to VIII) containing 6 different T/F HIV-1 were added to the human endocervical tissues, human Th1, or Th17 clonal cell lines in a round-robin setup such that each virus was competed at least once with each of the remaining 12 T/F viruses. The clonal Th1 and Th17 cell lines provide continuity between all mixed virus infections considering that autologous peripheral blood mononuclear cells were not available for the majority of individuals who consented to provide discarded cervical tissue. Competitions were performed on each of 3 separate donor tissues, with each competition conducted in triplicate per donor tissue, resulting in a total of 72 mixed-virus competitions (8 competitions x 3 donors x 3 replicates). MCs, released from the exposed tissue at 48 hours, mainly DCs and LCs were collected in the supernatant and co-cultured with Th1 and with Th17 cell lines (schematically represented in Fig 2A). DNA was extracted from tissue explants, MC + Th1 and MC + Th17 co-cultures, and from Th1 and Th17 infections after culturing for 10 days. Virus production and transmission were assessed by amplifying and sequencing the C2-V3-C3 region of *env* using Illumina MiSeq and analyzed by SeekDeep. Previous studies had performed similar round-robin, multi-virus competitions using HIV-1 derived from chronic infections versus acute/early infections, with most T/F from MSM transmissions [24]. These studies revealed that the MSM T/F HIV-1 had higher ex vivo transmission fitness in genital tissues compared with chronic HIV-1 (using the same methods described above).

**Fig 3:**
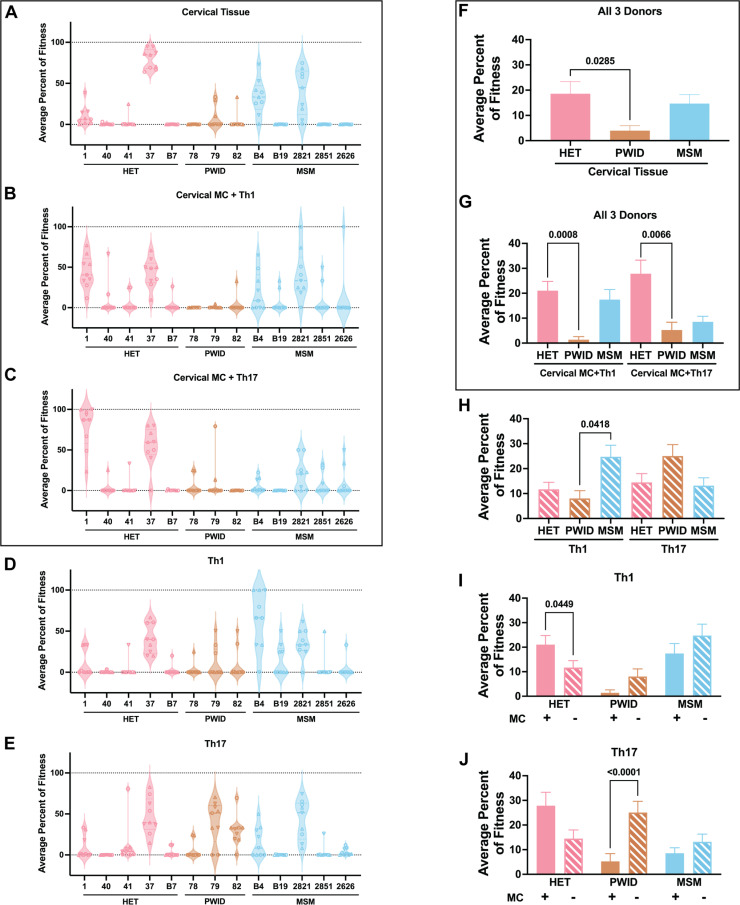
Average percent of fitness of chimeric T/F HIV-1 from different risk groups. **(A-E)** The average percent of fitness of each T/F chimera in cervical tissue, migratory cells and Th1 co-culture (MC + Th1), migratory cells and Th17 co-culture (MC + Th17), Th1 and Th17. The average percent of fitness was calculated based on the virus content in all replicates of all donors. Different shapes represent data different donors. **(F-H)** The average percent of fitness in cervical tissue, MC + Th1, MC + Th17, Th1 and Th17 grouped by different risk groups. The Kruskal-Wallis test followed by Dunn’s multiple comparisons test was performed. **(I-J)** Comparison of average percent of fitness of T/F viruses on Th1 and Th17 with and without MCs. Mann-Whitney tests were performed.

SeekDeep clusters and aligns input sequences to reference sequences [84], calculating the read counts and percentages of each T/F virus in each mixed infection (Figs 2B and S4-S10). Virus HET-37 and MSM-2821 showed the greatest transmission fitness, determined as the viruses entering the cervical tissue, being captured by MCs, and then transferred to infect either Th1 or Th17 cells. HET-37 and MSM-2821 chimeras also had the highest replicative fitness in the cervical tissue. Direct exposure of these same virus mixtures to only the Th1 or Th17 cells also showed that HET-37 and MSM-2821 outcompeted the other T/F HIV-1 chimeras, MSM-B4 and PWID-79 being the exception. In contrast, most T/F chimeras showed disparate replicative capacity in these mixed infections dependent on the exposed tissue and cell types. For example, MSM-B4 had high transmission fitness through the tissue-MC-Th1 route but less so through the tissue-MC-Th17 route (Fig 3A-C). It is notable that virus HET-1 replicated relatively poorly in cervical tissues but was still carried efficiently by the tissue MCs to infect and replicate in both Th1 and Th17 (Fig 3A-C). However, when HET-1 was added directly to Th1 and Th17 cells, replication efficiency was in the median range of the other T/F HIV-1. It is possible that the HET-1 virus (as well as other T/F HIV-1) may be dependent on DC/LC mediated trans-infection of T cells to establish primary infection without establishing primary infection in the mucosal tissue [43–45,48]. In contrast, most of the HET and MSM T/F HIV-1 appear to infect resident cells in the cervical mucosa while also being carried by cells (likely DC/LC) that migrate out of the tissue for the transfer of HIV-1 to CD4 + T cells in the submucosal layers. In vivo, this process of mucosal infection followed by transmigration for submucosal CD4 + T cell infections could lead to systemic infection [55].

The PWID-78, -79, and -82 viruses were rarely detected in the mixed infections of cervical tissue or transferred Th1 and Th17 cells from the cervical tissue by cervical-derived MCs. Nonetheless, all T/F HET and MSM chimeras could replicate and transmit through cervical tissue to T cells in the majority of the mixed virus competitions. There were exceptions. In competition IV, PWID-79 and PWID-82 reached ~33% of the total amount of virus replicating in cervical tissue, which also contained HET-41, PWID-78, MSM-2851 and MSM-2626 (Fig 2B). Despite relative outgrowth differences between chimeric T/F HIV-1 in these competitions, qPCR of the *gag* region (identical in the NL4–3 backbone) revealed that the relative virus copy number (combination of all T/F chimeras in a competition) was similar in all I through VIII competitions (within as specific tissue or cells) (S12 Fig). However, the total virus copy number from Th1 and Th17 competitions was approximately 10-fold greater than the total virus copy number that had to pass through or replicate in the cervical tissue (i.e., cervical tissue, MC + Th1, or MC + Th17 (S12 Fig)).

When the average fitness of chimeric T/F HIV-1 was grouped by risk groups, the PWID chimeras had significantly lower transmission fitness using cervical tissue as compared to the HET chimeras (P = 0.0285, Dunn’s). We define surrogate of transmission fitness as the ability to infect the first contact tissue and then to transfer and propagate in a specific CD4 + T cell population. In the case of HIV-1 transmission fitness in the female genital tract, we employed female endocervical tissue exposed to and infected by the HIV-1 variants and then examined transfer to and replication in Th1/Th17. The PWID chimeras had lower fitness than the MSM chimeras in cervical tissue based on a paired two-tailed Mann-Whitney (P = 0.0379) but did not reach statistical significance when considering all potential pairs using Dunn’s multiple comparison test (Fig 3F and 3G). Low transmission fitness with the PWID chimeras were observed in the mixed virus competition performed in each of the 3 donors (S11 Fig). In contrast, PWID chimeras had higher replicative fitness in cultures of Th1 or Th17 alone compared to HET chimeras (Fig 3H-J). This observation is consistent with the notion that PWID T/F HIV-1 may be more dependent on efficient replication in CD4 + T cells to establish primary infection, while having properties to establish infection through the mucosal route may not be necessary.

Because there were only 3 T/F HIV-1 in the PWID group, there were concerns about probability bias. By multiplying the total virus production (based on real-time PCR of viral DNA and the fraction of each virus in the mixed competition, we obtained the absolute production of each T/F HIV in that mixed competition (Fig 2C). For the mixed virus infections in tissue or in tissue MC with Th1 and Th17, and Th1 or Th17 alone, the absolute production of each virus in each mixed infection correlated directly with the relative fitness of each virus in the mixed infections (p < 0.0001), with no inherent bias for or against PWID T/F HIV-1 relative to the other T/F HIV-1 (Fig 2D). As previously described, starting in 2003 [85,86], the mixed infections described herein are not competition for susceptible cells and resources. Due to the low MOI (< 0.01) of the virus to susceptible cells in this tissue coupled with the extracellular matrix that limits diffusion, there is no chance of capacity infection during the ten days of incubation, which reflects the largest inoculum of infectious HIV-1 in donor semen deposited into the vaginal vault during heterosexual contact. In the Th1 and Th17 mixed infections where susceptible cells could be saturated by 10 days, there is a potential bias of fewer PWID T/F HIV-1 having lower than excepted fitness in mixed HIV-1 competitions. However, there was a direct correlation (p = 0.0044; Pearson correlations) in replicative fitness values of each T/F virus from the mixed infection of Th1 and Th17 compared to the replicative fitness from pairwise infections in the same cells (S13 Fig). Furthermore, there was an equal distribution of T/F HIV-1 with higher or lower fitness in the mixed versus pairwise infections regardless of transmission mode, indicating that PWID T/F HIV-1 did not have lower than expected fitness due to a probability bias.

**Fig 4:**
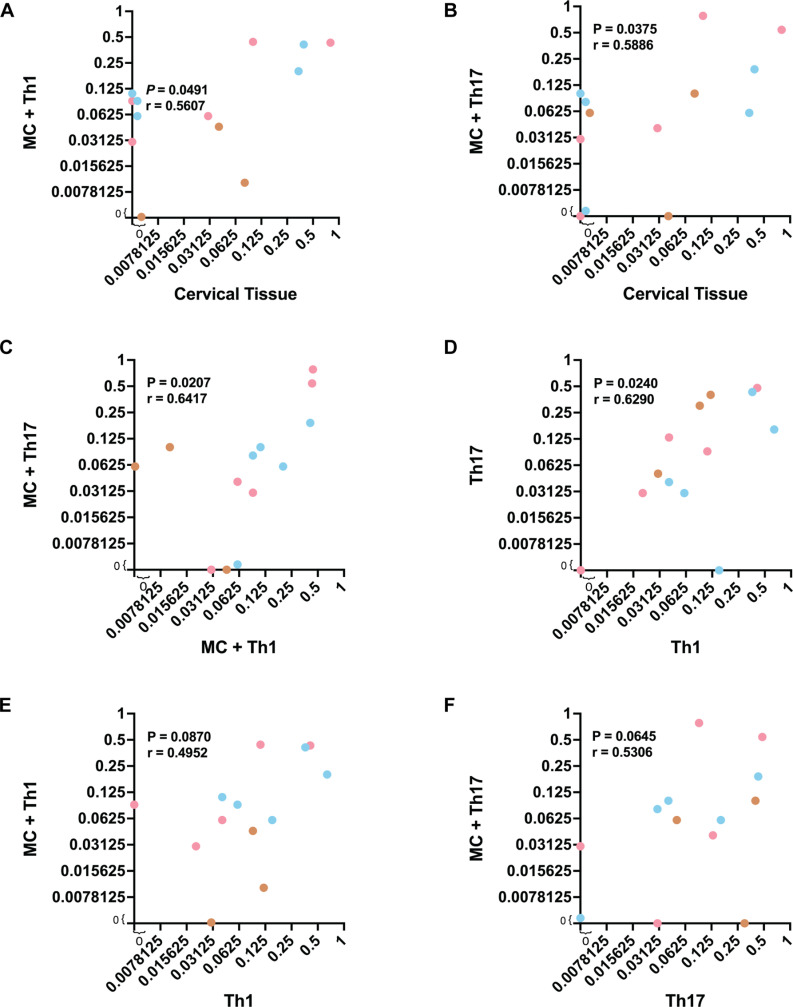
Correlation analysis on the average percent of fitness of chimeric T/F viruses. **(A-F)** Two-tailed Spearman correlation analysis on the average percent of fitness of chimeric T/F viruses in cervical tissue, migratory cells and Th1 co-culture (MC + Th1), migratory cells and Th17 co-culture (MC + Th17), Th1, and Th17.

Replication efficiency of T/F HIV-1 in cervical tissue, a surrogate of the primary infection site, showed a significant positive correlation with the replicative fitness in MCs coming from the same cervical tissue and co-cultured with Th1 or Th17 cells, a precursor of HIV-1 translocation and dissemination (Fig 4A-C). T/F HIV-1 replication efficiency in Th1 or Th17 alone also showed a positive correlation (Fig 4D). There was a trend for correlation when comparing the T/F HIV-1 fitness in the cervical MC + Th1 co-cultures and that in the Th1 alone. This same trend was observed between the T/F HIV-1 fitness in the cervical MC + Th17 and Th17 cultures (Fig 4E-F). These findings suggest that our ex vivo model of transmission (based on primary genital mucosal tissue) and replicative fitness (based on T cell and macrophage cultures) are uncoupled and governed by different parameters as previously described [1,2].

### Avidity for CD4 and CCR5 receptors for host cell entry by T/F HIV-1

**Fig 5:**
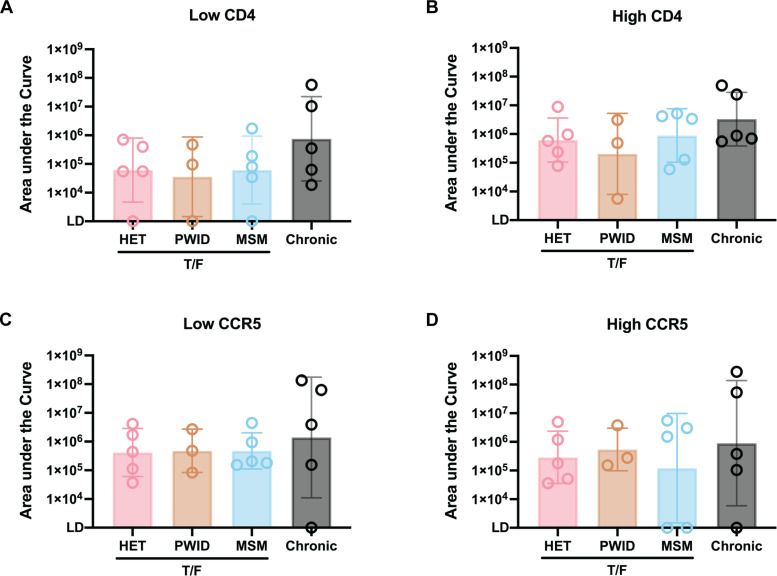
Evaluation of virus CD4/CCR5 utilization using Affinofile cells. Affinofile cells were induced with varying concentrations of minocycline (ranging from 0 to 20 ng/mL) and ponasterone A (ranging from 0 to 1 µM) to express varying levels of CD4 (approximately 850 to 125000 molecules per cell) and CCR5 (approximately 1274 to 15830 molecules per cell) independently and simultaneously. The infectivity was evaluated by luciferase activity expressed in Affinofile cells upon infection and the CD4/CCR5 response curves were characterized. **(A-D)** The mean area under the curve was calculated for each virus and grouped by transmission modes under low and high CD4 (approximately 100000 molecules per cell for low level; approximately 125000 molecules per cell for high level) and CCR5 (approximately 1296 molecules per cell for low level; approximately 15830 molecules per cell for high level) expression levels. Statistical analysis was performed using one-way ANOVA. LD, limit of detection.

Next, we examined various phenotypic and genotypic properties of the T/F HIV-1 that could be associated with transmission fitness and/or replicative fitness in T cells. Relative in vitro infectiousness of wild type HIV-1 isolates, especially Env chimeric HIV-1 with the same backbone is largely dependent on the usage of cellular receptor (CD4) and coreceptor (CCR5 or CXCR4) for efficient host cell entry [87,88]. In this study, all T/F HIV-1 variants were CCR5-tropic consistent with the finding that 98% of all T/F HIV-1 being CCR5 tropic [3]. HET-B4 was dual tropic, using either CCR5 or CXCR4. Affinofile cells were used to determine CD4 and CCR5 affinity and avidity related to host cell entry as previously described [57,89]. Different concentrations of minocycline and Ponasterone A were added to varying surface CD4 and CCR5 levels on HEK-293 cells (S14 and S15 Figs). Upon induction, a set of these Affinofile cells with different levels of cell surface CD4 and CCR5 were exposed to each of the 13 T/F HIV-1, and the infectivity was assessed by luciferase activity as described [57,90]. Infection and propagation of HIV-1 in the 293 Affinofile cells produce HIV-1 Tat, which transactivates HIV-1 long terminal repeat (LTR), leading to the expression of luciferase [91]. When comparing the chimeras from T/F versus chronic HIV-1 Env in these NL4–3 backbones, we observed that infection by chimeric T/F HIV-1 was more dependent on higher CD4 levels on the cell surface while able to scavenge for lower CCR5 (Fig 5A-D), consistent with previous studies [57]. For T/F HIV-1 from PWID, captured by circulating DCs followed by trans-infection to T cells may diminish the dependency on higher CD4 affinity during primary infection [26]. These results suggest that CD4 and CCR5 affinity and avidity may contribute to the transmission fitness differences between T/F and chronic HIV-1.

### T/F viruses from HET showed faster entry speed and higher sensitivity to Env conformation change

**Fig 6:**
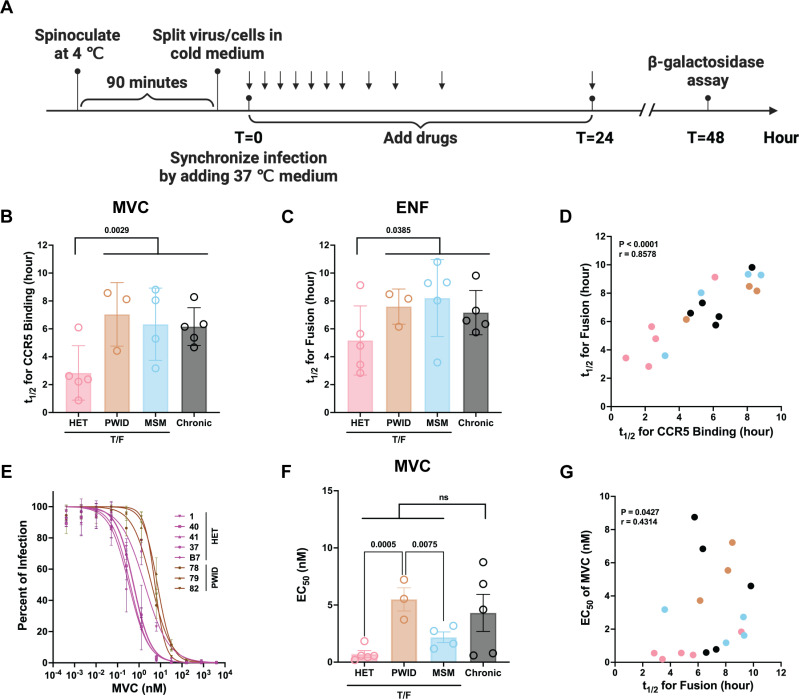
Virus entry kinetics and sensitivity to maraviroc. **(A)** Schematic representation of entry kinetics assay (created with BioRender.com). TZM-bl, a reporter cell line [98], was spinoculated with the virus at a multiplicity of infection (MOI) of 0.1 at 1200 × g for 90 minutes at 4 °C (a temperature that does not permit virus entry). Infections were synchronized by adding medium at 37 °C. Maraviroc (MVC) or enfuvirtide (ENF) at 10 µM was added at different time intervals (0, 1, 2, 3, 4, 5, 6, 8, 12, and 24 hours post-infection). The infectivity was measured 48 hours post-infection by β-Galactosidase expressed by TZM-bl cell line. **(B and C)** The half-maximal infection time (t_1/2_) against MVC or ENF was calculated for each virus and plotted according to transmission groups. **(D)** Two-tailed Spearman correlation analysis between t_1/2_ against MVC and ENF. To assess the MVC sensitivity of HIV-1, five-fold serial diluted MVC (ranging from 40 µM to 0.4 pM) was added to TZM-bl 2 hours before infection. The virus was added at an MOI of 0.05 and incubated with cells for 48 hours. The infectivity was measured by β-Galactosidase activity, and the dose-response curve to MVC was characterized. **(E)** The MVC response curves of HET and PWID chimeras. **(F)** EC_50_ against MVC for each virus was calculated based on the response curves and plotted by risk groups. **(G)** One-tailed Spearman correlation analysis between EC_50_ of MVC and t_1/2_ of fusion. One-way ANOVA followed by Tukey’s multiple comparisons and two-tailed unpaired t-tests were performed on B, C and **F.**

Faster entry speed might increase the transmission efficiency and also increase the chance of survival of a specific T/F HIV-1 clone from the inoculum HIV-1 quasispecies. By adding entry inhibitors, maraviroc (MVC) or enfuvirtide (ENF) at different times at the start of the infection cycle (Fig 6A), we calculated t_1/2_ for HIV-1 to bind CD4 and to be ready for CCR5 engagement (Fig 6B) and the t_1/2_ for HIV-1 to bind both CD4/CCR5 and initiate the cell-virus membrane fusion step (Fig 6C), respectively. Based on the time for MVC and ENF inhibition, HET chimeric T/F HIV-1 engaged CCR5 binding and mediated virus-cell membrane fusion (respectively) significantly faster than the combined group of PWID, MSM, and chronic chimeras. There was a strong direct correlation between rates for these two entry steps (Fig 6D). Along with a faster rate of host cell entry, the HET chimeras were exquisitely sensitive to MVC inhibition while the PWID and chronic chimeras were the least sensitive to MVC inhibition (Fig 6F). The drug susceptibility assays were performed on TZM-bl cell line but previous studies showed differences in MVC susceptibility between different HIV isolates when using different cell lines and primary T cells [92–96]. There was a weak positive correlation indicating that faster rates of host cell entry correlated with reduced sensitivity to MVC inhibition (Fig 6G), consistent with previous studies where wild type, chronic HIV-1 isolates with reduced MVC sensitivity show a weak correlation to faster host cell entry [71]. However, it is important to note that this correlation is not absolute with many MVC-resistant HIV-1 having faster rates of host entry and replicative fitness [96,97]. These findings highlight the potential role of virus entry in shaping transmission fitness.

### N-linked sites and glycosylation of HIV-1 T/F viruses

T/F and chronic viruses are believed to have different glycosylation profiles. T/F viruses exhibit lower glycosylation levels compared to chronic viruses [59,99–101]. We compared the predicted number of potential N-linked glycosylation sites (PNGS) on HIV-1 Env gp140 with the transmission fitness data (S16 Fig). Despite the ectodomains of the HET T/F Env having more PNGS sites than of the PWID T/F Env (p = 0.0462, Kriskal-Wallis), numbers of PNGS in the T/F Env did not appear associated with transmission fitness in a Spearman rank correlation. This suggests that if there are glycosylation differences between these viruses, it may be more related to the Env conformation and the accessibility to the glycosylation machinery than to the PNGS based on Env nucleotide sequences [102,103].

**Fig 7:**
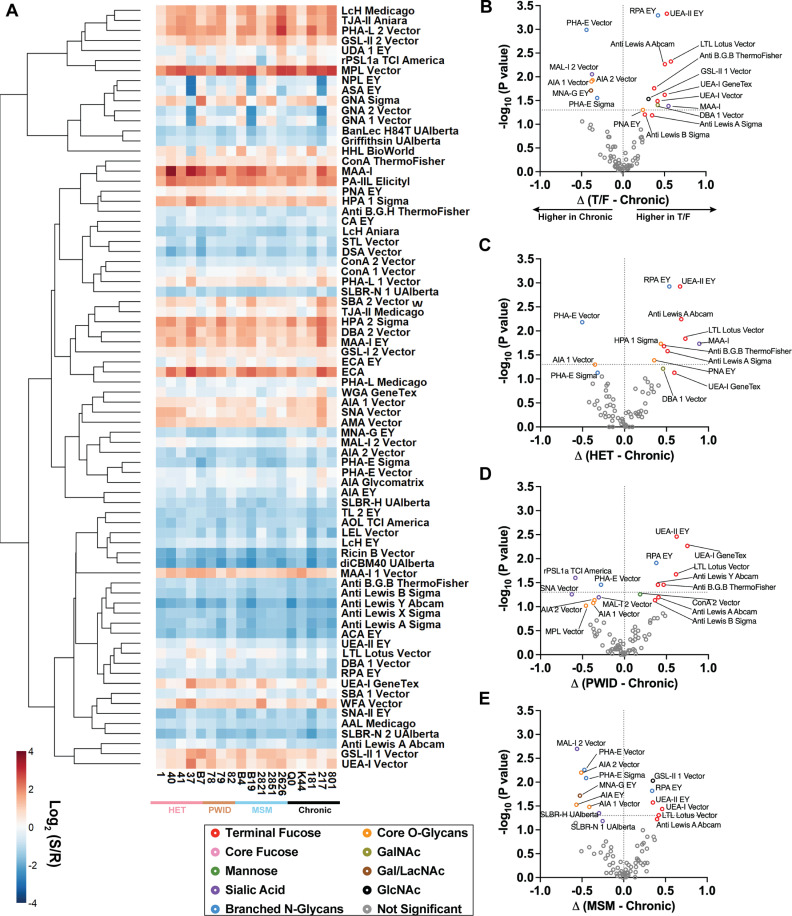
Lectin microarray analysis of T/F and chronic HIV-1. **(A)** A heat map generated from dual-colour lectin microarray analysis, with lectins clustered by Pearson’s correlation. The colours represent the normalized log₂ ratios of (Sample signal **(S)**/Reference signal **(R)**). Each virus was propagated 3 times separately on the U87.CD4.CCR5 cell line as individual samples and the mean values are shown. **(B-E)** Mann-Whitney test comparing lectin microarray results of T/F and chronic viruses. The X-axis shows the log₂(S/R) difference between the two transmission groups, and the Y-axis shows the -log₁₀(P value) from the Mann-Whitney tests. The dotted line on the Y-axis indicates a significance threshold of P = 0.05.

We have previously demonstrated a negative correlation between binding affinity to DC-SIGN and replication fitness in tissue, thereby linking transmission fitness to C-type lectins and glycosylation [24]. To further explore the impact of glycosylation on virus transmission, Env from lysed T/F and chronic HIV-1 were analyzed using dual-colour lectin microarray technology [104–106]. With this array, glycosylation profiles on different HIV-1 Env can be compared using panels of lectins and antibodies that bind to distinct glycan epitopes, including both N- and O-linked glycans [104–106]. These lectin arrays have been used in several previous studies to analyze HIV-1 glycosylation [107,108] and have the advantage of utilizing small amounts of material to test a multitude of lectins that bind to different glycan epitopes simultaneously. While this analysis does not identify specific glycan composition at each N-linked site in Env as can be obtained with mass spectrometry (MS), it will identify the relative amounts of glycans on the Env glycoproteins from multiple viral preparations (Fig 7A).

To determine possible differences in glycan binding with each of the 76 lectins and antibodies, we performed the Kruskal-Wallis statistical test (comparison between multiple groups) to compare between T/F HIV-1 derived from different modes of transmission (S17 Fig) and Mann-Whitney to compare between T/F viruses versus chronic viruses (Figs 7B-E and S17 and S18). Based on the lectin microarray analysis (Kruskal-Wallis and Mann-Whitney) shown in the volcano plot (S17 Fig), the binding to the array (which reflects the glycosylation profiles) did not differ significantly within the T/F Env groups (MSM, HET, or PWID). However, significant differences were observed in the glycosylation on T/F Env (from all transmission modes) when compared to chronic HIV-1 Env (Fig 7B-E). Compared to chronic Env’s, T/F Env’s had lower levels of the N-glycans containing bisecting GlcNAc (schematic of lectins, S19 Fig) binding by lectin PHA-E (blue circles, Fig 7B-E) and core 1/3 O-glycans (schematic of lectins, S19 Fig) binding to lectins AIA and MPL (orange circles, Fig 7B-E). Fucosylated glycans (schematic of lectins, S19 Fig) were increased on T/F viruses when compared to chronic strains, based on the binding of Lewis structures by LTL and anti-Lewis A/B/Y antibodies (red circles, Fig 7B-E). Increased terminal ɑ1,2 fucose (schematic, S19 Fig), which is recognized by UEA-I and anti-blood group B antibody (red circles, Fig 7B-E), was also enriched on T/F versus chronic Env. As described below, increased fucosylated glycans were associated with increased T/F HIV-1 replication during the passage from cervical migratory cells (e.g. DCs and LCs) to CD4 + T cells. DC-SIGN and langerin, C-type lectins on DCs and LCs have specificity for both mannose and fucose residues on glycans [109–111]. We did not find significant differences in oligomannose N-glycan (green circles) binding between chronic or T/F Env’s, which aligns with previous findings that oligomannose-type N-glycans are universally present on HIV-1 Env [102].

**Fig 8:**
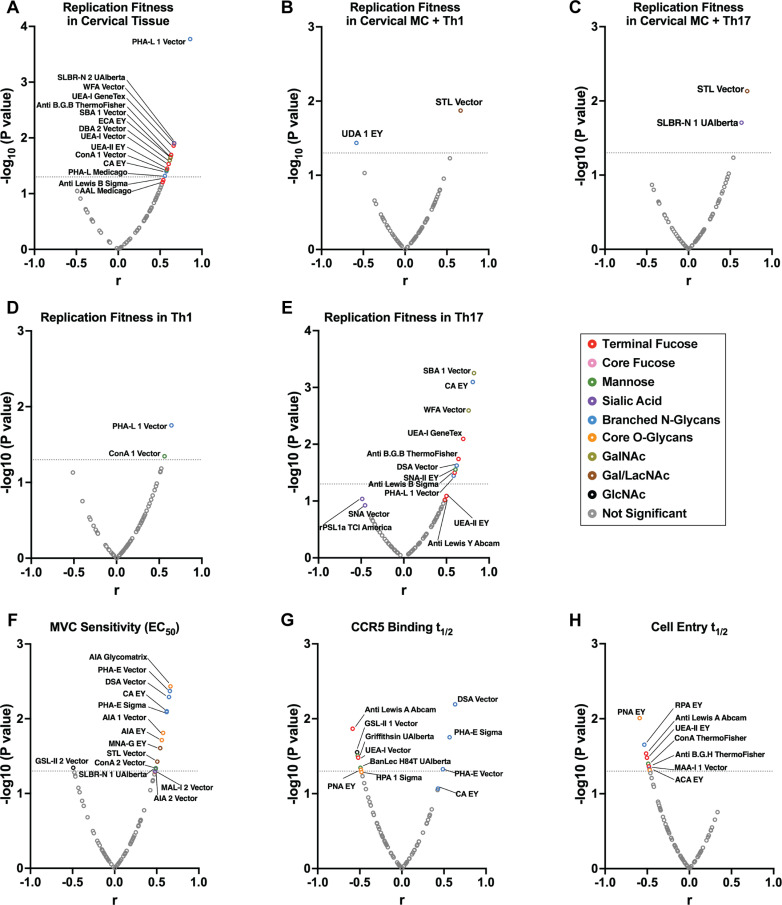
Correlation analysis of lectin microarray data. **(A-H)** Two-tailed Pearson correlation analysis between lectin microarray results and HIV-1 phenotypic characteristics tested above. The x-axis represents the r value from correlation analysis, and the y-axis represents the -log₁₀(P value) of correlation analysis. Panels A-E include transmission fitness data from all 13 T/F HIV-1 chimeras, while panels F-H include phenotypic data from 13 T/F and 5 chronic HIV-1 chimeras studied. The dotted line indicates a significance threshold of P = 0.05. Corresponding two-tailed Spearman correlation analysis is given in S20 Fig.

The glycan composition on the HIV-1 Env (based on lectin array results) was next compared to the various phenotypic characteristics of the viruses tested earlier in this study (as shown in Fig 3-6) using two-tailed Pearson analysis (Figs 8 and S21 and S23) and two-tailed Spearman analysis (S20 and S22 Figs). The T/F HIV-1 with the highest transmission fitness in cervical tissue had higher levels of ɑ1,2 fucosylated glycans on their Env’s (glycan schematic, S19 Fig) bound by lectins/antibodies UEA-I, UEA-II, anti-blood group B antibody, and anti-Lewis B antibody (red circles; Fig 8A). Example plots of T/F HIV-1 replicative fitness versus their fucosylated glycan composition are shown in S23 Fig. Higher levels of β-1,6 branched N-glycans (lectin: PHA-L, blue circle; Fig 8A) are also observed. As discussed below, T/F HIV-1 Env with more complex Env glycans (derived from the late Golgi apparatus) replicate better in the cervical tissue (Fig 8A). More “host-like” complex glycans on T/F Env could help to avoid binding by soluble mannose-binding lectins (MBL), resident at a high extracellular concentration in these genital mucosal tissues and thought to act as a barrier to prevent microbial translocation [112–114]. Furthermore, increased levels of N- or O-linked fucosylated glycans (binding by anti-Lewis A/X antibodies, anti-blood group H antibody, and lectin UEA-II; red circles) on these T/F Env’s positively correlated with the speed of host cell entry (cell entry t_1/2_, Fig 8H). T/F HIV Env with N-glycans containing polyLacNAc (lectins: DSA, STL, blue circles, Fig 8F, glycan types, S19 Fig) or bisecting GlcNAc (lectin: PHA-E, blue circles, Fig 8F) was correlated with decreased sensitivity to MVC (MVC sensitivity; Fig 8F) and slower entry kinetics (CCR5 binding t_1/2_; Fig 8G). As described in Fig 7B, chronic Env has higher levels of bisecting branched N-glycans, which would be found on the PNGS near conserved Env regions, obscuring antibody recognition of the sites necessary for receptor binding. However, these same bulky glycans on chronic Env are also thought to impact binding affinity to the receptors and potentially reduce host cell entry efficiency [115]. These findings suggest that glycan profiles may play a critical role in HIV-1 entry and transmission.

## Discussion

Transmission bottlenecks lead to a major decrease in virus population diversity during HIV-1 transmission. However, it remains poorly understood if the bottleneck is caused by a stochastic event or if the HIV-1 clone establishing primary systemic infection has special traits. Additionally, it is unknown if HIV-1 isolates with high transmission fitness from the same or different transmission routes share similar characteristics [116]. It is important to note the imbalance of the mode of transmission in different regions around the world, also predominated by different subtypes. North America is one of few regions in the world with a largely monophyletic HIV-1 epidemic caused by HIV-1 subtype B [117,118] transmitted primarily in MSM, but also with substantial numbers of HET and PWID transmission by the same HIV-1 subtype B. In contrast, the majority of new infections in sub-Saharan Africa are related to HET contact, with many cases reported from MSM and PWID transmission as well. Although HIV-1 subtype C predominates in southern African countries, nearly all HIV-1 subtypes and recombinant forms co-circulate in the central region of Africa, with defined boundaries of HIV-1 subtype distribution. This study examined the transmission fitness of T/F HIV-1 derived from different modes of infection derived from individuals with acute/early HIV-1 subtype B infections in North America.

In this study, we generated chimeric viruses by incorporating the Env of T/F HIV-1 from different transmission risk groups and evaluated their transmission fitness using multi-virus competitions on human cervical tissues, which includes translocation from the tissue via migrating cells (e.g. DCs) to infect exogenously added CD4 + Th1 or CD4 + Th17 cells. This ex vivo cervical tissue-based transmission assay serves as a surrogate of mucosal transmission, especially when comparing HET T/F HIV-1 (derived from male-to-female infections) to T/F HIV-1 transmitted during vaginal exposure. However, there are potential similarities in cervical and colorectal transmission of HIV-1. Topically applied T/F HIV-1 is also absorbed into colorectal explants leading to uptake of the virus by DCs, LCs, and macrophages in the lamina propria and conjugation with resident CD4 + T cells [54]. DCs also traffic HIV into local mucosal lymphoid follicles in the gut [54]. In cervical mucosal tissue compared to the gut or vaginal mucosa, lymphoid follicles especially tertiary lymphoid follicles [119–121] with most antigens (or HIV as antigen) captured and carried by antigen-presenting cells, macrophages and DCs, for presentation (or for HIV-1 trans-infection of T cells) in draining inguinal and iliac lymph nodes [120,122,123]. We found that T/F viruses from HET exhibited significantly higher transmission fitness on cervical tissues, MC + Th1 and MC + Th17, compared to T/F viruses from PWID. However, this increased fitness of HET in the transmission model was not evident in direct competitions/replication in Th1 and Th17 cell lines where the PWID-derived HIV-1 had higher replication capacity. We then investigated multiple phenotypic characteristics conferred by the different T/F Env’s in these viruses. T/F HIV-1 from PWID were the least sensitive to MVC inhibition, suggesting an enhanced ability to utilize diverse CCR5 conformations and potentially a greater usage of low CCR5 levels on the cell surface. These observations support our hypothesis that the interaction with CD4 receptor and CCR5 coreceptors [57] could be a key selection factor that affects transmission fitness. Our data suggests that the PWID T/F HIV-1 have high binding affinity/avidity to CD4/CCR5 and efficient host cell entry. This trait in PWID T/F HIV-1 may have been selected as a means to directly infect activated Th1 and Th17 cells (necessary in blood transmission), thereby reducing the need for trans-infection of Th1 and Th17 cells mediated by DC/LC/MO (and possibly specific glycan content on Env). However, other phenotypic properties of the T/F HIV could have an impact on transmission fitness. For example, resistance to interferons (e.g., alpha) and reduced ability to induce interferon-stimulated genes by T/F Env’s may also play a role in transmission [124,125]. However, there is also evidence that sensitivity to interferon-alpha is not significantly different between T/F and non-transmitted variants, suggesting that resistance to interferon-alpha may have minimal involvement in the selection of the T/F HIV-1 from the inoculating quasispecies, at least with subtype C infections [6].

Depending on the initial site of virus contact establishing primary infection, a specific trait may have a greater impact on which specific HIV-1 clone in the inoculating quasispecies establishes primary infection. It is also important to note that not every T/F HIV-1 with more or less efficiency with one trait (e.g., CD4 affinity) shows the same observation with another trait (e.g., CCR5 affinity). As described herein, we suspect that the transmission fitness is a multi-trait phenotype that differs depending on the origin of the T/F HIV-1 and the receptacle for infection. HIV-1 transmission fitness during male-to-female and male-to-male sexual transmission is assessed through the HIV-1 genetic characterization in donor and recipient blood near the time of infection. However, this transmission event is shaped by various transmission bottlenecks [1,5,126] occurring first in the donor and then in the recipient, e.g., semen to vaginal/cervical (or rectal mucosal) tissue followed by translocation from mucosal tissue to an infection foci, likely surrounding an HIV-specific germinal center, and then eventually spread through primary lymphoid tissue to establish systemic infection [1]. Receptor/co-receptor binding, rate of host cell entry, and glycan content appear to all have roles throughout this process of primary infection, which can be defined as transmission fitness.

As previously reported [1,2], the virus ultimately responsible for systemic primary infection will encounter different barriers along the way. In male-to-female primary infection, there is evidence of several foci of infection in the vaginal tract, each established by a divergent HIV-1 clone from the inoculum [17]. Recent findings suggest that most HIV-1 clones from the donor semen inoculum are trapped and prevented from penetrating the vaginal/cervical mucosa, which may be governed by C-type lectins binding to and trapping HIV-1 Env with high mannose glycans [24]. In the donor quasispecies, only a few HIV-1 clones will have an Env with complex N-glycans, resembling those glycans in human glycoproteins, allowing for some HIV-1 clones to escape the lectin trap. Soluble, extracellular MBLs are present in the mucosal layer of the human genital tract, serving to prevent the translocation of pathogens or even microbiomes through the mucosal layer [24,127–129]. If these pathogens escape the mucosal lectin trap and other innate barriers (e.g., mucin [1]), DCs and LCs expressing C-type lectin receptors (such as DC-SIGN) with specificity to high and oligomannose type N-glycans (more microbial-like) and fucosylated/complex glycans (more host like) [110,130–132], will bind HIV-1 Env for endocytosis/phagocytosis. Uptake of HIV-1 particles through DC-SIGN leads to lysosomal degradation for MHC class I and II antigen presentation but can also “store” HIV-1 in multivesicular bodies or invaginations which can result in highly efficient trans-infection from DCs to CD4 + T cells [44,45,133]. Thus, we propose that more high/oligomannose type and/or fucosylated/complex glycans (more host-like) on T/F HIV-1 Env compared to chronic HIV-1 Env may enhance affinity for lectin receptors on DCs and LCs (such as DC-SIGN), thereby increasing trans-infection efficiency. It is important to note that the pattern of specific glycan composition on specific N-linked sites on HIV-1 Env (not described herein) may also influence its affinity for lectin receptors on DCs and LCs.

Regardless of the mode of HIV-1 transmission, it is interesting that both MBL and DC-SIGN may play a role in transmission. Based on our findings herein, it is possible that blood DCs and MBLs still play some role in HIV-1 transmission but that efficiency of binding and entry into host cells is a more dominant trait. These observations are supported by several distinct studies. First, with PWID, the T/F HIV Env still bears glycan content more similar to HET and MSM T/F HIV Env (complex, fucosylated N- and O-glycans) than to the glycan content on the Env of chronic HIV-1 (Figs 7D and S18). Second, genetic polymorphisms leading to lower circulating MBL levels increase transmission efficiency and disease progression, suggesting less HIV-1 trapping in blood and other fluids [134]. Third, HIV-1 captured in blood by DCs (via DC-SIGN) and transported to lymph nodes for antigen presentations and trans-infection of T cells results in faster propagation and dissemination of infecting HIV-1 than direct HIV-1 infection of circulating CD4 + T cells [135]. Finally, unlike those T/F HIV-1 Env from HET and MSM, the PWID T/F HIV-1 Env are more tolerant of CCR5 conformational change (based on decreased susceptibility to MVC) and faster entry kinetics suggesting that DC capture mechanism may be a more important trait for transmission/propagation as observed with those HIV-1 found in established, chronic infection.

The presence of O-glycans on HIV-1 Env remains controversial [108,136–138]. Some studies have shown that O-glycans are absent on gp120 from HIV-1 virions and therefore are not important for the HIV-1 life cycle [137,139,140]. However, recent studies using lectin binding and MS have confirmed the presence of O-glycans on HIV-1 and have demonstrated that the presence of O-glycans promotes viral escape from broadly neutralizing antibodies [108,136]. Our lectin microarray data suggests different types and levels of O-glycans on T/F HIV-1 Env as compared to that on chronic HIV-1 Env. Notably, core 1/3 O-glycans (bound by AIA, MNA-G, MPL) are higher in chronic HIV-1 Env and correlate to decreased sensitivity to MVC (Figs 7B and 8F). These observations support future studies on the role of O-glycans on HIV-1 Env on immune evasion and transmission.

Finally, all T/F HIV-1 strains in this study were produced from a cell line and not from the actual tissue containing the inoculating virus in the case of penile-to-vaginal transmission (HET). PCR and next-generation sequencing technologies are capable of defining the exact T/F HIV-1 env gene sequences during transmission, predicting the T/F Env amino acid sequence, model the T/F Env structure, as well as determining the number and position of N-linked sites that would carry glycans on the T/F HIV-1. We and others have done all of this and yet have to define a specific genotype or structure of Env (except the loss of N-linked sites) that is associated with transmission and primary infection. However, few have tested the possibility of differential glycosylation on T/F HIV impacting mucosal transmission. Currently, there is no method to amplify the glycans on T/F HIV-1 Env directly from, e.g., cervical samples. Instead, T/F HIV-1 must be propagated on various cell lines or primary T cells to obtain sufficient Env to analyze glycan content by the lectin array analyses or by a combination of electron-transfer dissociation (ETD) and higher energy collisional dissociation (HCD) MS. Lectin array analyses are higher throughput, more quantitative, and provides a clear idea of O- and N-linked glycan types compared to ETD/HCD MS, which can identify specific glycans on specific N linked sites but is less quantitative, requiring more purified product and very intense analyses. We are currently developing methods for large-scale HIV-1 propagation of select HET T/F HIV-1 on human vaginal/cervical explant tissue and primary seminal T cells for analyses of glycan content on HET T/F HIV by both ETD/HCD mass spectrometry and lectin array analyses. Differential glycosylation of T/F and chronic Env in vivo reflects the Env primary sequence, secondary, and tertiary structures as well as the varying glycosylation machinery in the endoplasmic reticulum and Golgi apparatus of the producer cell (reflecting different expression levels of, e.g., various glycosyltransferases and glycosidases). However, regardless of the in vivo producer cells of HIV-1, this study suggests that during the processing of transmission, primary infection involves a filtering process to select for the HIV-1 Env with the “best” glycans in the inoculum of an HIV-1 quasispecies, which is highly diverse in Env genetic sequence and glycan composition. Based on the findings herein, the selected T/F HIV-1 variant from the inoculating quasispecies will have glycan composition on HIV-1 Env that promotes entry, propagation and dissemination in the entry tissue (e.g., cervical tissue) while also having the glycan content that evades innate defences and antigen recognition/presentation.

## Materials and methods

### Ethics statement and description of cervical tissue

Deidentified endocervical tissues were collected from women undergoing hysterectomies and frozen through the National Disease Research Interchange (NDRI). The use of these tissues was approved by the human ethics committee of Western University (WREM protocol 111054). Through NDRI, the tissue is dissected following a hysterectomy in the operating room and immediately frozen using a standard operating procedure regardless of the hospital site. NDRI obtains pathology reports and only sends out the tissue based on the specific criteria for this study (i.e., non-diseased tissue of pre-menopausal women). Following a temperature-controlled thaw of the tissue, we only employ tissue with >90–95% viability.

### Transmitted/Founder (T/F) and chronic chimeric viruses

Starting around 1996, the Division of AIDS, NIH, established the Acute Infection and Early Disease Research Network (AIEDRN) across the US with a similar network established by the Canadian government. AIEDRN provided anonymized samples from MSM and PWID participants and from female participants in the HET for this study (kindly provided by Drs. Martin Markowitz at the Aaron Diamond AIDS Research Center, Eric Rosenberg at the Massachusetts General Hospital, Eric Daar at UCLA, Julie McElrath at Fred Hutchinson Cancer Centre, Julio Montaner, BC Center for Excellence in HIV/AIDS, and from the Multicenter AIDS Cohort, NIH). Chimeric viruses were constructed using HIV-1 NL4–3 strain backbone and Env from subtype B T/F and chronic viruses derived from anonymous HIV-infected participants. The gp140 coding region of Env of T/F was RT-PCR amplified from RNA derived from plasma or serum from stored anonymized individuals drawn at acute/early HIV-1 subtype B infection with known modes of transmission. The Env from chronic viruses were previously PCR amplified from the plasma of chronically infected individuals in the absence of treatment [72,81,141]. All Env genes were amplified from viral RNA in samples of people living with HIV prior to antiretroviral therapy. A yeast-based recombination cloning method was used to construct these chimeric viruses (quasispecies were maintained this way), following a previously established protocol [70]. Briefly, the gp140 region of the *env* genes (encode the extracellular portion of Env) from T/F and chronic viruses were recombined into pREC_nfl_NL4–3_Δenv (ecto-MSD) vector following transfection into Saccharomyces cerevisiae. Virus production was achieved by transfection of these constructs, along with the supplementary vector pCMV_cplt, into HEK 293T cells, utilizing the Fugene 6 Transfection Reagent (Promega), following the manufacturer’s guidelines. All chimeric viruses were tested for tropism usage on U87.CD4 expressing CCR5 or CXCR4 [7]. Viruses were propagated and titrated on U87.CD4.CCR5 using 50% tissue culture infective dose (TCID_50_) assay. The DNA sequences of gp140 from chimeric viruses were aligned using the MAFFT program on the Los Alamos HIV database for a phylogenetic tree. The tree was generated using the TreeMaker tool (neighbour-joining way) on the Los Alamos HIV database and visualized by FigTree v1.4.4.

### Env quantification by Western blotting

To quantify the Env expression level, pellets were obtained by centrifugation of 10^5^ TCID_50_ values of chimeric viruses at 38,000 rpm for 90 minutes. The virus pellets were then resuspended in PBS and lysed with Tris-Glycine SDS sample buffer (LC2676, Invitrogen) and 2-Mercaptoethanol (Sigma-Aldrich). Samples were heated at 93°C for 15 minutes and then separated on Tris-Glycine 4–20% Mini Protein Gels (XP04200BOX, Invitrogen) at 125V for 90 minutes using Mini Gel Tank (Invitrogen). Samples were then transferred to 0.2 μm PVDF transfer membranes (Thermo Scientific) at 20V for 70 minutes. The blots were blocked with 5% milk and probed for p24 using HIV1 p24 Monoclonal Antibody (D45F) (MA1–7039, Invitrogen) at a dilution ratio of 1 in 25. The blots were then stripped using 2 M glycine (pH = 2.2) and probed again for Env using B13 hybridoma supernatant (the hybridoma was kindly provided by Dr. George Lewis, The Institute of Human Virology, Baltimore, MD, USA) [142]. The primary anti-gp120 B13 antibody used to probe the denatured Env binds to a linear epitope in gp120, which is conserved in all of these divergent subtype B Env’s [142]. Blots were incubated with horseradish peroxidase-conjugated Goat anti-Mouse IgG (H + L) Secondary Antibody (31430, Invitrogen) at a dilution ratio of 1:5000 and revealed by using Immobilon Crescendo Western HRP substrate (MilliporeSigma). Band intensities were quantified by ImageJ. Serial dilutions of lysed virus were initially probed on Western blots to obtain the linear range from a single gel.

### HIV-1 multi-virus competitions in human explant tissue

The competitions were performed as previously described [7,24]. Briefly, frozen human explant cervical tissue, including both epithelium and stroma, was thawed at room temperature and cut into 5mm^3^ pieces. Every 3 pieces of tissue from the same donor were cultured in one well (one competition) of a 48-well plate with RPMI-1640 medium supplemented with 10% heat-inactivated fetal bovine serum (FBS [Wisent Inc]), 100 units/mL Penicillin-Streptomycin (P/S [Gibco]), and 2.5 µg/mL amphotericin B (Sigma-Aldrich) (tissue culture medium). A mix of 6 chimeric T/F HIV-1 from different transmission routes, each at 3750 IUs, was added to one well containing the 3 pieces of tissue (one competition) and incubated for 6 hours. The tissue was then washed with PBS and cultured in fresh tissue culture medium at 37 °C with 5% CO_2_ for 10 days. MCs (around 2.5 × 10^4^ cells per 5 mm³ of tissue) that migrated out of the tissue were collected 48 hours post-infection by centrifugation at 1500 g for 5 minutes and co-cultured with 2.5 × 10^4^ human Th1 (PM1) or Th17 (A3R5.7) cell lines for 10 days in tissue culture medium at 37 °C with 5% CO_2_. In addition, the exact panel of virus mixtures (each at 125 IUs) was added to human Th1 (PM1) or Th17 (A3R5.7) cell lines directly for 6 hours then washed with PBS and cultured for 10 days. The competition panel was designed so that all T/F viruses can compete against each other at least once. DNA was extracted from tissue pieces, MC + Th1, MC + Th17, Th1 and Th17 using DNeasy Blood & Tissue Kit (Qiagen) following the manufacturer’s instructions. Each competition was performed in triplicate on tissue from 3 donors, and separately in triplicate on Th1/Th17 cell lines, repeated 3 times.

### PCR and deep sequencing analysis of multi-virus competition

The C2-V3-C3 region of the *env* was amplified from tissue, MC + Th1, and MC + Th17 DNA extracts by an external-nested PCR amplification. Primers used for the external PCR were EnvB and ED14, and those for nested PCR were E80 and E125 (see Table 1 for sequences). Target products were amplified from the viral (v)DNA using Platinum Taq DNA polymerase (Invitrogen) according to the manufacturer’s instructions. PCR products were confirmed on 1% agarose gel with bands of approximately 500 base pairs [17,24]. We utilized vDNA over vRNA to monitor mixed infections to avoid any remnant vRNA from the inoculum. vDNA is also indicative of infection and correlated with vRNA production in the supernatant.

**Table 1:** PCR primers for HIV-1 C2-V3-C3 region.

Primer/Probe	Sequences (5’ to 3’)
EnvB (forward)	AGAAAGAGCAGAAGACAGTGGCAATGA [HXB2 positions 6202 to 6228]
ED14 (reverse)	TCTTGCCTGGAGCTGCTTGATGCCCCAGAC [HXB2 positions 7932 to 7961]
E80 (forward)	CCAATTCCCATACATTATTGTG [HXB2 positions 6858 to 6879]
E125 (reverse)	CAATTTCTGGGTCCCCTCCTGAGG [HXB2 positions 7315 to 7338]
E80-illu (forward)	TCGTCGGCAGCGTCAGATGTGTATAAGAGACAGCCAATTCCCATACATTATTGTG
E125-illu (reverse)	GTCTCGTGGGCTCGGAGATGTGTATAAGAGACAGCAATTTCTGGGTCCCCTCCTGAGG

Forward primer E80-illu and reverse primer E125-illu (see Table 1 for sequences) containing Illumina tags followed by template-specific sequences were used to prepare the library for Illumina sequencing. PCR cycle conditions were 95°C for 2 minutes, followed by 35 cycles of 95°C for 30 seconds, 55°C for 30 seconds, and 72°C for 45 seconds, and a final extension of 72°C for 10 minutes. PCR products were then purified and amplified according to Illumina sample preparation instructions and were sequenced using a 600-cycle kit on an Illumina MiSeq instrument (Illumina). SeekDeep was used to analyze Illumina sequencing data [84]. The pipeline was adjusted to allow up to 6 mismatches for clustering sequences to account for virus mutations and sequencing errors.

### qPCR

An external HIV-1 gag-specific PCR was performed first using Platinum Taq DNA polymerase, forward primer 1D_SR-1 and reverse primer 4E_GA8 (see Table 2 for sequences). The conditions are as follows: 95°C for 2 minutes, followed by 20 cycles of 95°C for 30 seconds, 55°C for 30 seconds, 72°C for 1 minute 50 seconds, and a final extension of 72°C for 10 minutes. The PCR product was then incorporated into the qPCR reaction with forward primer SUB A/D 5LTR PA and reverse primer SUB A/D GagR (see Table 2 for sequences).

**Table 2:** Primers for qPCR.

Primer/Probe	Sequences (5’ to 3’)
1D_SR-1 (forward)	TAGACCAGATCTGAGCCTGGGAGC-3′ [HXB2 positions 457 to 490]
4E_GA8 (reverse)	TCCCATTCTGCAGCTTCCTCATTG-3′ [HXB2 positions 1404 to 1427]
SUB A/D 5LTR PA (forward)	CCCACTGCTTAAGCCTCAATAAAGC-3′ [HXB2 positions 510 to 534]
SUB A/D GagR (reverse)	AGCTCCCTGCTTGCCCATACTA -3′ [HXB2 positions 890 to 911]
MTS (forward)	AGCCACTTTCCACACAGACAT
MTA (reverse)	CTGGTTAGGCTGGTGTTAGGG

DNA extracts from tissue, MC + Th1 and MC + Th17 were diluted 1000 times first and then were subject to qPCR reaction with forward primer MTS and reverse primer MTA (see Table 2 for sequences) targeting human mitochondrion region as internal control to calculate HIV copy numbers per cell.

The qPCR reactions were performed using SYBR Green Master Mix (A46109, Applied Biosystems) on the QuantStudio5 real-time PCR system (Applied Biosystems) with conditions: 50 °C for 2 minutes, 95 °C for 2 minutes, and 40 cycles of 95 °C for 1 second and 55 °C for 15 seconds and 72 °C for 1 minutes. All samples were run in duplicate. Samples were considered negative when cycle threshold (CT) values were above 35.

### Affinofile system

pDM1.1, the plasmid designed to express firefly luciferase upon HIV infection, was transfected in Affinofile cells using Fugene 6 according to the manufacturer’s instructions. Affinofile cells were digested with trypsin and divided into two sets: one set was distributed into 6-well plates at a density of 10^6^ cells per well, intended for subsequent flow cytometry analysis, while the other set was placed in 96-well plates at a density of 2 × 10^4^ cells per well for infectivity analysis. Cells were culture in DMEM supplemented with 10% dialyzed FBS (Cat # A3382001, Gibco) and 50 µg/ml blasticidin S HCl (Cat # A1113903, Gibco) for 48 hours at 37 °C with 5% CO_2_. Affinofile cells were then subjected to a controlled induction regimen to modulate the expression levels of CD4 and CCR5. This induction was achieved by exposure to varying concentrations of minocycline ([ranging from 0 to 20 ng/mL], Cat # M9511-25MG, Sigma-Aldrich) and Ponasterone A ([ranging from 0 to 1 µM], Cat # H10101, Invitrogen) for a period of 24 hours, following established protocols [57,90,143].

Induced cells in 6-well plates were digested by enzyme-free cell dissociation buffer (Cat # 13151014, Gibco) and stained with PE-conjugated anti-human CD4 antibody (300508, Biolegend), APC-conjugated anti-human CCR5 antibody (359122, Biolegend) and Zombie Violet (423113, Biolegend) following the manufacturer’s instructions. Following staining, cells were fixed with 2% formaldehyde and then analyzed using an LSR II flow cytometer (BD Biosciences) to quantify the levels of CD4 and CCR5 expression, with mean fluorescence intensity measurements obtained and analyzed using FlowJo software.

Induced cells in 96-well plates were infected by viruses at MOI of 0.2 for 48 hours in DMEM supplemented with 10% dialyzed FBS. The infectivity was measured using the Luciferase Assay System (E2620, Promega) according to the manufacturer’s instructions.

### Entry kinetics assay

The assay was performed as described before [57]. Briefly, 5 × 10^5^ TZM-bl and viruses at MOI of 0.1 were spinoculated at 1,200 × g for 90 minutes at 4 °C and then split into 96-well plates (50 µL/well). Virus infection was synchronized by adding 130 µl of 37 °C DMEM complete medium and 20 µL MVC or ENF at 10 µM to each well at different time intervals (0, 1, 2, 3, 4, 5, 6, 8, 12 and 24 hours post-infection). The cells were then incubated for 48 hours at 37 °C with 5% CO_2_. The infectivity was measured using the Galacto-Star β-Galactosidase Reporter Gene Assay System (Invitrogen) according to the manufacturer’s instructions. Half maximal infection time (t½) was calculated using GraphPad Prism 9. β-Galactosidase activity at 24 hours post-infection was used as the maximal infection value.

### MVC sensitivity assay

10^4^ TZM-bl cells per well were seeded in 96-well plates using DMEM supplemented with FBS and P/S (DMEM complete medium). 5-fold serial diluted MVC (ranging from 40 µM to 0.4 pM) was added to cells and incubated for 2 hours at 37 °C with 5% CO2 prior to infection. Viruses were added to cells at a MOI of 0.05 and incubated with cells for 48 hours at 37 °C with 5% CO_2_. The infectivity was measured using the Galacto-Star β-Galactosidase Reporter Gene Assay System (Invitrogen) according to the manufacturer’s instructions. Half maximal effective concentration (EC_50_) was calculated using GraphPad Prism 9. β-Galactosidase activity with the lowest drug concentration was used as the maximal infection value.

### Lectin microarray

60 mL cell-debris-free virus supernatant was harvested and the ultracentrifuged at 37,000 g for 90 minutes. The pellet was resuspended in 300 µL 100 mM ammonium bicarbonate (pH = 8.0). A final concentration of 2.5 unit/mL Benzonase (E8263-5KU, Millipore Sigma), 2 mM MgCl, and 0.25% Empigen BB (30326–250ML, Millipore Sigma) was added, and the solution was centrifuged at 14,800 rpm for 15 minutes at 4°C to release viral Env from the membrane. The solution was replaced with PBS with 0.08% Triton by repeatedly filtering the lysate through 50 kDa centrifugal filter units (UFC505024, Millipore Sigma) for 4 times. 100 µL of the sample remaining in the filter units was collected and shipped to Lara K. Mahal’s lab at the University of Alberta for lectin microarray analysis following published procedures [104–106]. Details are in S1-2 Tables.

## Statistical analysis

Statistical analysis was performed on GraphPad Prism 9 or using R. The Kruskal-Wallis test followed by Dunn’s multiple comparisons test was applied to compare the average percent of fitness in multi-virus competitions. One-way ANOVA followed by Tukey’s multiple comparisons were employed to compare Env expression levels, MVC sensitivity, CCR5 binding speed, fusion speed, HIV copy numbers per cell and CD4/CCR5 utilization among virus groups from different transmission modes. Pearson and Spearman correlation analysis was conducted to determine the association between virus average percent of fitness in multi-virus competitions, the count of PNGS, MVC sensitivity, virus fusion speed and lectin binding affinities. Mann-Whitney test was used to compare the lectin binding affinities between different risk groups. P values smaller than 0.05 were considered significant.

## Supporting information

S1 Fig Original western blot. (TIF)

S2 Fig Nucleotide sequence alignment of T/F viruses. (PDF)

S3 Fig Amino acid sequence alignment of T/F viruses. (PDF)

S4 Fig Virus content from each competition group on cervical tissue. Illumina sequencing results from competitions were processed using SeekDeep and the content of each virus in each competition was calculated. “ND” indicates cases where no data was available.
(TIF)

S5 Fig Virus content from each competition group on migratory cells and Th1 co-culture. Illumina sequencing results from competitions were processed using SeekDeep and the content of each virus in each competition was calculated. “ND” indicates cases where no data was available.
(TIF)

S6 Fig Virus content from each competition group on migratory cells and Th17 co-culture. Illumina sequencing results from competitions were processed using SeekDeep and the content of each virus in each competition was calculated. “ND” indicates cases where no data was available.
(TIF)

S7 Fig Virus content from each competition group on Th1. Illumina sequencing results from competitions were processed using SeekDeep and the content of each virus in each competition was calculated.
(TIF)

S8 Fig Virus content from each competition group on Th17. Illumina sequencing results from competitions were processed using SeekDeep and the content of each virus in each competition was calculated.
(TIF)

S9 Fig Percent of replication on cervical tissue, migratory cells and Th1 co-culture, and migratory cells and Th17 co-culture in each competition group. **(A-C)** The average content of viruses from each risk group was calculated based on SeekDeep results. Viruses that participated in each competition group are indicated at the bottom of the figure. MC + Th1, migratory cells and Th1 co-culture; MC + Th17, migratory cells and Th17 co-culture.
(TIF)

S10 Fig Percent of replication on Th1 and Th17 in each competition group. **(A-B)** The average content of viruses from each transmission group was calculated based on SeekDeep results. Viruses that participated in each competition group are indicated at the bottom of the figure.
(TIF)

S11 Fig The average percent of fitness of cervical tissue competitions on each donor. **(A-F)** Grouped average percent of fitness in cervical tissue, migratory cells and Th1 co-culture (MC + Th1), migratory cells and Th17 co-culture (MC + Th17) from each donor. The Kruskal-Wallis test followed by Dunn’s multiple comparisons test was performed.
(TIF)

S12 Fig HIV-1 copy numbers in tissue and cells relative to mitochondria DNA in competitions. **(A-K)** qPCR was performed to evaluate HIV-1 relative copy numbers on DNA extracted from cervical tissue (~ 3 pieces of 5 mm^3^ tissue), migratory cells and Th1 co-culture (MC + Th1), migratory cells and Th17 co-culture (MC + Th17) in each donor and Th1 and Th17.
(TIF)

S13 Fig Correlation analysis between multivirus and pairwise competitions. **(A)** Pearson correlation analysis result between multivirus and pairwise competitions in Th1 or Th17. Dots represent data acquired from Th1 and triangles represent data acquired from Th17.
(TIF)

S14 Fig Response curves of T/F and chronic viruses to CD4 and CCR5 expression levels. Affinofile cells were induced with a range of concentrations of minocycline (from 0 to 20 ng/mL) and ponasterone A (from 0 to 1 µM) to independently and simultaneously express varying levels of CD4 (approximately 850–125,000 molecules per cell) and CCR5 (approximately 1,274–15,830 molecules per cell). Subsequently, these cells were infected with T/F viruses from different transmission modes or chronic viruses. Virus infectivity was assessed by measuring relative light units (RLU) from luciferase activity and plotted against the approximate CD4 **(A-H)** or CCR5 **(I-P)** expression levels, considering both low (approximately 100,000 molecules per cell for CD4; approximately 1,296 molecules per cell for CCR5) and high (approximately 125,000 molecules per cell for CD4; approximately 15,830 molecules per cell for CCR5) expression conditions. LD, limit of detection.
(TIF)

S15 Fig CD4 and CCR5 utilization efficiency of individual virus. Affinofile cells were induced with a range of concentrations of minocycline (from 0 to 20 ng/mL) and ponasterone A (from 0 to 1 µM) to independently and simultaneously express varying levels of CD4 (approximately 850–125,000 molecules per cell) and CCR5 (approximately 1,274–15,830 molecules per cell). Subsequently, these cells were infected with T/F or chronic HIV-1. **(A-D)** The area under the curve was calculated for each virus under low and high CD4 and CCR5 expression levels based on response curves in S14 Fig. LD, limit of detection.
(TIF)

S16 Fig The number of potential N-linked glycosylation sites (PNGS) on HIV-1 Env. **(A)** The PNGS on HIV-1 gp140 region. **(B)** Kruskal-Wallis analysis on PNGS in gp140 based on different transmission modes. **(C)** Kruskal-Wallis analysis on PNGS in Env variable regions based on different transmission modes. **(D-F)** Spearman correlation analysis between PNGS in gp140 and average percent of fitness during competitions. **(G-I)** Spearman correlation analysis between PNGS in Env variable regions and average percent of fitness during competitions. PNGS was predicted using the Los Alamos database.
(TIF)

S17 Fig Kruskal-Wallis and Mann-Whitney test results on lectin microarray between different T/F groups. Kruskal-Wallis **(A)** and Mann-Whitney **(B-D)** tests were performed on the log₂(S/R) values from the lectin microarray data. The x-axis in panels **B-D** represents the log₂(S/R) difference between the two transmission groups. The y-axis in panels **A-D** represents the -log₁₀(P value) from the Kruskal-Wallis or Mann-Whitney tests.
(TIF)

S18 Fig Table of Mann-Whitney test results on lectin microarray. Mann-Whitney tests were performed on the log₂(S/R) values from the lectin microarray data. The table displays all lectins that showed significant differences in the Mann-Whitney test based on the lectin microarray data. The names, types, and predominant binding motifs of the lectins are also provided in the table. The “Difference” column shows the log₂(S/R) difference between the two transmission groups (listed in the left column). The P-values were obtained from the Mann-Whitney test. Lectins highlighted in red indicate significantly higher binding avidity, while those in blue indicate significantly lower binding avidity in the comparison.
(TIF)

S19 Fig Schematic representation of lectins and their predominant binding motifs related to HIV-1 phenotypic traits. Abbreviations and symbols: Mannose (Man, green circles), N-acetylglucosamine (GlcNAc, blue squares), N-acetyllactosamine (LacNAc, blue square + yellow circle), fucose (Fuc, red triangles), galactose (Gal, yellow circles), N-acetylgalactosamine (GalNAc, yellow squares). R, the rest of the molecule. The figure was created with BioRender.com.
(TIF)

S20 Fig Two-tailed Spearman correlation analysis results between different phenotypic data tested above and lectin microarray results. The x-axis represents the r value from correlation analysis, and the y-axis represents the -log₁₀(P value) of correlation analysis. Panels A-E include transmission fitness data from all 13 T/F HIV-1 chimeras, while panels F-H include phenotypic data from 13 T/F and 5 chronic HIV-1 chimeras studied. The dotted line indicates a significance threshold of P = 0.05. The corresponding two-tailed Pearson correlation analysis is given in Fig 8.
(TIF)

S21 Fig Detailed table of two-tailed Pearson correlation analysis results between different phenotypic data tested above and lectin microarray results. The table displays lectins that reached statistical significance in the correlation analysis. The names, types, and predominant binding motifs of the lectins are provided, along with the r and P-values from the correlation tests. The types of phenotypic data tested in this study are listed in the left column. The corresponding data visualization is presented in Fig 8.
(TIF)

S22 Fig Detailed table of two-tailed Spearman correlation analysis results between different phenotypic data and lectin microarray results. The table displays lectins that reached statistical significance in the correlation analysis. The names, types, and predominant binding motifs of the lectins are provided, along with the r and P-values from the correlation tests. The types of phenotypic data tested in this study are listed in the left column. The corresponding data visualization is presented in S20 Fig.
(TIF)

S23 Fig Example plots of correlation between fucosylated lectin microarray data and HIV-1 T/F replication fitness in cervical tissue. The two-tailed Pearson correlation analysis was performed.
(TIF)

S1 Table Lectin microarray information. (PDF)

S2 Table Lectins used in microarrays. (PDF)
