# Correction: Transmitted/founder (T/F) HIV-1 derived from sexual contact exhibits greater transmission fitness in human cervical tissue than T/F HIV-1 from blood-to-blood contact: Unique glycan profiles on T/F envelopes associated with transmission phenotypes

**DOI:** 10.1371/journal.ppat.1013697

**Published:** 2025-11-18

**Authors:** Yiying Zhang, Katja Klein, Annette Ratcliff, Sashini Loku Galappaththi, Nicholas Hathaway, Nicholas Twells, Mukti Patel, Stephen Temesy, Jeffrey A. Bailey, Lara K. Mahal, Carole Creuzenet, Eric J. Arts

The Data Availability statement for this article is incorrect. The correct statement is: The authors confirm that all data underlying the findings are fully available without restriction. All relevant data are within the paper, its Supporting Information files and/or provided as Excel spreadsheets in the figshare repository (https://doi.org/10.6084/m9.figshare.30213652.v1).
