# Supplementary figures and images for: Transmitted/founder (T/F) HIV-1 derived from sexual contact exhibits greater transmission fitness in human cervical tissue than T/F HIV-1 from blood-to-blood contact: Unique glycan profiles on T/F envelopes associated with transmission phenotypes
Source: PLoS Pathog. 2025 May 23;21(5):e1013177. doi: 10.1371/journal.ppat.1013177 (PMC12140434; doi:10.1371/journal.ppat.1013177)

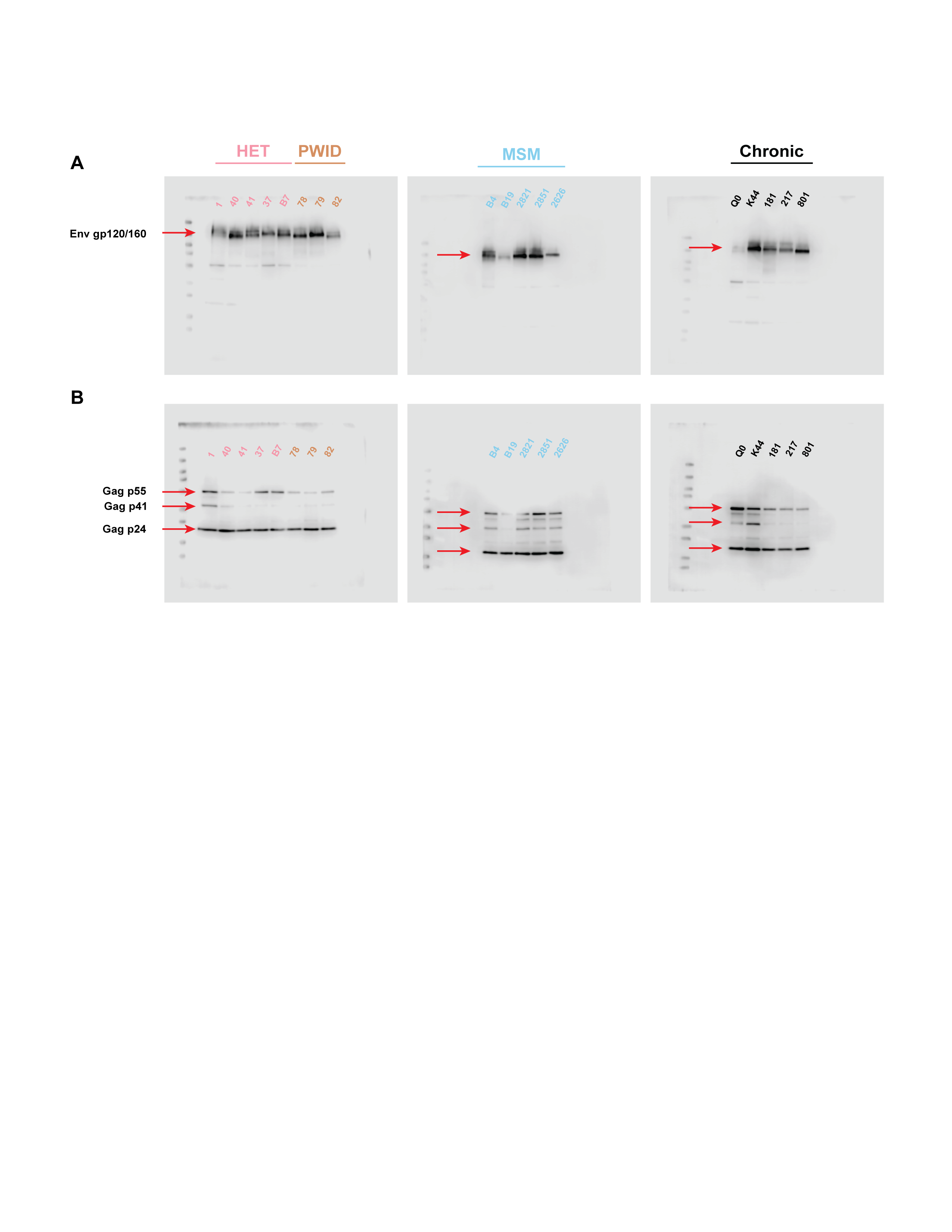

Supplement: S1 Fig — (TIF) [file ppat.1013177.s001.tif]

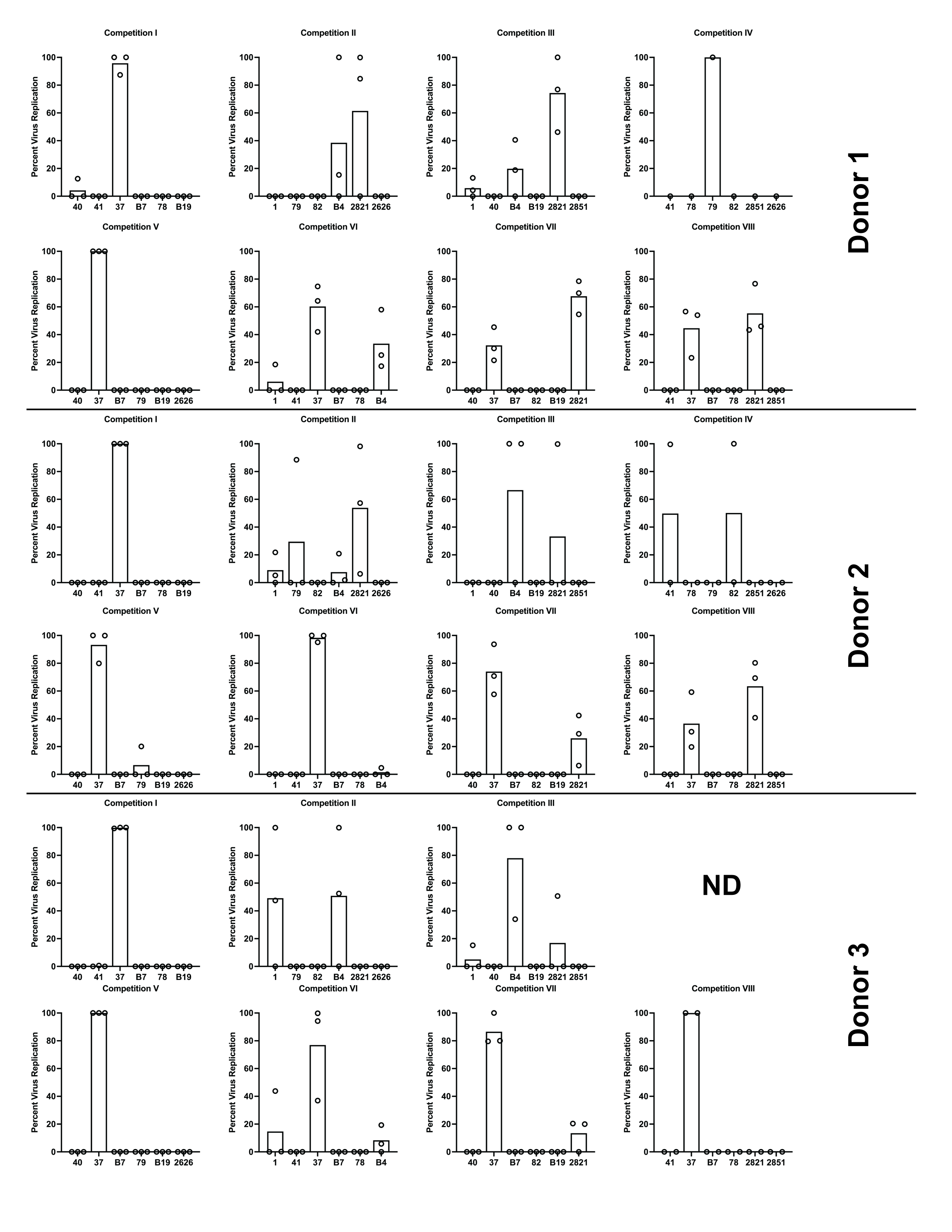

Supplement: S4 Fig — Illumina sequencing results from competitions were processed using SeekDeep and the content of each virus in each competition was calculated. “ND” indicates cases where no data was available. (TIF) [file ppat.1013177.s004.tif]

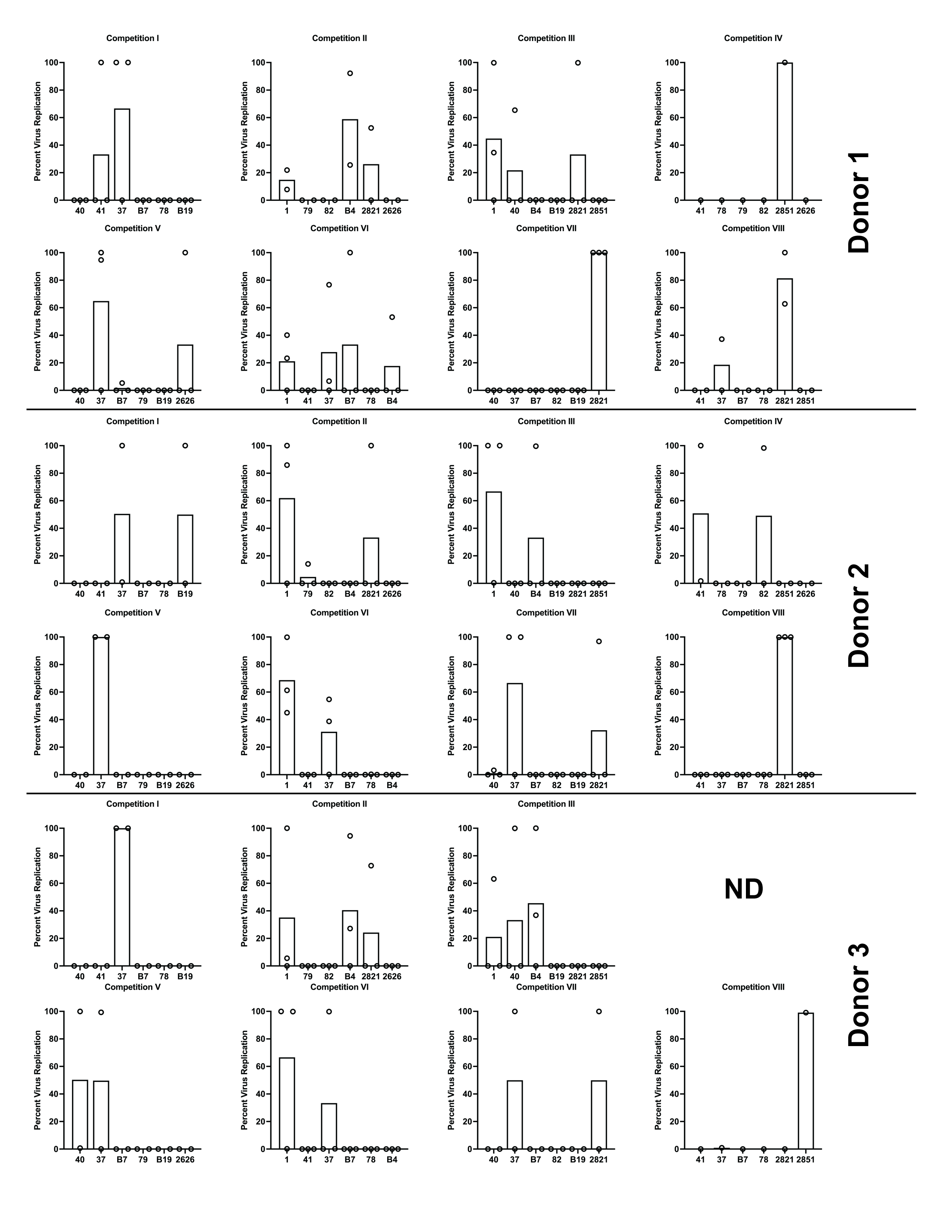

Supplement: S5 Fig — Illumina sequencing results from competitions were processed using SeekDeep and the content of each virus in each competition was calculated. “ND” indicates cases where no data was available. (TIF) [file ppat.1013177.s005.tif]

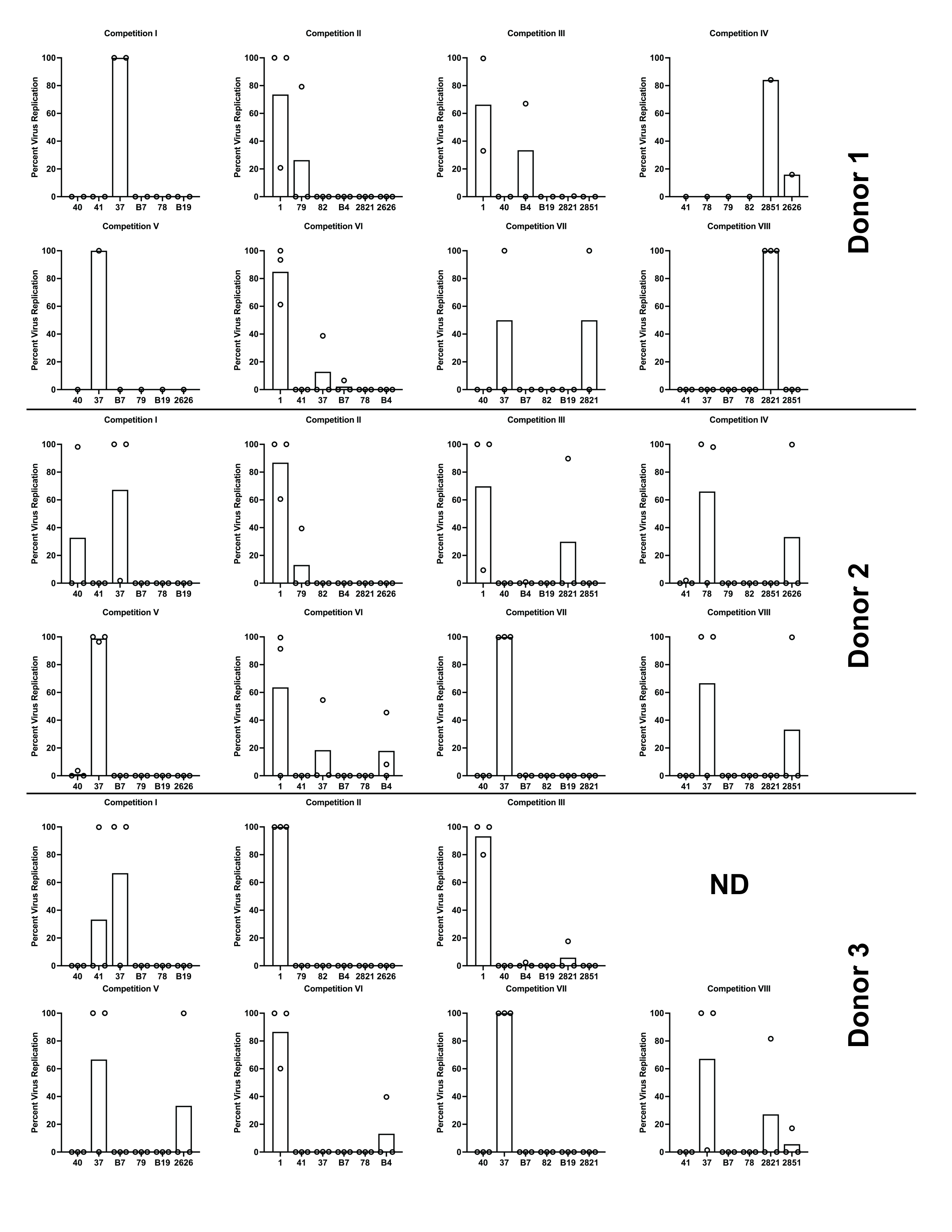

Supplement: S6 Fig — Illumina sequencing results from competitions were processed using SeekDeep and the content of each virus in each competition was calculated. “ND” indicates cases where no data was available. (TIF) [file ppat.1013177.s006.tif]

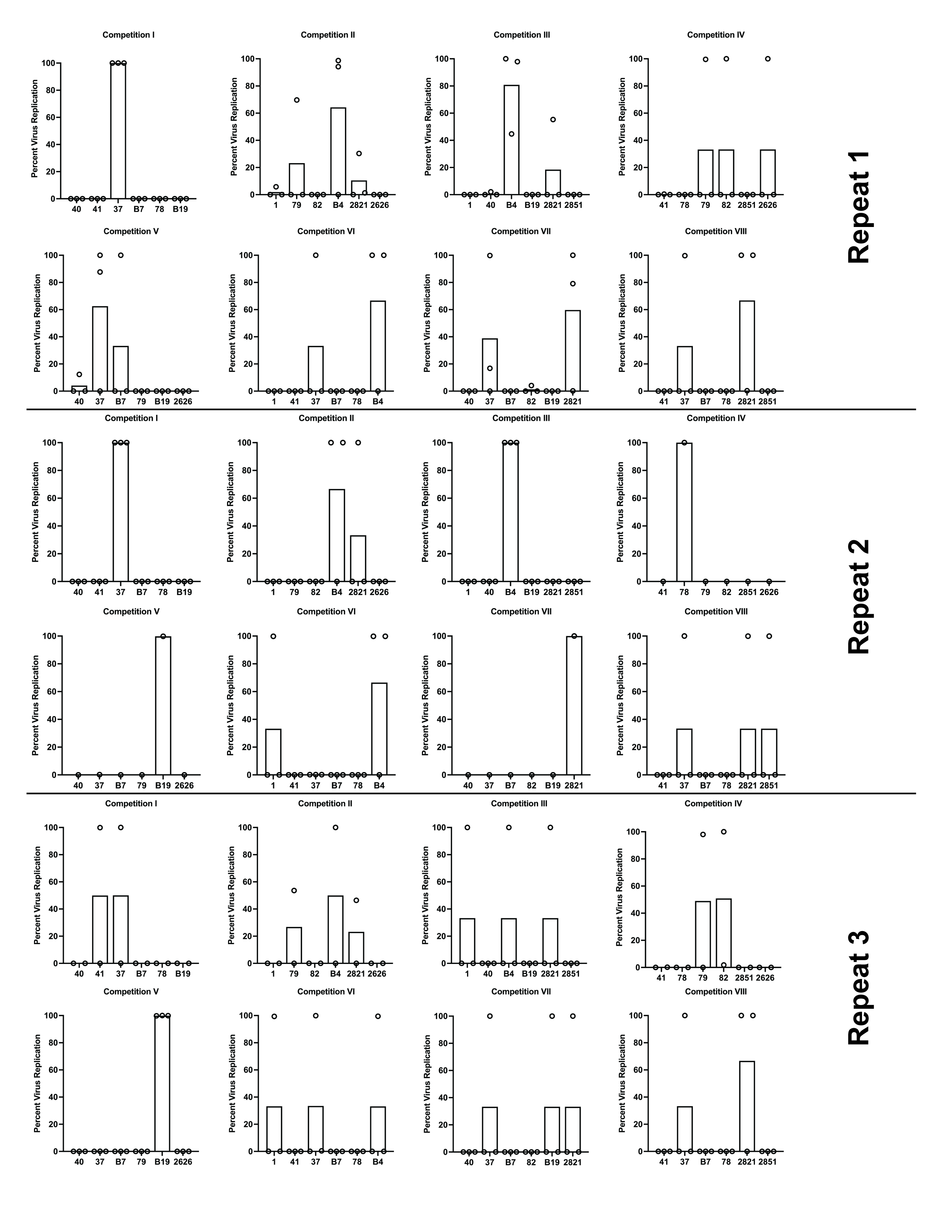

Supplement: S7 Fig — Illumina sequencing results from competitions were processed using SeekDeep and the content of each virus in each competition was calculated. (TIF) [file ppat.1013177.s007.tif]

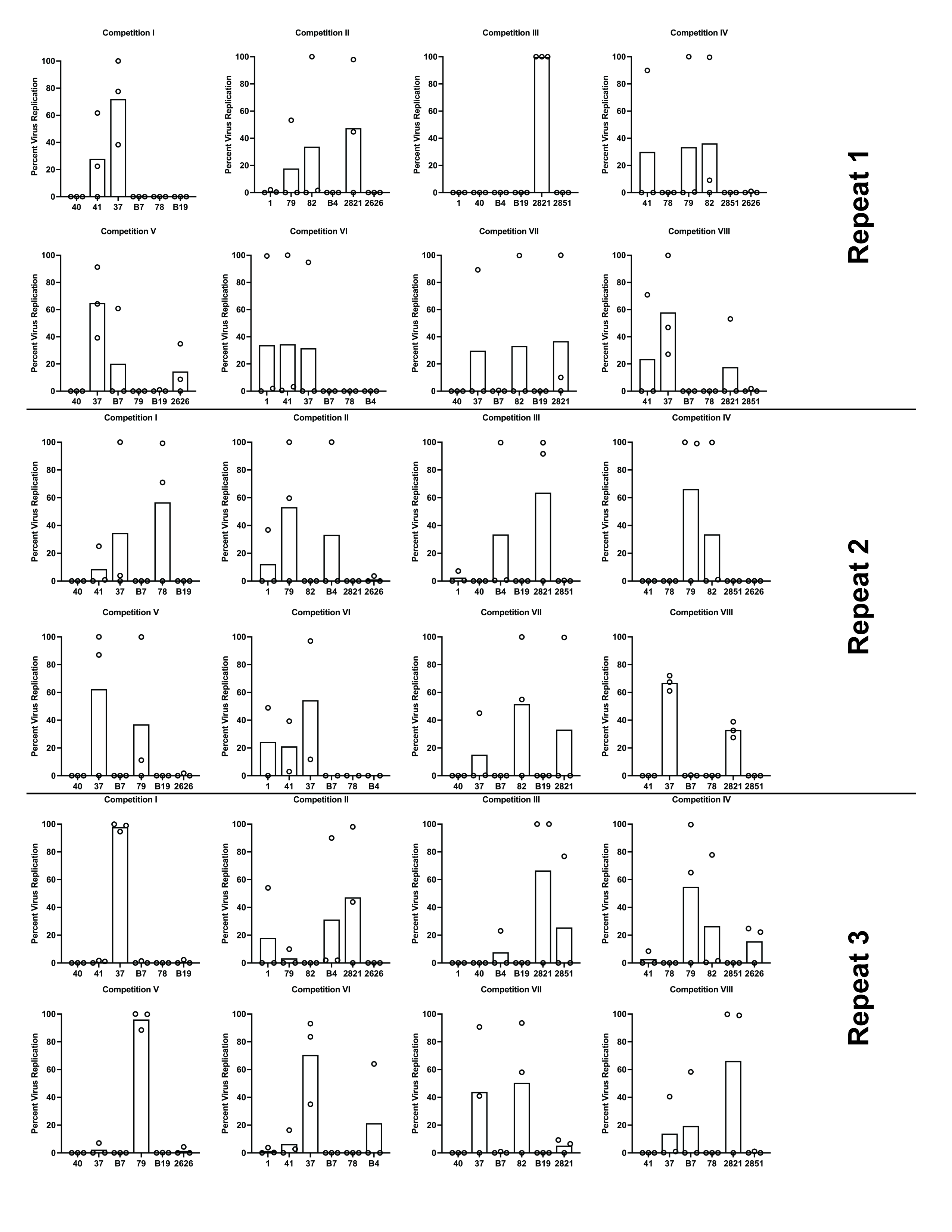

Supplement: S8 Fig — Illumina sequencing results from competitions were processed using SeekDeep and the content of each virus in each competition was calculated. (TIF) [file ppat.1013177.s008.tif]

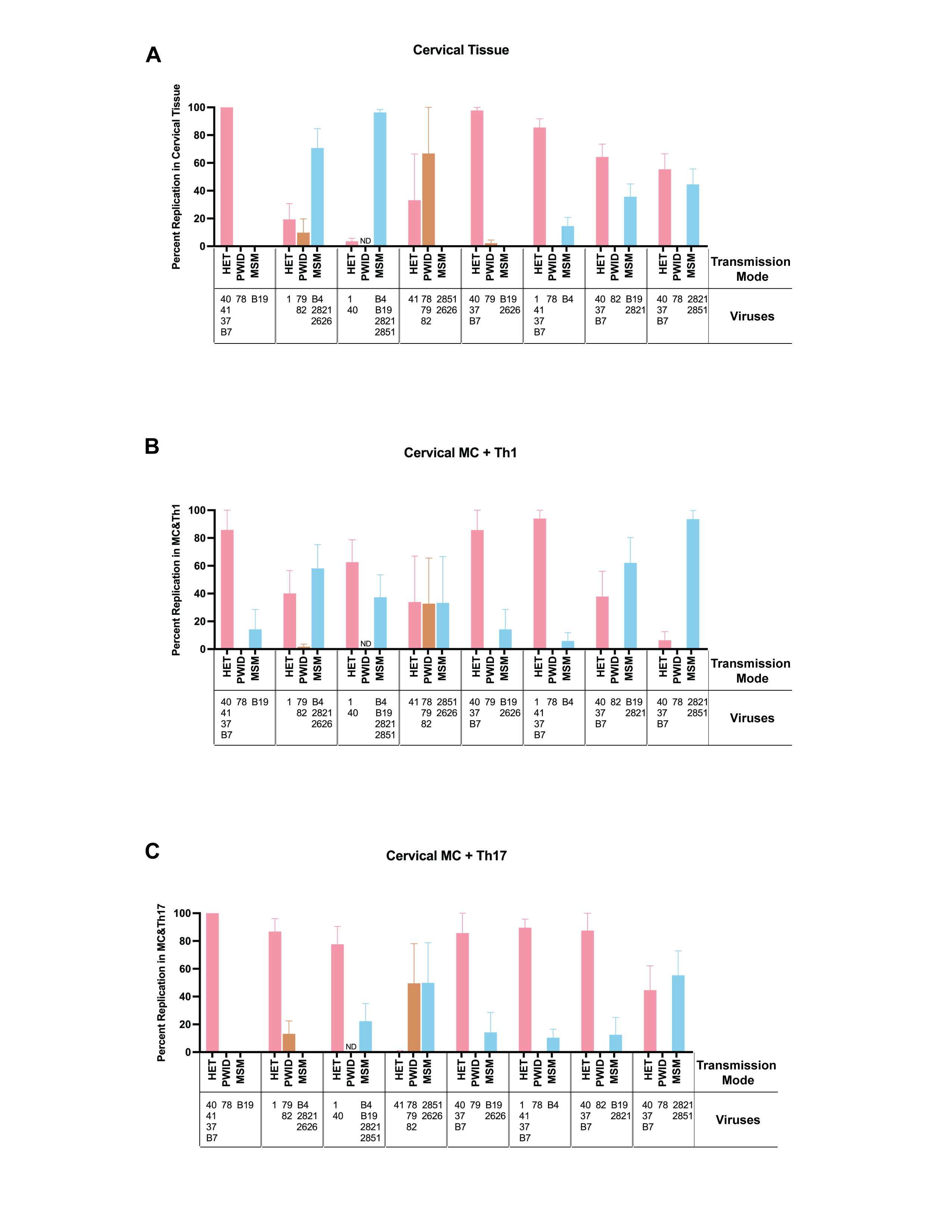

Supplement: S9 Fig — (A-C) The average content of viruses from each risk group was calculated based on SeekDeep results. Viruses that participated in each competition group are indicated at the bottom of the figure. MC + Th1, migratory cells and Th1 co-culture; MC + Th17, migratory cells and Th17 co-culture. (TIF) [file ppat.1013177.s009.tif]

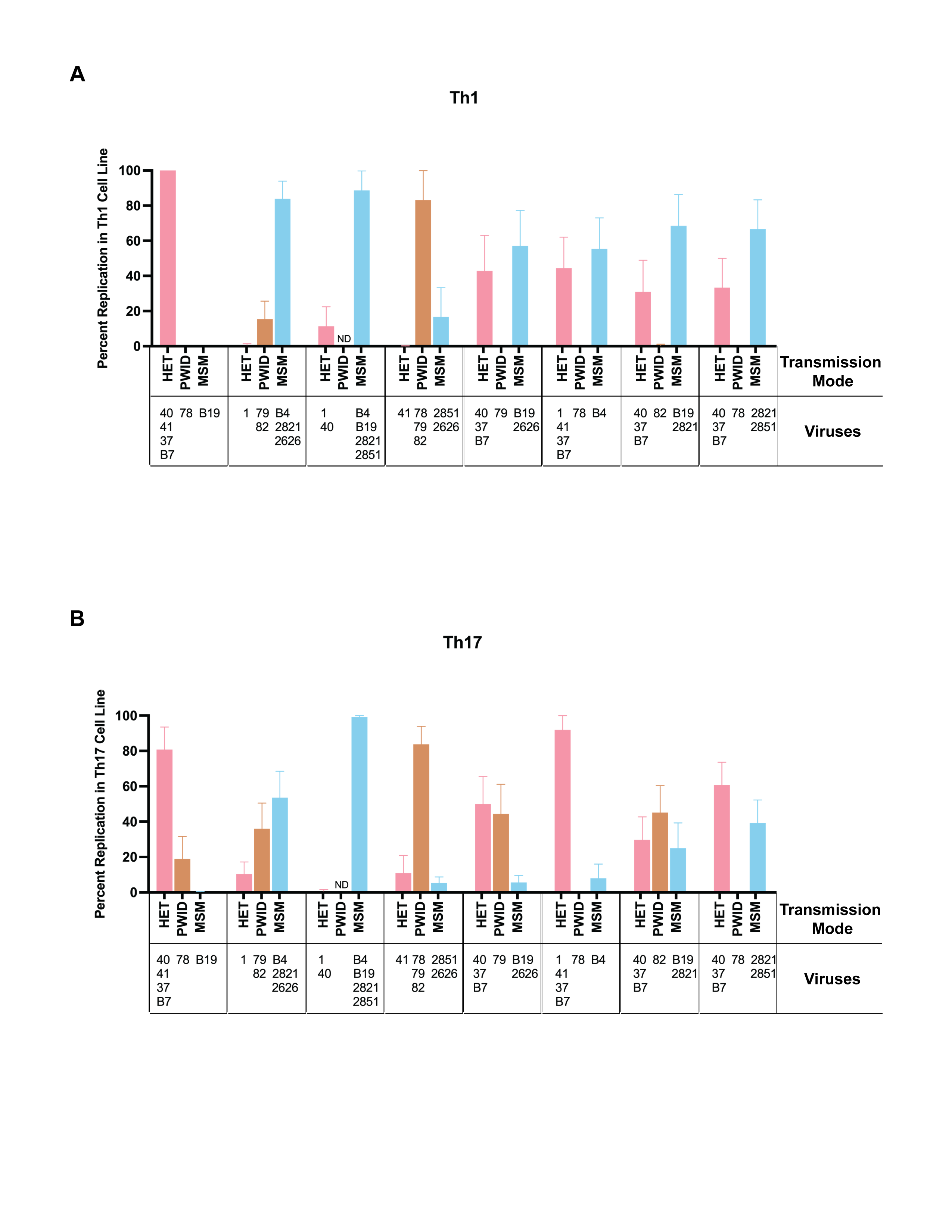

Supplement: S10 Fig — (A-B) The average content of viruses from each transmission group was calculated based on SeekDeep results. Viruses that participated in each competition group are indicated at the bottom of the figure. (TIF) [file ppat.1013177.s010.tif]

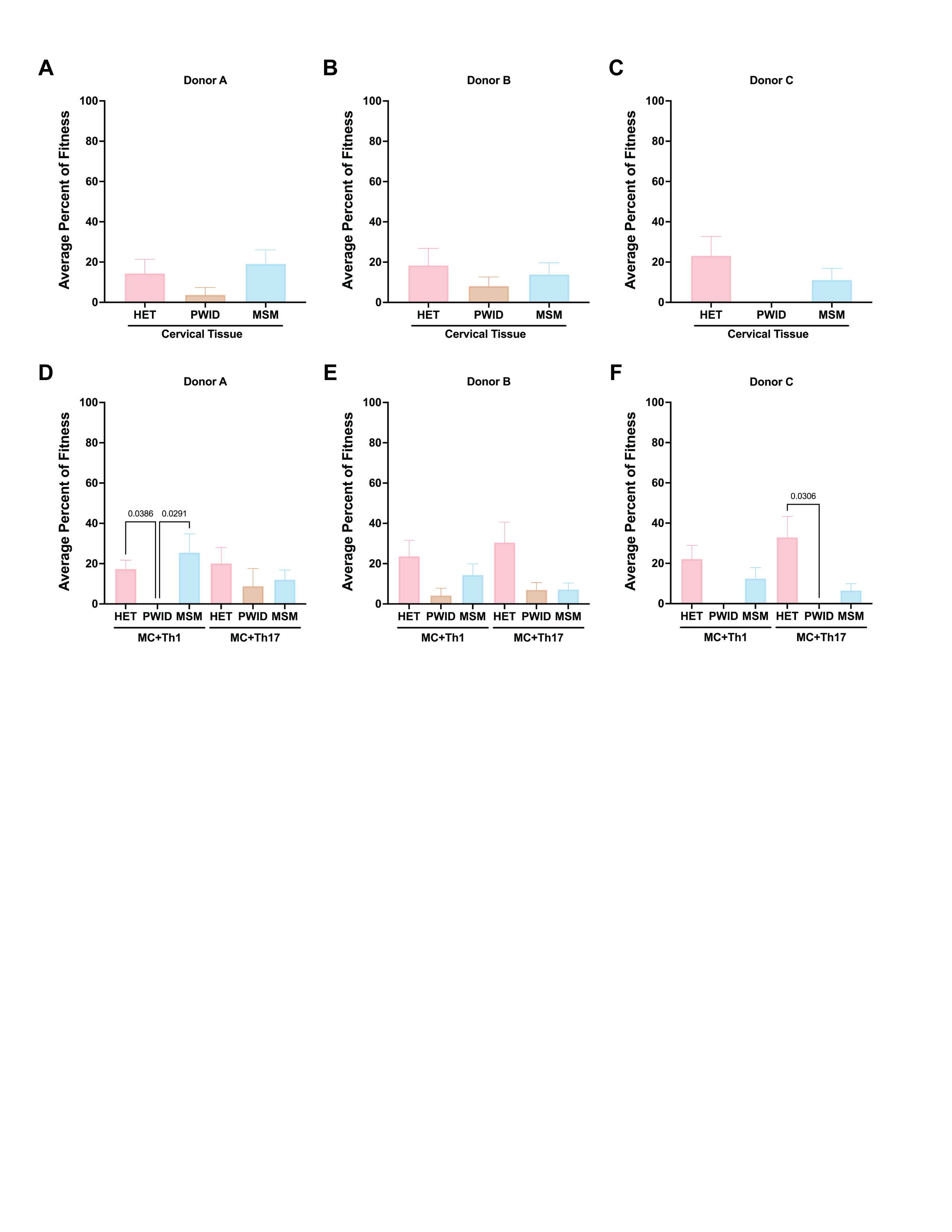

Supplement: S11 Fig — (A-F) Grouped average percent of fitness in cervical tissue, migratory cells and Th1 co-culture (MC + Th1), migratory cells and Th17 co-culture (MC + Th17) from each donor. The Kruskal-Wallis test followed by Dunn’s multiple comparisons test was performed. (TIF) [file ppat.1013177.s011.tif]

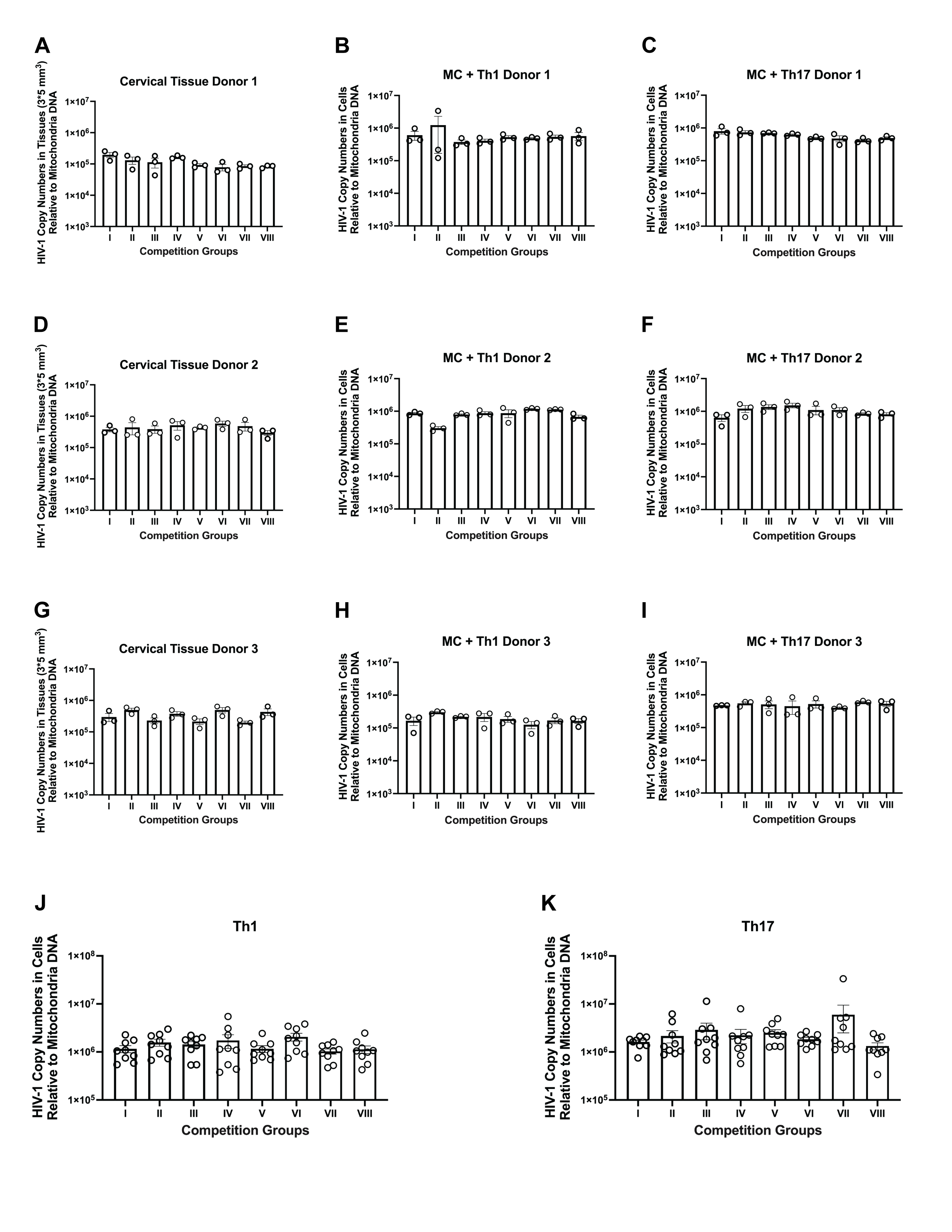

Supplement: S12 Fig — (A-K) qPCR was performed to evaluate HIV-1 relative copy numbers on DNA extracted from cervical tissue (~ 3 pieces of 5 mm3 tissue), migratory cells and Th1 co-culture (MC + Th1), migratory cells and Th17 co-culture (MC + Th17) in each donor and Th1 and Th17. (TIF) [file ppat.1013177.s012.tif]

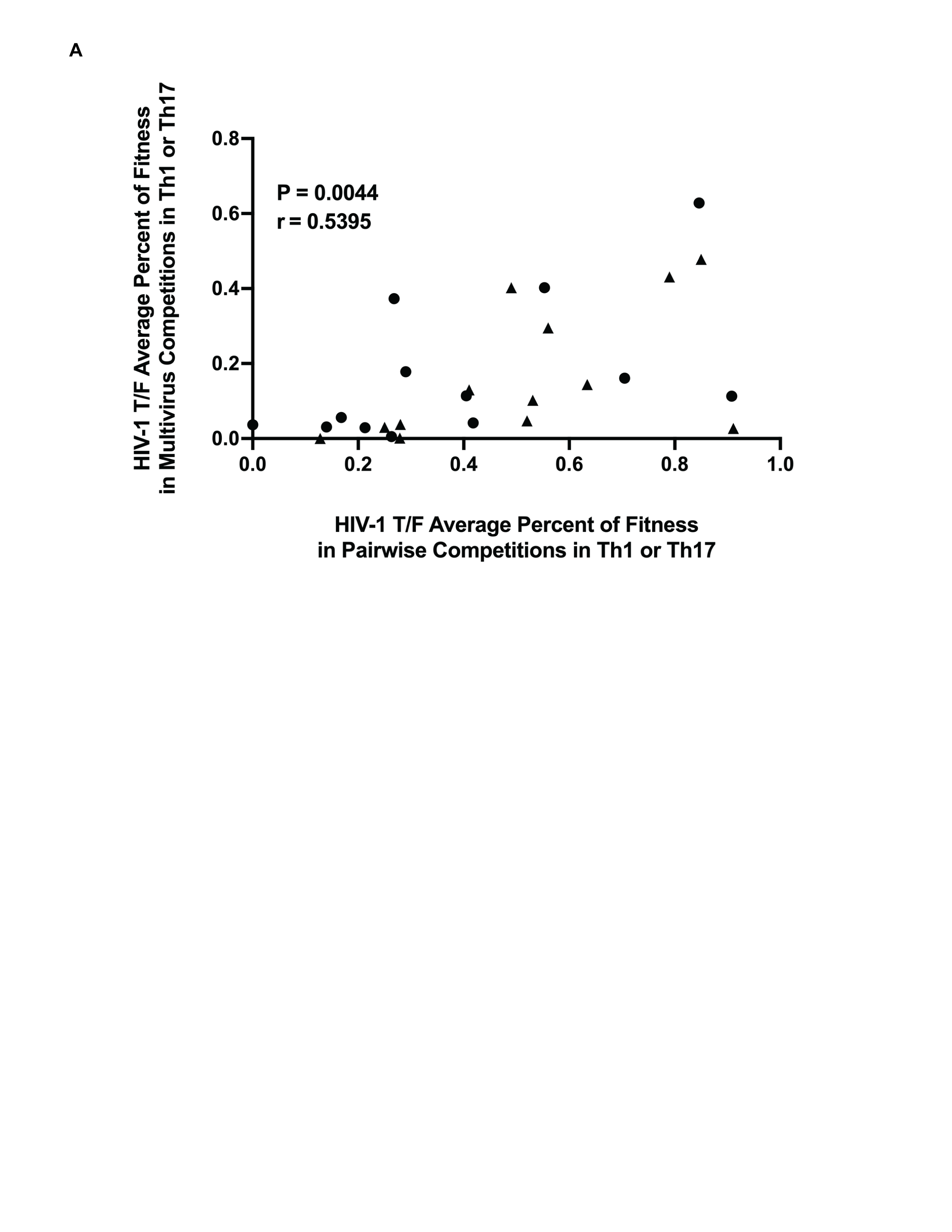

Supplement: S13 Fig — (A) Pearson correlation analysis result between multivirus and pairwise competitions in Th1 or Th17. Dots represent data acquired from Th1 and triangles represent data acquired from Th17. (TIF) [file ppat.1013177.s013.tif]

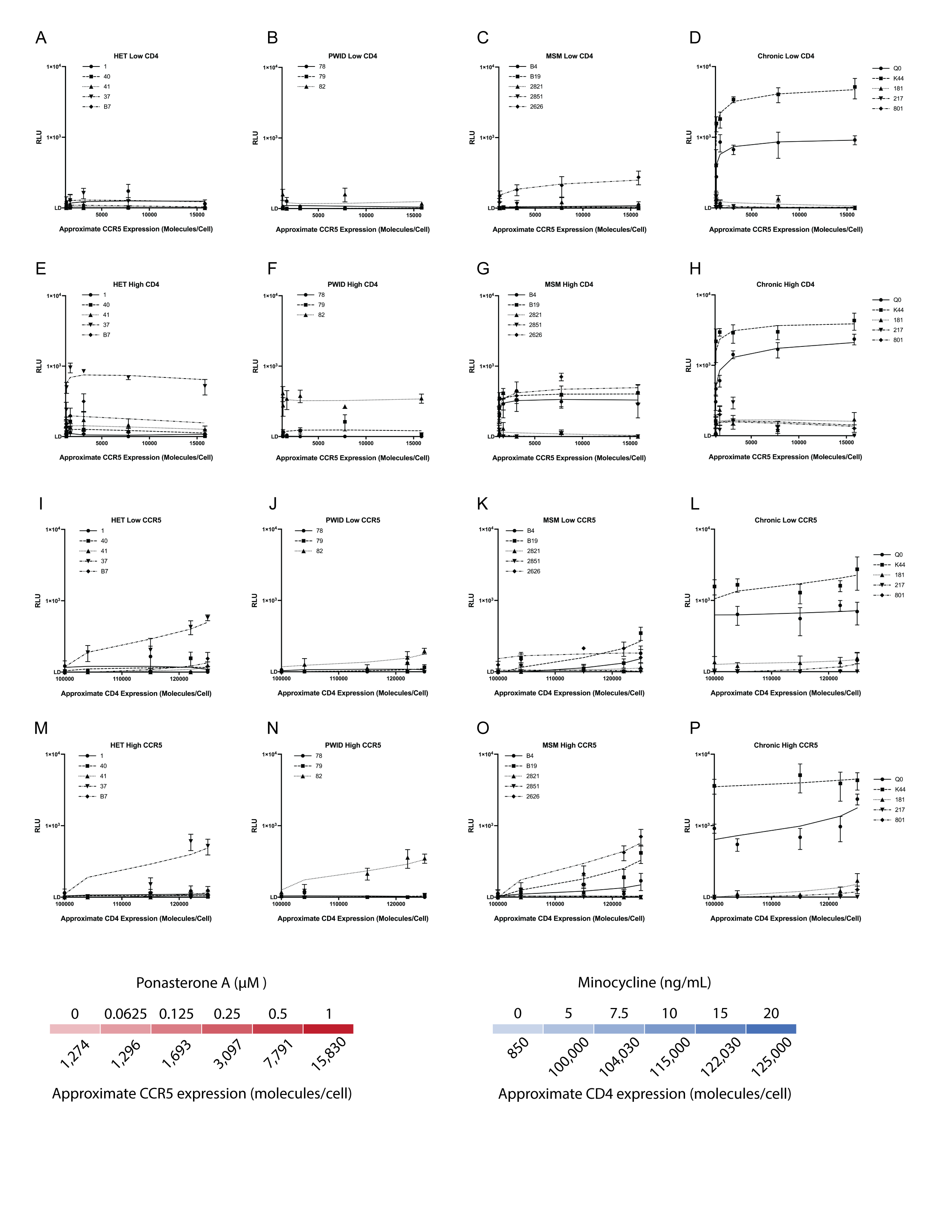

Supplement: S14 Fig — Affinofile cells were induced with a range of concentrations of minocycline (from 0 to 20 ng/mL) and ponasterone A (from 0 to 1 µM) to independently and simultaneously express varying levels of CD4 (approximately 850–125,000 molecules per cell) and CCR5 (approximately 1,274–15,830 molecules per cell). Subsequently, these cells were infected with T/F viruses from different transmission modes or chronic viruses. Virus infectivity was assessed by measuring relative light units (RLU) from luciferase activity and plotted against the approximate CD4 (A-H) or CCR5 (I-P) expression levels, considering both low (approximately 100,000 molecules per cell for CD4; approximately 1,296 molecules per cell for CCR5) and high (approximately 125,000 molecules per cell for CD4; approximately 15,830 molecules per cell for CCR5) expression conditions. LD, limit of detection. (TIF) [file ppat.1013177.s014.tif]

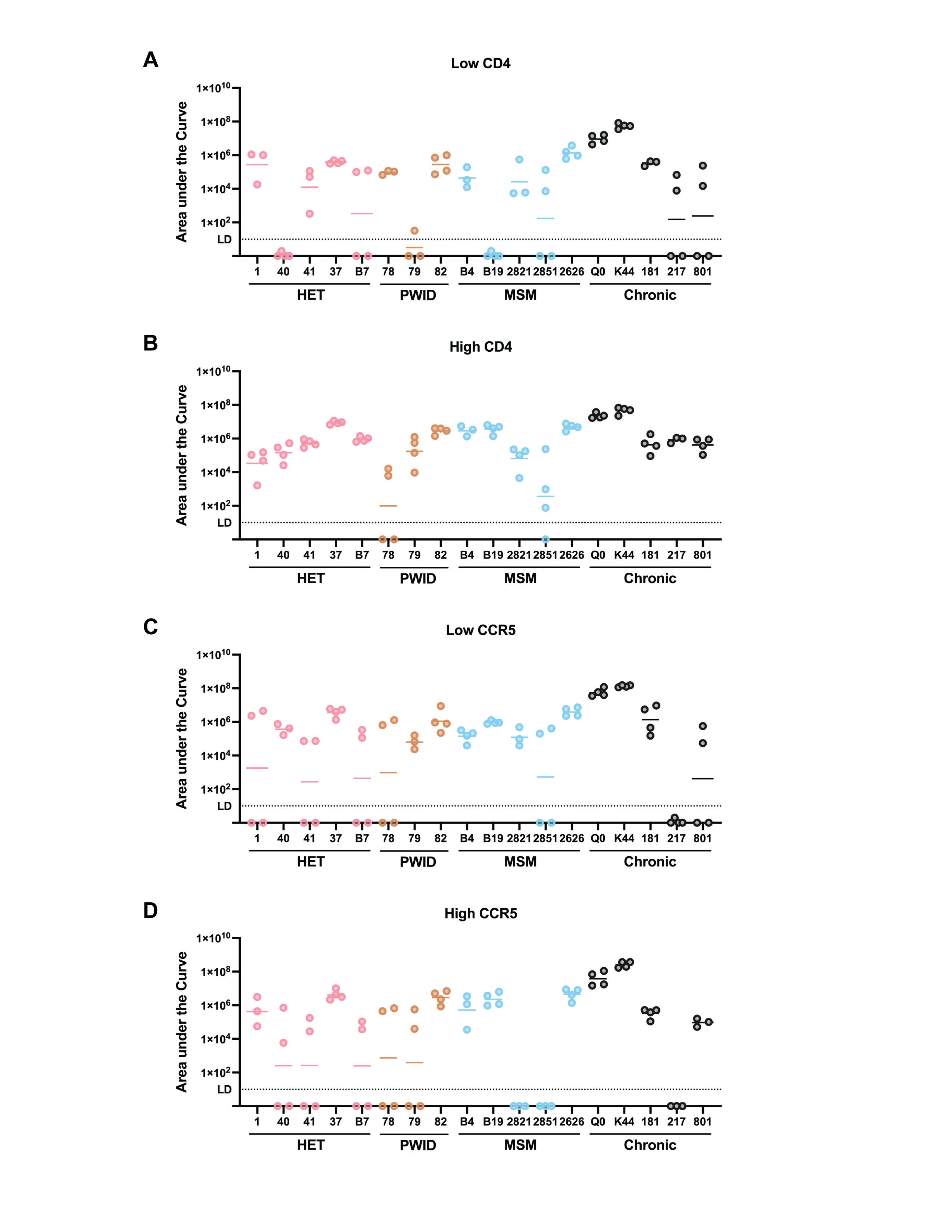

Supplement: S15 Fig — Affinofile cells were induced with a range of concentrations of minocycline (from 0 to 20 ng/mL) and ponasterone A (from 0 to 1 µM) to independently and simultaneously express varying levels of CD4 (approximately 850–125,000 molecules per cell) and CCR5 (approximately 1,274–15,830 molecules per cell). Subsequently, these cells were infected with T/F or chronic HIV-1. (A-D) The area under the curve was calculated for each virus under low and high CD4 and CCR5 expression levels based on response curves in S14 Fig. LD, limit of detection. (TIF) [file ppat.1013177.s015.tif]

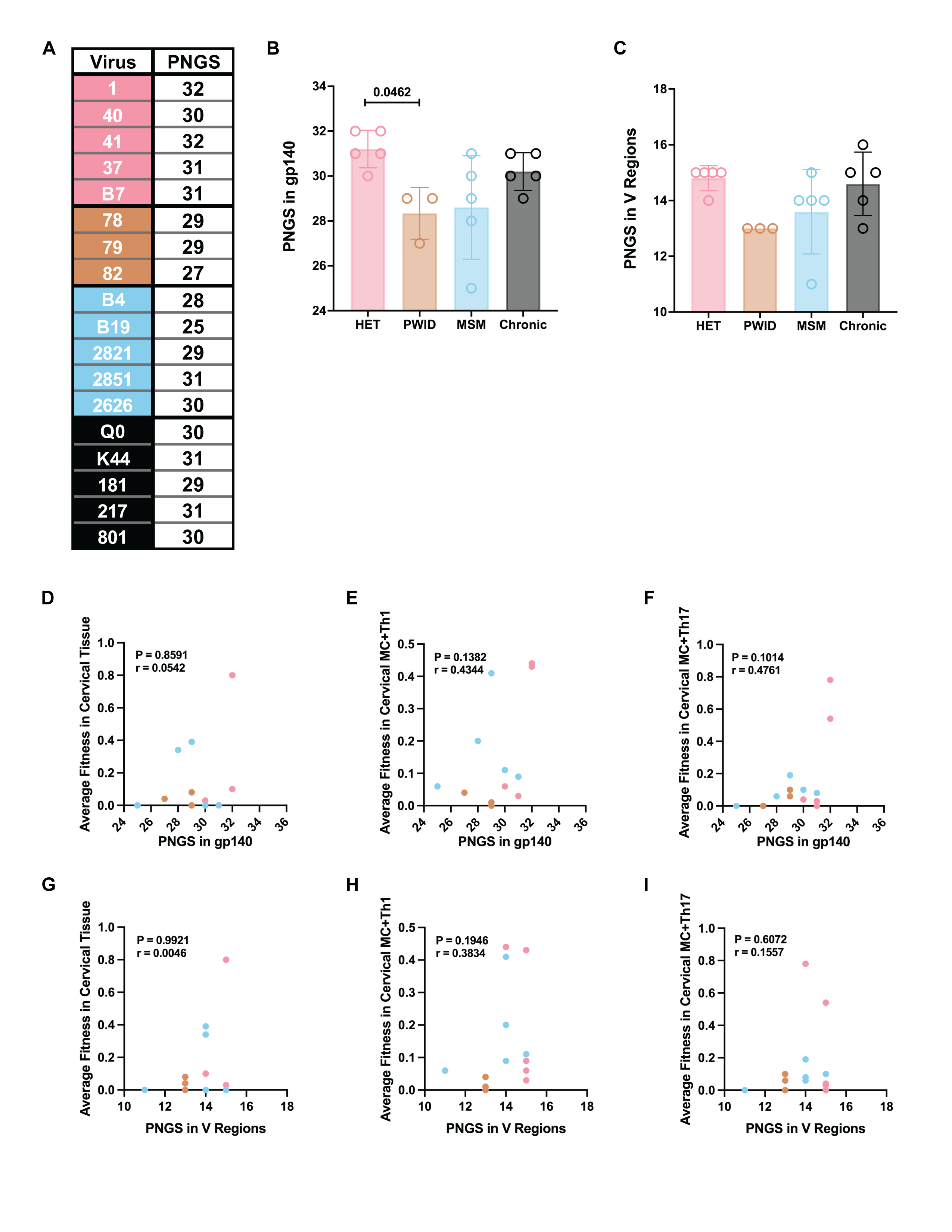

Supplement: S16 Fig — (A) The PNGS on HIV-1 gp140 region. (B) Kruskal-Wallis analysis on PNGS in gp140 based on different transmission modes. (C) Kruskal-Wallis analysis on PNGS in Env variable regions based on different transmission modes. (D-F) Spearman correlation analysis between PNGS in gp140 and average percent of fitness during competitions. (G-I) Spearman correlation analysis between PNGS in Env variable regions and average percent of fitness during competitions. PNGS was predicted using the Los Alamos database. (TIF) [file ppat.1013177.s016.tif]

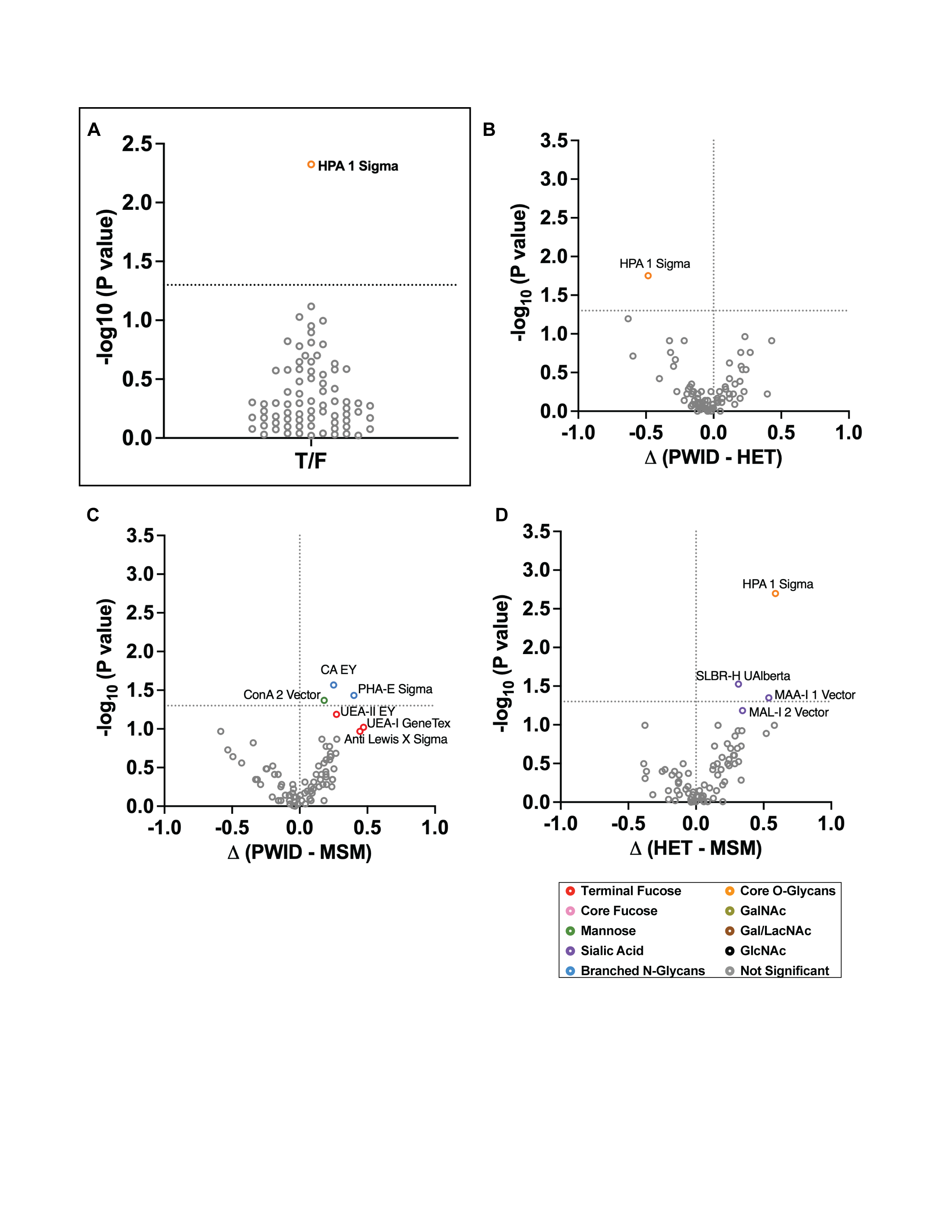

Supplement: S17 Fig — Kruskal-Wallis (A) and Mann-Whitney (B-D) tests were performed on the log₂(S/R) values from the lectin microarray data. The x-axis in panels B-D represents the log₂(S/R) difference between the two transmission groups. The y-axis in panels A-D represents the -log₁₀(P value) from the Kruskal-Wallis or Mann-Whitney tests. (TIF) [file ppat.1013177.s017.tif]

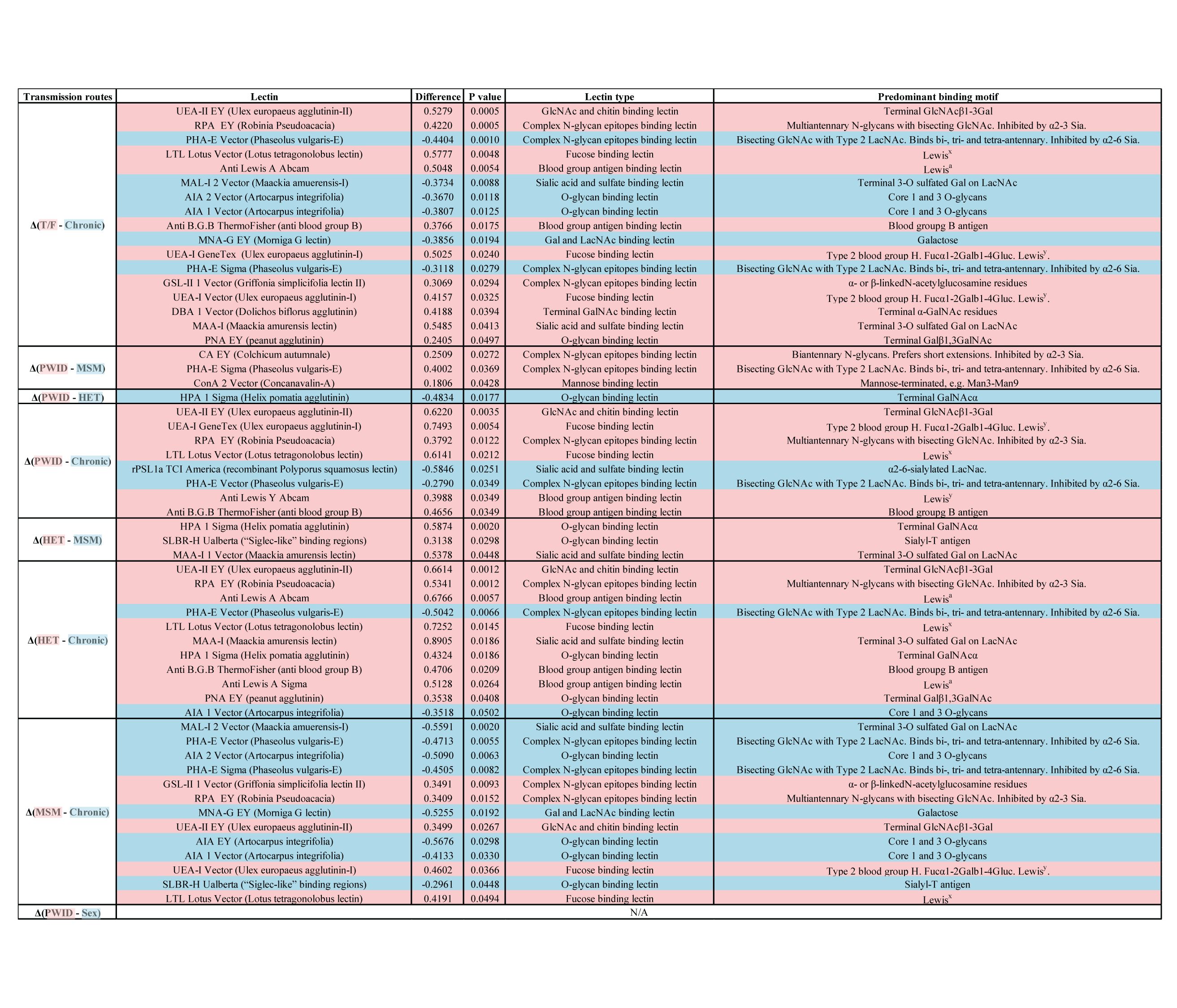

Supplement: S18 Fig — Mann-Whitney tests were performed on the log₂(S/R) values from the lectin microarray data. The table displays all lectins that showed significant differences in the Mann-Whitney test based on the lectin microarray data. The names, types, and predominant binding motifs of the lectins are also provided in the table. The “Difference” column shows the log₂(S/R) difference between the two transmission groups (listed in the left column). The P-values were obtained from the Mann-Whitney test. Lectins highlighted in red indicate significantly higher binding avidity, while those in blue indicate significantly lower binding avidity in the comparison. (TIF) [file ppat.1013177.s018.tif]

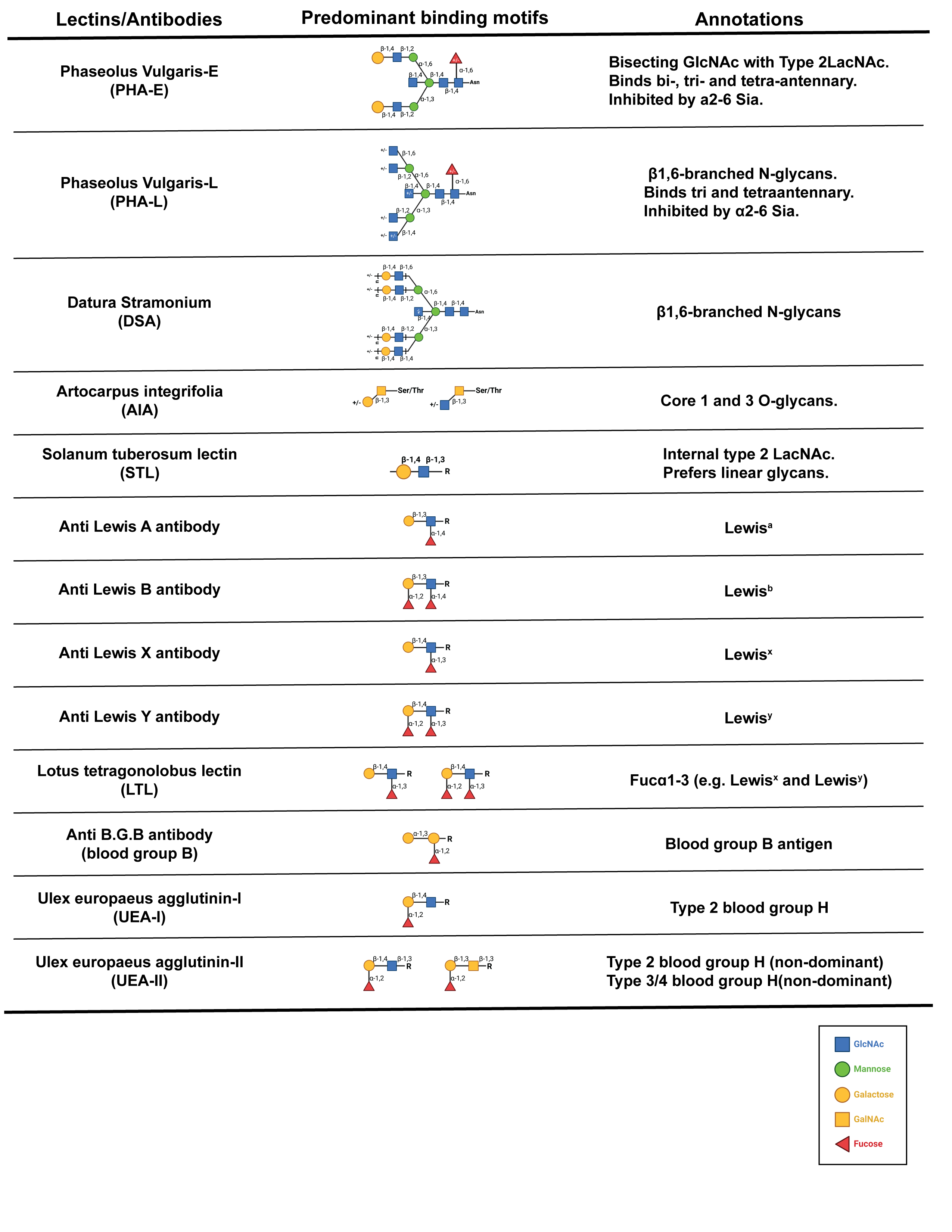

Supplement: S19 Fig — Abbreviations and symbols: Mannose (Man, green circles), N-acetylglucosamine (GlcNAc, blue squares), N-acetyllactosamine (LacNAc, blue square + yellow circle), fucose (Fuc, red triangles), galactose (Gal, yellow circles), N-acetylgalactosamine (GalNAc, yellow squares). R, the rest of the molecule. The figure was created with BioRender.com. (TIF) [file ppat.1013177.s019.tif]

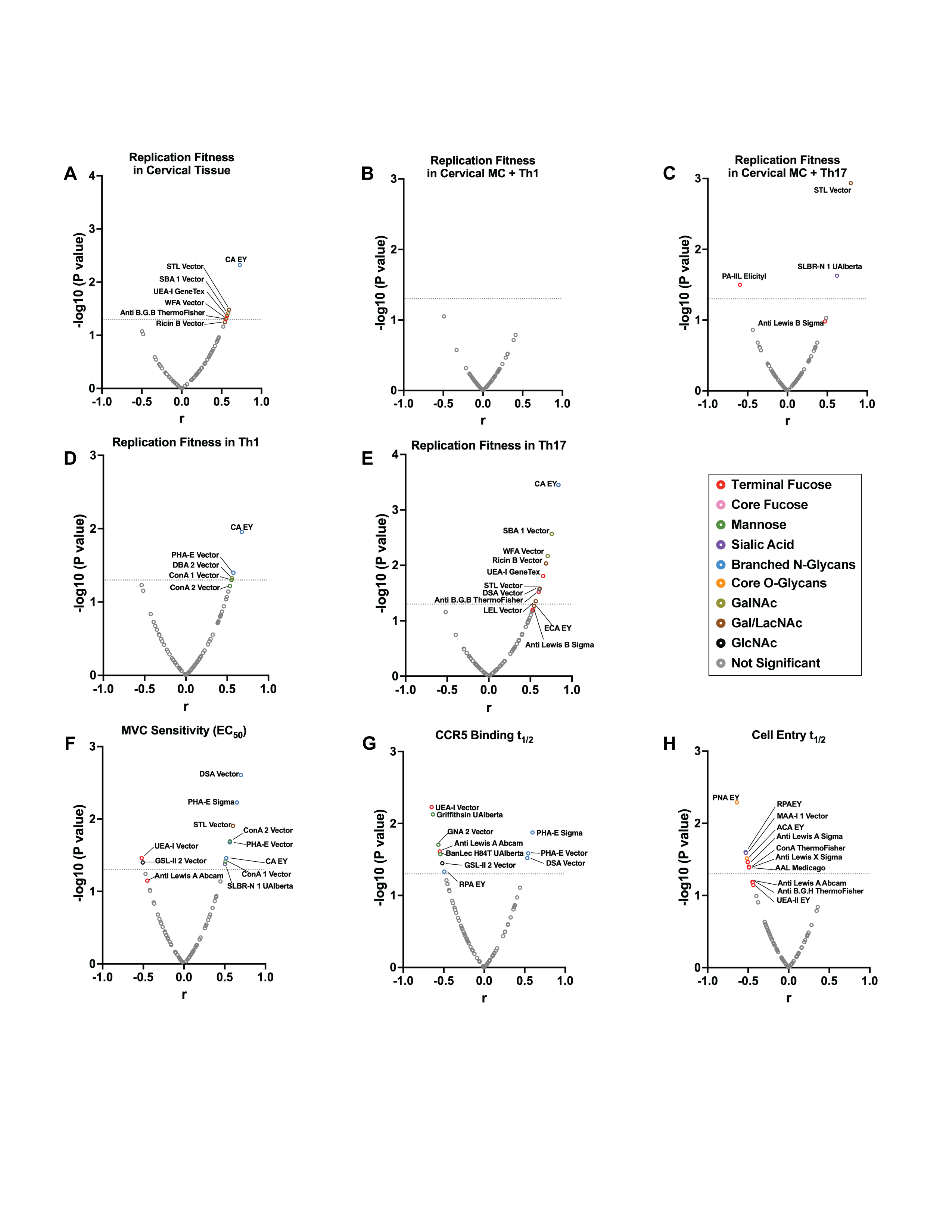

Supplement: S20 Fig — The x-axis represents the r value from correlation analysis, and the y-axis represents the -log₁₀(P value) of correlation analysis. Panels A-E include transmission fitness data from all 13 T/F HIV-1 chimeras, while panels F-H include phenotypic data from 13 T/F and 5 chronic HIV-1 chimeras studied. The dotted line indicates a significance threshold of P = 0.05. The corresponding two-tailed Pearson correlation analysis is given in Fig 8. (TIF) [file ppat.1013177.s020.tif]

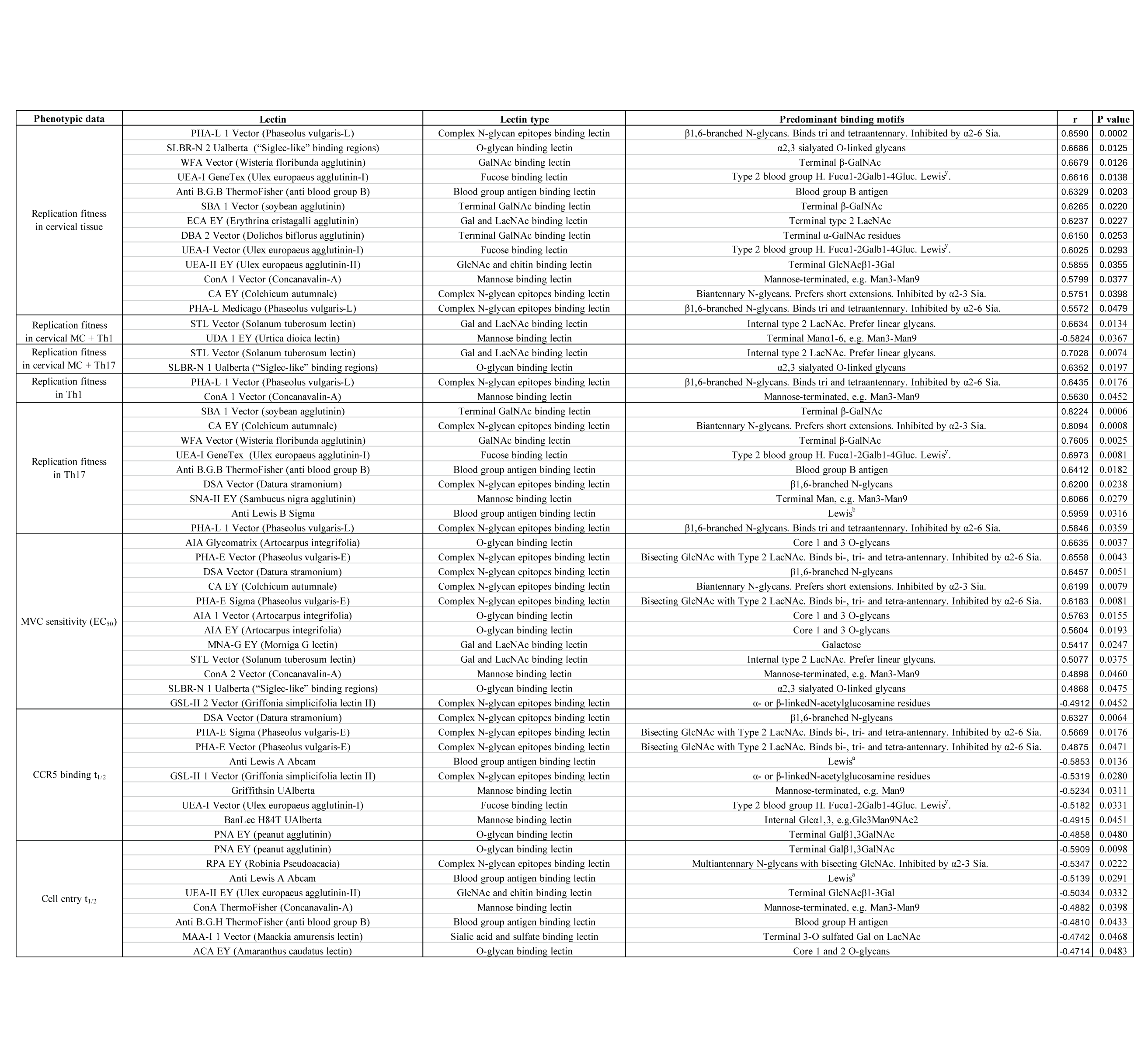

Supplement: S21 Fig — The table displays lectins that reached statistical significance in the correlation analysis. The names, types, and predominant binding motifs of the lectins are provided, along with the r and P-values from the correlation tests. The types of phenotypic data tested in this study are listed in the left column. The corresponding data visualization is presented in Fig 8. (TIF) [file ppat.1013177.s021.tif]

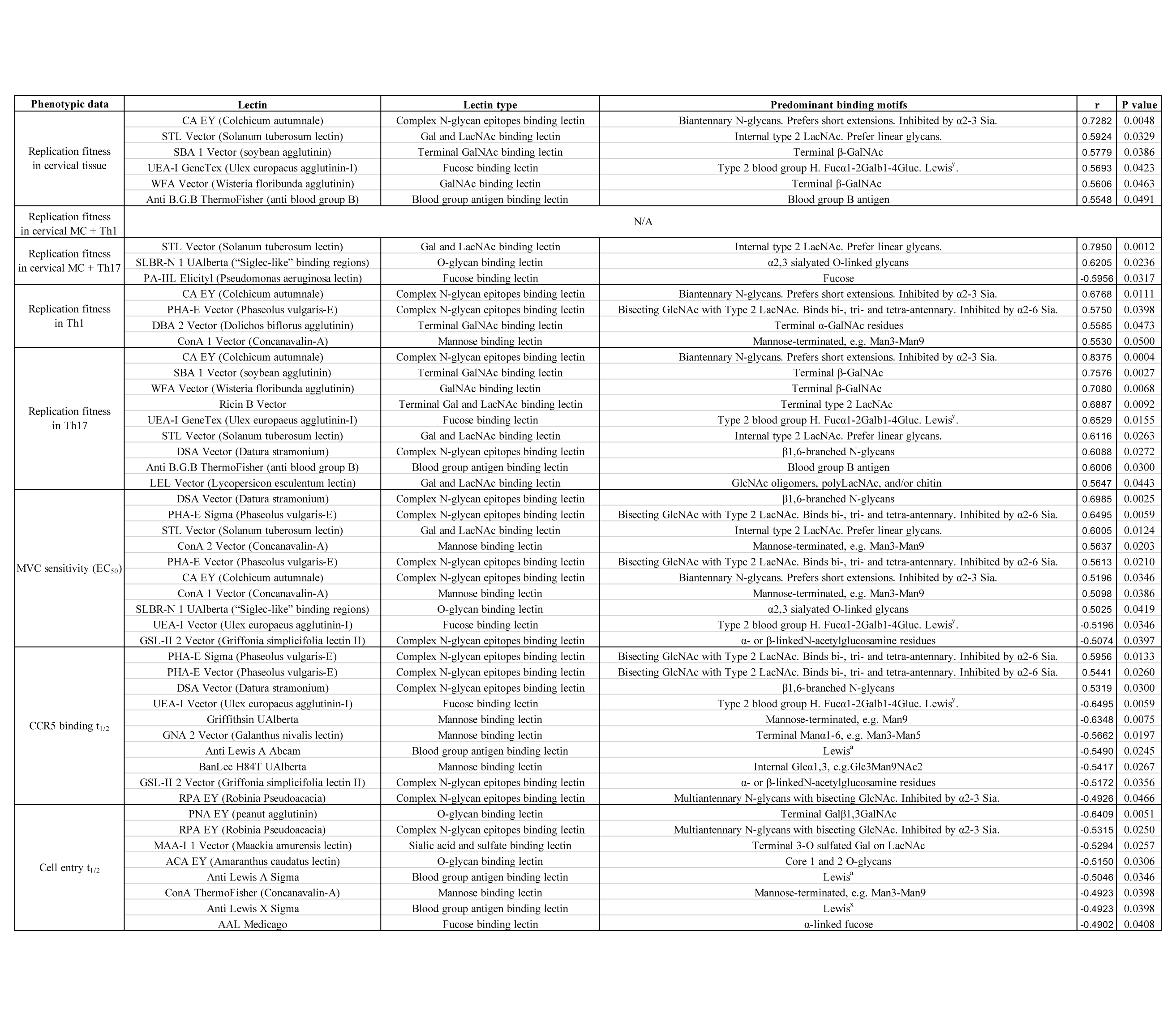

Supplement: S22 Fig — The table displays lectins that reached statistical significance in the correlation analysis. The names, types, and predominant binding motifs of the lectins are provided, along with the r and P-values from the correlation tests. The types of phenotypic data tested in this study are listed in the left column. The corresponding data visualization is presented in S20 Fig. (TIF) [file ppat.1013177.s022.tif]

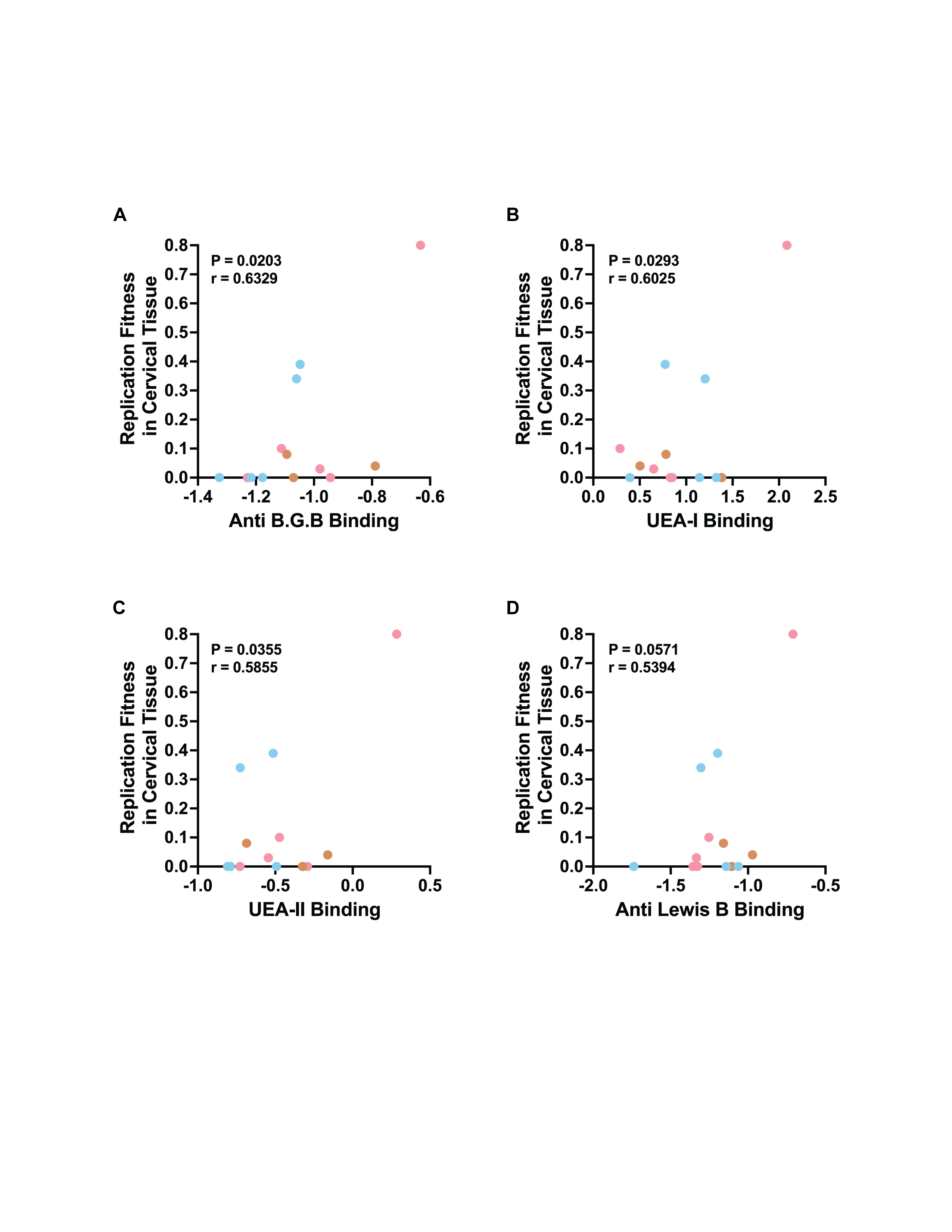

Supplement: S23 Fig — The two-tailed Pearson correlation analysis was performed. (TIF) [file ppat.1013177.s023.tif]
