# Supplementary material for: Transmitted/founder (T/F) HIV-1 derived from sexual contact exhibits greater transmission fitness in human cervical tissue than T/F HIV-1 from blood-to-blood contact: Unique glycan profiles on T/F envelopes associated with transmission phenotypes
Source: PLoS Pathog. 2025 May 23;21(5):e1013177. doi: 10.1371/journal.ppat.1013177 (PMC12140434; doi:10.1371/journal.ppat.1013177)
Supplement: S2 Fig — (PDF) [file ppat.1013177.s002.pdf]

```
                         10        20        30        40        50        60        70        80        90        100
TF HIV-1 Env        ....|....|....|....|....|....|....|....|....|....|....|....|....|....|....|....|....|....|....|....|....|....|
1                   GTGCAGAACAGGCGTGGGTCACAGTCTATTATGGGGTACCTGTGTGGAAAGAAGCAAATACCACTCTATTTTGTGCATCAGATGCTAAAGCATATGATCC
37                  C.A...G..GATT......T......................................C....................................TC.A.
40                  C.A.....A.ATT..........A.................................CC.......................................A.
41                  C.A...GTA.ATT......T.....................................CC.........................................
B7                  C.....G.A.TTT..........................................G.CC.......................................A.
78                  C.....G...ATT............................................CC.......................................A.
79                  C.A...GTA.TTT............................................CC.............................G.........A.
82                  C..T......ACT.....................A......................CC.......................................A.
B4                  C.......A.CTT............................................CC.......................................A.
B19                 C......CA.CTT...................................G........CC..T....................................A.
K44                 CAA.......ATT............T........A..G...................CC......................................G..
Q0                  C.......A..AT............T.................A..............C......T.............................AG.A.
801                 C.......A..TT............................................CC..T....................................A.
217                 C.........ATT........................G...................CC.......................................A.
181                 C.A...---.ATT......T................................G....CC......................................CA.
2851                C.A.....A.ATT............T...............................CC......T.........................T.....GA.
2626                C.A....CA.ATT............T..............................GTC........G..............................A.
2821                C.A.....A.TTT.....................A......................CC.......................................A.
Clustal Consensus       **       ****** *** * ******** ** ***** **** *** **    ** *** * ******************** ** ***    *

                        110       120       130       140       150       160       170       180       190       200
                    ....|....|....|....|....|....|....|....|....|....|....|....|....|....|....|....|....|....|....|....|
1                   AGAGGCACATAATGTTTGGGCCACGCATGCCTGTGTACCTACAGACCCCAACCCACAAGAATTACCAGTGCTAAATGTGACAGAAAATTTTAACATGTGG
37                  .....T...............A..A..............C.....................A..AA.T..GA............................
40                  .....T..................A..............C.....................A..GA.T..GG............................
41                  .....T..................A...........C..C.....................G..AA.T..GA...............G......GCA...
B7                  .....T.........C........A..............C.....................G..GT.T..GG............................
78                  .....T..................A..C...........C.........GG..........G..A..T..GA......A..............T......
79                  .....TG.................A..............C..........G..........G.....T..GA............................
82                  .....T..................A..............C.....T...............G..AACT..AA....A.A.....................
B4                  ...A.T..................A..............C.....................G..GT.T..GA............................
B19                 ........................A..C...........C.....................G..GA.T.AAA.....................T......
K44                 .....T..................A..............C.....................G..GT.T..GG.....................T......
Q0                  .....T..................A...........G..C..........G..........G..GT.T..GG.............G.A............
801                 .....T..................A..............C.....................A.GGA.T..GC............................
217                 .....T..................T..............C.....................G..GT.T..GA......A...........C.........
181                 ........................A..............C..........G..........G..AT.T..GA.............G..............
2851                .....T..................A..............C........................GT.T..GA......A......T.....G........
2626                ...A.T..................A..............C.........G...........G..GT.C..GA............................
2821                ...A.T......C...........A..............C.....................G..GT.T..GA...............C............
Clustal Consensus   *** *  ***** ** ***** ** ** ******** ** ***** ***  ********** *     *   **** * ****** * **  *    ***

                        210       220       230       240       250       260       270       280       290       300
                    ....|....|....|....|....|....|....|....|....|....|....|....|....|....|....|....|....|....|....|....|
1                   AAAAATAACATGGTAGAACAAATGCATGAGGATATAATCAGTTTATGGGATCAAAGCCTAAAGCCATGTGTAAAATTAACCCCACTCTGTGTTACTTTAA
37                  C...................G...A................C....................A.......................T.....C.......
40                  .........................................C.........................................T................
41                  ..T....G............G.................T..................T............................T..C..........
B7                  ....................G...............................................................................
78                  ....................G..A.................C...............T................GC...................C....
79                  ....................G....................C.........G.....T................GC...................C....
82                  G...................G...................A.............TT............................G.......GG......
B4                  ....................G........A........T.......................A.....................................
B19                 G...................G....................................T....A.................T...................
K44                 ......G.....................................................................................C......G
Q0                  ..............G.............................G.........................................G........C...C
801                 ....................G..................GAGC.................................................C.......
217                 ....................G...............................................................................
181                 .................T..G....................C..........................................................
2851                ....................G........A........T.......................A............C................C.......
2626                .....C..............G................T..............................................................
2821                ....................G.......................................C.A..........G......T...................
Clustal Consensus    * **   ****** ** ** **  **** ********     * ****** **  * ** * **********   **** **  * ** **  * ***

                        310       320       330       340       350       360       370       380       390       400
                    ....|....|....|....|....|....|....|....|....|....|....|....|....|....|....|....|....|....|....|....|
1                   ATTGCACTGATGTGGAAACCAAAAGGACTAATGCCACTGATACTAATGCCACTACTACTAGGACCACTAGTACTCCTAGTACAAACA---GTAGTGGAGT
37                  ..........CT------------------------------------TG.GA.A.GAA..TGAT....A.C.CA...T..GTCC------------C.G
40                  .G....A....ACC..GGTT.CT.AT------------------------..........AT..------....A...A..GC.C---------------
41                  ..........-TAT.GTGA-----------------------------.T.A...C..AG.A.AT.GC...G...T..A..TG.C---------------
B7                  ....T.....---T.TG.ATGGT.------------------------.T.G-------------G...A.GTCA.....--------------------
78                  ....T.....GT------------------------------------TA.G..A.G...ATTAT..C.C..A.AG.....GGG.------------..A
79                  ....T..A..GT------------------------------------TA.G..------------.C.C..A.AG...---------------------
82                  ...........T------------------------------------TG.A.GT.GA.TT..ATGT..A..A.A.C.T..AT.G------------TAG
B4                  ........A.---T.TG.ATGTG.------------------------.T.A.TTG.AG.ATGAG....A...CAA.....GT.G---------.....G
B19                 ...........T.A.GG.AT------------------------------GT.....A..CC..T.A....------------------------AAC.G
K44                 ...........T..A.T..T.CT.TTT.A.------------------AG.A.G......ATT.A.GG.A...A...A...C.CT.---......A.TTG
Q0                  ........A..---------------------------------------GT...A..A.ATG......A...CA....GAT.GTT--------------
801                 ...........T.AA.G.AT.CT.CT.A.T------------------.G.A........AT..........G.AGGG.GGA..CG.---TGGAGAA..G
217                 ...........T...GG.ATGCC.------------------------.T.....C.A..ATG......A..TCAA.....GT.GT.---TC.A.....G
181                 ..........CTAT.GG.ATG.T.CT.A..C.AA.T...GG.A.G..A.T.G...C.AGG.T.AT.AC.CC...AG.G...AC.C..CTA.....AACTG
2851                ...........T------------....G...G........TGG..A.....A..G..A-----------------..A...C.CT.---T...AA...A
2626                ...........TATA.C.AT.---------------------------.TG.....A...CC....G....G..A...C...CGC..GTA..GC.AATAA
2821                ...........T---------------------------------------A.GTC.AG.ATGTT....A.G.CA.......T..------------T.C
Clustal Consensus   * ** *   *
```

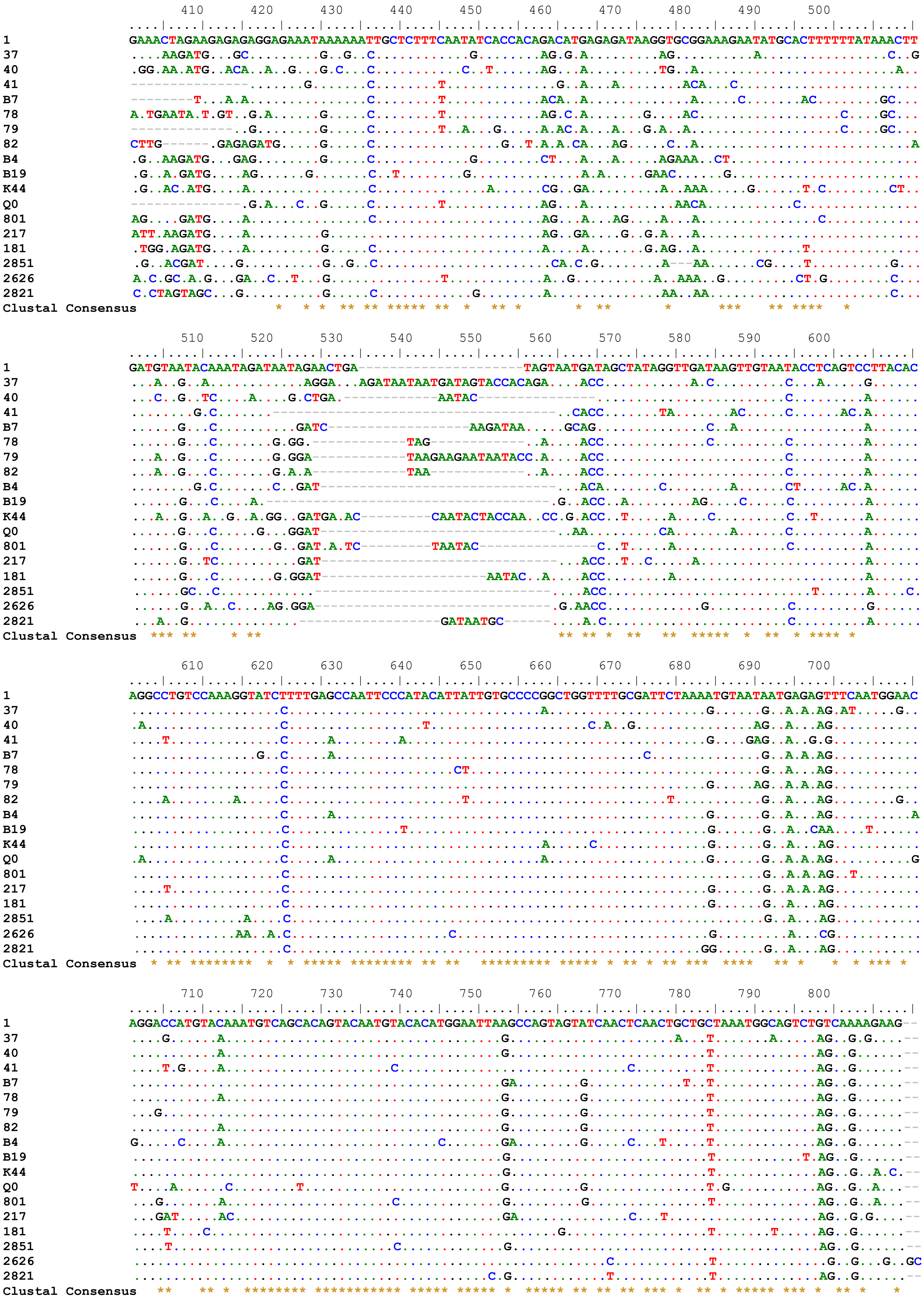
410 420 430 440 450 460 470 480 490 500
1 GAAACTAGAAGAGAGAGGAGAAATAAAAAATTGCTCTTTCAATATCACCACAGACATGAGAGATAAGGTGCGGAAAGAATATGCACTTTTTTATAAACTT
510 520 530 540 550 560 570 580 590 600
1 GATGTAATACAAATAGATAATAGAACTGA-------------------------TAGTAATGATAGCTATAGGTTGATAAGTTGTAATACCTCAGTCCTTACAC
610 620 630 640 650 660 670 680 690 700
1 AGGCCTGTCCAAAGGTATCTTTTGAGCCAATTCCCATACATTATTGTGCCCCGGCTGGTTTTGCGATTCTAAAATGTAATAATGAGAGTTTCAATGGAAC
710 720 730 740 750 760 770 780 790 800
1 AGGACCATGTACAAATGTCAGCACAGTACAATGTACACATGGAATTAAGCCAGTAGTATCAACTCAACTGCTGCTAAATGGCAGTCTGTCAAAAGAAG--
37
40
41
B7
78
79
82
B4
B19
K44
Q0
801
217
181
2851
2626
2821
Clustal Consensus

```
                         810       820       830       840       850       860       870       880       890       900
                  ....|....|....|....|....|....|....|....|....|....|....|....|....|....|....|....|....|....|....|....|
1                 -ATGTAATAATTAGATCCAAGAATTTCTCGGAAAATACTGACATCATAATAGTACAGCTGAATACATCGGTAACAATTAGTTGTATAAGACCCAACAACA
37                -.G...G..........TGCC...........C...GACC.G.A..................AGA...T...GA.....C.....C..............
40                -.G...G..C.......TGCC.........A.C...G..A.A.C............C.....C.A...T...GA.....A.....CG...AT........
41                -.....G..G.C.....TGCC......A..A.C...G..A.A.C..................AGATC.T...GT.....A.....C...G..TTT.....
B7                -.G...G..........TG.A......A.A..C...G..A.A.C....................AC..T...GT.....A.....C............T.
78                -.G...G..........TG.A......A.AA.C...G..A.A.G.............T.....GA...T...GT.....A.....C..............
79                -.G...G..........TG.A......A..A.C...G..A.A.C.............T.....GA...T...GT.....A.....C..............
82                -.G...G..........TG.A......A....C...GT.A.A.C.............T.....GA...T...GA.....AG....C..............
B4                -.CA..G..........TG.A......A....C...G..A.A.A...................GT...CA..GA.....A.....C..............
B19               -.G...G..........AGTC......AGT..C...G..A.A.CA...........A.......A...T...GA.....C...C.C..............
K44               -.GAC.G..........TGCC......A.A..C...G..A.A.CT..................GA.C.T....AC....C...C..G.............
Q0                -.G..............TGCC...........C...GG.A.A.C.........G..T..C....A...T...GA.....A.........G..........
801               -.G...G.......G..TG........A.A..C...G..A.A.C....................A...T...GA....GAG...G............G..
217               -.G........C.....TGTT........A..C...G..A.A.C...................GA...T...GA.....A.....C.........T....
181               -.GA..G....C.....TG.A...C..A..A.C...G..A.A.C.......A....T.....CGA...T...C.....CA....................
2851              -.G...G..........T..A...........C...G..A.A..............A..A...GA...T...C......A.....C.......C......
2626              G.G.....G.....G..TGCC......A.AA.C...G..A.A.C.................G..A...T...G......A.....C......A.......
2821              -.A...G..........T..A......A..A.C...G..A.......................GAC..T...GA.....A.....C..............
Clustal Consensus     *  * *  * ** **   *** **   * ***    * *  ****** * ** * *     *  **  ***   **   **      *  *

                         910       920       930       940       950       960       970       980       990      1000
                  ....|....|....|....|....|....|....|....|....|....|....|....|....|....|....|....|....|....|....|....|
1                 ACACAAGAAAAAGTATACCCATGGGACCAGGCAGAGCATTTTTTACAACAGGAGACATAATAGGAGATATAAGACAAGCACATTGTAACCTTAGTAGAAC
37                .T............G...AT.CA........GG.....C.G.A.G..........T.........A.A.........................A..A...
40                .T.....G..........AT..A........G..........A.G..........A.........A......A...............T......GC.G.
41                .T.......G.........T..A........G..G...A.....CA..............C............GA.....T..............TTTGT
B7                .T.................T..A........G..T.T.....A....---.....A........................................AT..
78                .T.........G......AT..A..G.....A..........A.G..........A.................G.....................G..G.
79                .T.........G......AT..A..G.....G.A........A.G.........GA.................G........................G.
82                .T.........G......AT..A..G.....G.A........A.G..........A.................G...................A.G....
B4                .T..........AG...A.TT.A........G....T.C...A............A...................G........................
B19               .T.............................G.A........A.G...G...........C......C......A.....T.....G.AA...A.G....
K44               .T...............A....A........G..........A............A.................................A...C.GA.GT
Q0                .T.........G......ATC.A........GG.....C...A.G.........CT........................T........A........G.
801               .T................AT..A........G..........A.G..........A.........A..CC...................A...C..A.GG
217               .T.......G......T.AT..A...G....G.....CA...A...........CA......G.................T.....C.A....A..A.G.
181               .T.....G...G.....AATT.A.T..A...G.......GG....ATT...AG.---..G.....A.C.....G...............A...C....G.
2851              .T.....G..........AT..A........G.....C.GG.A.G..........T..................A.....T........A.....GA.G.
2626              .T.....T..........AT...........GG...........G........AGA..................A.....T....C.CTG...A.G....
2821              .T.................T..A........G..........A............A.................................A......A..G
Clustal Consensus    * ***** * *   *      *   ***    *    * *     *     ** ***  *  **    **** ****    *** *

                        1010      1020      1030      1040      1050      1060      1070      1080      1090      1100
                  ....|....|....|....|....|....|....|....|....|....|....|....|....|....|....|....|....|....|....|....|
1                 AGATTGGAATAACACTTTAAAACAGGTAGCTGCAAAATTAAGAAAA------CAATTTGGGAATAATAAAACAATAATCTTTCAGCCATCCTCAGGAGGA
37                .C.A......G........C.....A...T.AT.C...........------G.....A..G.G............GC....AGT......A.......G
40                ...C...C.................A...T.AGT.........G..------.......---GA..............T...A.T.A............G
41                .AG.................G.A........AT..........G..------..G....---GG.............CT...A.T.A.............
B7                .............................T.AT..........G..------.......---.G............G.....A.T.A....G.......G
78                ..CA..................A..A...T.AT........A.G..------.......---GG............G.....A.T.G...T........G
79                ..CA..................A..A...T.AT..........G..------.......---GG............G.T...A.T.A............G
82                ..CA.....G............A..A...T.AT..........G..------.......---GGG.............T...A.T.A............G
B4                .AG......................A...T..A..........G..ATAAAA......A---.G............G.T...A.A.A............G
B19               ...A...C...G...........T.....T..A..........G..------....A.------............G.....A.T.GC...........G
K44               ...A...T....AG........A..A...T.AT..........G..------......A---..............G.....A.T.AC...........G
Q0                ...A......G.........G....A..............CATGC.------G......---.G..C.........GC....A.T.AT...........G
801               .A.C....CAG.GG.....C..TG.A......A..........G..------.......---GG............G.....A.T.A......G.....G
217               ...A......G.A.........A....................G..------.......---.G..................A.T.A............G
181               .AGA......G........G..A..A...T.AG..........G..------......C---CGGG..GC......TC....A.CAA............G
2851              .A.A.....................A..A...A........A.G..------......---.....A.C..T....G.....A.T.A............G
2626              ...A.......CA.......G....A...T..A......C.A....------.........G.G............G.....A........G.......G
2821              CA.A......G........C....AA...T.AA..........G..------......A---.G............G.....ACT.AT...........G
Clustal Consensus       ***      *****  *   **  *   ****     *      * * *       *  * ****  ***     **  * *****

                        1110      1120      1130      1140      1150      1160      1170      1180      1190      1200
                  ....|....|....|....|....|....|....|....|....|....|....|....|....|....|....|....|....|....|....|....|
1                 GACCCAGAGATTGAAAGGTTCAATTTTAATTGTGGAGGGGAATTTTTTTACTGTAATTCAACACAACTGTTTAATAGTACTTGGTATG---CTAATGGAA
37                ........AG......T.CA..G..................G.....C.........A......C...............C...C.GT---T....AAT.
40                ........A....T..T.CA...........................C........C.......C...................A..A---TG.....T.
41                ........A....TG.T.CAT.G........................C.........A........T.......C....G........---.A....AT.
B7                ..T.....A....T.TT.CA..G........................C.........G..........................A..A---T......T.
78                ........A....T..T.CA..G..........A....A........C.........A..........................---------...A.T.
79                ........A....C..T.CA..G........................C.........A.....................G....CC..---AG....AG.
82                ....T...A....T..T.CA..G..........A....A........C.........A..........................---.---AA...A.T.
B4                ........A....T..T.CA..G........................C.................G..................C...---.......T.
B19               ........A....T..T..A..G...C.....C..............C...............A.G................---------------.GC
K44               ........AG...T....CA..G...............A........C......G.................................---.A.CA.AT.
Q0                ..T.....A....T..T.CAT.C........................C.........A.....A................G....T..---GA....A..
801               ........A....T..T.CA...........................C...........C...............TA..G.GTACTA.---T......T.
217               ........A.......T.CA..G........................C....................................A...---A....A.T.
181               ........A....T..CACA..G...............A..G.....C..........................C.....C......A---.......T.
2851              ........AG..AC..T.CA..G......C.................C.........A..T..A...........G.A..C...A..A---G..C.AA..
2626              ........A....T..CACA..G........................C.........A.....A.C.....C.......G..CAACA.AACT....A.T.
2821              ........A....T..T.CA..G........................C..T......A.............................A---T....AAT.
Clustal Consensus    ** * *** **        * *** ** **  **** ** ***** ** ****  *  **   **** **   *
```

```
                         1210      1220      1230      1240      1250      1260      1270      1280      1290      1300
                     ....|....|....|....|....|....|....|....|....|....|....|....|....|....|....|....|....|....|....|....|
1                    CACAAAAA---------AACACGGTAGGA----GATGACCCAATCATACTCCCATGCAGAATAAAACAAATTATAAACATGTGGCAGGGAGTAGGAAAAG
37                   .TTGG..TATTACTAATGGA..A.-....-------...AAAT....C....T.............................................
40                   .TA.TG.---------G.CA.GTA-GCAC--AGA.----AAT.....G....AG....A..................C.........A...........
41                   .TGC.G.G---CTAAAT.T...CA-.A.GGCCAA.....A.T..T.C...........G.............................A..........
B7                   .TTGG..TGGTACGAATGGT..TA-CA..GTCAA.....A.T....C.........................................A.....C....
78                   .T---G..GGGTCAAAA.G...TA-.C..----------AA.T..T.C........................................A..........
79                   .TAG.G..GGGCCAGAT....AT.-GA..----------AAAT....CC.........A.....G.......................A..........
82                   .T---TGGGGGTCAAAT.G...T.-.AC.----------AAAT....C................G.......................A.....G....
B4                   .TTGG..------GAAT---..T.-.A.GGGCA...A..AAT....C.........................G.......A..........
B19                  .TTGG..TGATACTAA-.GGGTCAC.T..CACTA...G.A..C..............A.......................................
K44                  ATACT.---------CG.GT.ATA-.TA---GAAGCAT.AAC....C.A...T...............G..G......C.......A.........G.
Q0                   .TAG.G.G---ACAAAT.GA..AA-.T..----------AAAT....C...........G.....G...........T...................G..
801                  GTTTT..GCCACCACTGG.T.ATA-GTACTCGGA...GGA.C....C.........................................A..........
217                  .TG.G..---------TG.T..TA-GA.GGTCAA....AA......C......G.....G............................A.A..........
181                  .TTGG..G---AGTGAT.------A-.A..GATGA-C..AAAC....C........................................A..........
2851                 G.GC...T----------.......A...G----AT.-------..........A........................T.G......A.A.........
2626                 .TTGG.GTGGTAATAGT..TGACAC....AAA-A....TA......C......................................CA..........
2821                 .TGGG..TGGTGCTAAAGGGT.A.-.TA.CAC---....A.T....A.......................................AG.........
Clustal Consensus                                                * *   **   ****  ****  ***  **  ****   ******    ***** *  *

                         1310      1320      1330      1340      1350      1360      1370      1380      1390      1400
                     ....|....|....|....|....|....|....|....|....|....|....|....|....|....|....|....|....|....|....|....|
1                    CAATGTATGCCCCTCCCAGCAGAGGACTAACTAACTGTACATCAAATATTACAGGACTACTATTAACAAGGGATGGTGGTGTAAAC------------CA
37                   ..................T...C.......T...A....................G..GA....................AAT..T--------------
40                   ..................T..A........T..GA...T................G..G...........A.........A---..--------------
41                   ..................T.CA........T.......T................G..G..G.................CAAT.CT--------------
B7                   ..................TT.......AG.T..GA...T.................T............A..C......AAT..T--------------
78                   ................T.T.GC.....A..T..G....T................G..GA....................AAC..T--------------
79                   ..................T...........T..GA..CT................G..G.....................AAC..T--------------
82                   ................T.T.GC.....A..T..C....TT...............G..GA..........A.........AA.CTG-------------
B4                   .............A....T.GA.....A..T..GA...TT...............GT.............A.........AGT.GT--------------
B19                  ..................T.GA....AA..T..GA..CT.......C..........GT..........A.........TACG.---------------
K44                  ..................T........A..T.......G................G..G......GT...A.........A.TGGT--------------
Q0                   ..................T...T....A..T..GA..CT................G..G...........A.........AAT..T--------------
801                  ..................T.......TAC.T..GA...TT...............G..G...........A.........A---.T--------------
217                  ..................T...T....A..T...G..........C.........G..G...C.......A.........AC....--------------
181                  ..................T........A..T..GA...T................G..G...........A.....G...C.CG.G--------------
2851                 ..................T.GA.....A..T...A..CT................GT.G...........A.........AA..C.--------------
2626                 ..................T...T...AA..T.......TT...............G..G...........A.....G...AGTG.TGGGGGTAGTA---.
2821                 ..................T........A..T..GA...T.C.................GA..........A.....G...AAT...--------------
Clustal Consensus     ************* ** *     ***   * **  **    ***** ********  *  *  **  *** ** ** **

                         1410      1420      1430      1440      1450      1460      1470      1480      1490      1500
                     ....|....|....|....|....|....|....|....|....|....|....|....|....|....|....|....|....|....|....|....|
1                    GACCGA--------------GTATAACGAGAC-CTTCAGACCTGCAGGAGGAAATATGAAGGACAATTGGAGAAGTGAATTATATAAATATAAAGTAGTA
37                   A-AT..GACAAACA-------CGAC.--GAGAC..........CAC......G..............................................
40                   A-A.C.GA--GCCAG-----------.CGAGAC...T.....A.G.......G......G........................................
41                   A-A.A.TAC------------CGAGT.TGAGAT.................G......G...........................................
B7                   A-A.ACGAG-------------.GAC..CGAGAT..........G.......G......G..........................................
78                   A-.A..AAG------------C.AT..AGAGAT..........G.......G..................................................
79                   A-.A.GAAA------------C.AT..AGAGAT..........GG......G......G...........................................
82                   A-A..GGAG-------------.GGGC.TGAGAT..........G..............A..........................................
B4                   .-AA..GAA------------CCAG..CGAGAT..........G..................T.......................................
B19                  ..G.A---------ATGAGAC.GAT---GAGAT..........G.......G......G...........G...............................
K44                  A-AT..TACTGACAAT-----TCC..CGAGAT.........G.G.......G......G...........................................
Q0                   .-TGACCAACAACAC------C.CCC.CGAGAT.........CA......G...........................G.......................
801                  A-ATA.GACGAATGA---------C..CGAGACT..........G.......G......G..........................................
217                  C-AGACTGACAGAAA------CGA...TGAGAC..........A..............G...........G...............................
181                  A-A.A.GACAGGGGACGAGAAC.AG..CGAGAC..........G.......G......GA..T........................................
2851                 -...A.CAA------------C.CC..AGA.TT..........G...............A..T.......................................
2626                 ..A.AGTAGTAAGAATGAGACCGGT..TGAGAT..........G.......G......G...........................................
2821                 A-AT..TACCAACAA------.GAT..CGAGGT..........G.......G......GA..........................................
Clustal Consensus                                   ** *****    *****  ******  **  ********  ********  ******************

                         1510      1520      1530      1540      1550      1560      1570      1580      1590      1600
                     ....|....|....|....|....|....|....|....|....|....|....|....|....|....|....|....|....|....|....|....|
1                    AGAATTGAACCATTAGGAATAGCACCCACCAAGGCAAAGAGAAGAGTGGTGCAGAGAGAAAAAAGAGCAATAGGAGTATTCGGAGCTATGTTCCTTGGGT
37                   .A.G..............G.........T...........................................G.TAC---.C.A..................
40                   .A.......................................................................G.G..---.A.G......T...........
41                   .A................G......................................................GC------..A......T...........
B7                   .A.......................................................................G.G...AC.A.A.................
78                   .A.......................................................................G....---.C.A......T...........
79                   .A................G....................G...............A.................G.G..---.A.A......T...........
82                   .A................G.......T...........A..................................G.G..---.A.A.....GT...........
B4                   .A................G......................................................G.G...A.GC.A.................
B19                  .A............G...G.......T...............G...............G.............T.T..---.C.A......G...........
K44                  .A................C.........T...........................A...............G.G..---.A.A......T...........
Q0                   .A................G..........................................................CAC---.A.G......C...........
801                  .A........CA......G.....................................A...............G.G..---.A.A......G...A..T.....
217                  .A................G....................A.............................................---.A.A................
181                  CA................G.....................................A...............G.G..---.A.A......C...........
2851                 ..................G......................................................A..---......G...........
2626                 ..................G.......................G....C...A...............ATC.G..---.C.A......T...........
2821                 .A................G.........T............................................G.G..---.A.A.....CT...........
Clustal Consensus      * *******  * *** ******* **  *******  **  **** ***  **  **  **********          *****  **  **  ******
```

```
                         1610      1620      1630      1640      1650      1660      1670      1680      1690      1700
                   ....|....|....|....|....|....|....|....|....|....|....|....|....|....|....|....|....|....|....|....|
1                  TCTTGGGAACAGCAGGAAGCACTATGGGCGCAGCGTCACTAACGCTGACGGTACAGGCCAGACAATTATTGTCTGGTATAGTGCAACAGCAGAGCAATTT
37                 ........G...............................G.................................G.........................A......
40                 ........G...............................G.....................T...............................A.AT..C..
41                 ........G............................G..G.....................T.................................A......
B7                 ........G.........................A...T.......................T.................................A....C.
78                 ........G.............................G...........................................................A....C.
79                 ........G.............................T.G.........................................................A....C.
82                 ........G.............................G.GG.........C................C...............................
B4                 ........G.............................A.G.....................T.................................A....C.
B19                ........G.........................A...A...........................................................A......
K44                ........G........................T....A.G..............A.......T.................................A......
Q0                 ........G.............................A.G............................................G.......A....C.
801                ....A...G.............................A.G..........................GC..............................C.
217                ........G.............................A.......................................................AT.....
181                .......GG...............................G..................................................A.A...C..
2851               ....A...G.............................A.......................A...............................A....C.
2626               ........G.............................G.G.......................G...............................A....C.
2821               ........G.............................G.G..........................G.............................A......
Clustal Consensus  **** **  ***********************  **  *  ********* *** ******   **  **************** ***** * *  **  *

                         1710      1720      1730      1740      1750      1760      1770      1780      1790      1800
                   ....|....|....|....|....|....|....|....|....|....|....|....|....|....|....|....|....|....|....|....|
1                  GCTGAGGGCTATTGAGGCGCAACAGCATCTGTTGCAACTCACAGTCTGGGGCATCAAGCAGCTCCAGGCAAGAGTCCTGGCTGTGGAAAGATACCTACAG
37                 ...A..............................................................................................AG.
40                 ......A.......................................................................A.........C.......A..
41                 ......A.....................A.....................................................................AG.
B7                 ..........................................G..............A.......................................AG.
78                 .....................G..............................................................................T
79                 .....................G..............................................................................T
82                 .........................................................A.......................................A..
B4                 .........................................................A.......................................A..
B19                ......................................................T...........................................A..
K44                .........................................................................A.......................AGA
Q0                 .....A...............G......T.......................................................................T
801                .....A............................................................A...............................AGA
217                ......A.......................................................................A.........GC.......A..
181                ......A..................................................A.......GA......A.......................A..
2851               ....................................................................................A............AG.
2626               ......A....................C.............................................C.......................A..
2821               .....A...................................................A..........................A............A..
Clustal Consensus  *** *  ************** *****  ************* *********** ** *******  ****** **** ***** ***  *******   

                         1810      1820      1830      1840      1850      1860      1870      1880      1890      1900
                   ....|....|....|....|....|....|....|....|....|....|....|....|....|....|....|....|....|....|....|....|
1                  GATCAACAGCTCCTGGGGATTTGGGGTTGCTCTGGAAAACTCATCTGCACCACTACTGTGCCTTGGAATACTAGTTGGAGTAA---TAGAACTGTGAAGT
37                 .................A....................................G.............................---..A....C....TC
40                 ............................................T.........G..............G..........C..---....T..C.A..TC
41                 .................A........A.................T..........A.........................---..A.T..C...GTG
B7                 ............................................T........................G...........---..A.T..T....T.
78                 .................A..........................T.........G..............G...........---..A.T..C..G.TA
79                 .................A..........................T..........A.............GT..........---..A..ACC...GTC
82                 ............A........................G................G....A.......................---..A.T..C..G..G
B4                 .....G...............................................................G...........---....T..CA.G.T.
B19                ..................................................................................---..A.T..C..G.AC
K44                ............................................T.....................................---..A.T..C.A..TG
Q0                 ..............A.......T...............G.....T.........G..............G...........---..A.T..TAT..TG
801                ............................................T.........G...........................---..A.T..C..G.TA
217                ............................................T.........G..............G...........---..A.T..C..G.TC
181                ............................................T.........G...........................---..A.T..T..G.TG
2851               ..............A.............................T.........G...............G..........---..A.T..C..G.TA
2626               ..............A.............................T.........................A............AAA..A....TATG...
2821               .................A....................................G..............G...........---..A.T..C....CG
Clustal Consensus  ***** ****** * ** ** *** **********  ***** *********  *** *********  *********  **   ** *          

                         1910      1920      1930      1940      1950      1960      1970      1980      1990      2000
                   ....|....|....|....|....|....|....|....|....|....|....|....|....|....|....|....|....|....|....|....|
1                  ACATTTGGGAGAACATGACCTGGATGCAGTGGGAAAAAGAAATTGAGAATTACACAGGTGCAATATACACCTTAATTGAAGAATCGCAAATCCAGCAGAA
37                 .G........T.....................................C...........CAT........G..................G.A.......G.
40                 .G........T.............G.A.......G...........A...........CTT.............C..........A...T....A...G.
41                 ........A.T.............A.........CG.........AGC..C......AACA.........G.....................A...A..AG.
B7                 .T........T.......................T.G.......A.C.........ACCCTC............C.......TG....G.A......AG.
78                 .A......A.A...............G.......G.G.........C...........CTT........A....C.............G.A...A..AG.
79                 .G........T.......................T.G.......A.C.........AA.A.........A....C.............G.....A..AG.
82                 ........A.T...............G.................AGC...........CTT..........C........A.........G...A..AG.
B4                 ........A.T...............G.......G.G.......A.C...........CTT........A..................G.A...A..AG.
B19                .G........T........A......G.........G.........C.....T.....CTACC......CAA..................G.A...A..AG.
K44                .G........T........A.....AG.......G.G.........C.........A.CTT.............C.....A.T.......A...A..AG.
Q0                 .A......A.T........A..........................C.........A.CTT...................C.........A...A..AG.
801                .G......C.A...............G.......G.G.........C...........CTT........A..................G.A...A..AG.
217                .G.......GT.............A.G.........GC........C..........ACTT........GA.........A.......G.A......AG.
181                ........C.T.......................T.........A.C..G.......AC.AG.......AT.....................A...A..AG.
2851               CA......A.T...................................C...........GTT...........................G.....A..AG.
2626               ........A.T..............AG.........G.........C..C........CTT........AT.........A.......G.A.......G.
2821               .G..C.....T...................................C.........A..TT.............C.....C.........A...A..AG.
Clustal Consensus    ** ***    ********     ****   * *****    ******   ****   ** ** ***      ******    ** *****  *  * **   *** **  *
```

```
                             2010      2020      2030      2040      2050      2060      2070      2080      2090
                    ....|....|....|....|....|....|....|....|....|....|....|....|....|....|....|....|....|....|
1                   AAAGAATGAACAAGAATTATTGAAATTAGATACATGGGCAAGTTTGTGGAATTGGTTTAGCATAACAAATTGGCTGTGGTATATAAGATT
37                  ......................G.........A.................G.......GA.........C................A.A.
40                  ..........A....C.....AG.........A..................C......GA.........G................A.A.
41                  ..........A....C......G.........A........AC...............GA............................A.A.
B7                  ............C........AG....G....AG........................GA.......C..................A.A.
78                  .....................AG.........AG........................GA..........................AGA.
79                  ..........A....C.....AG....G....A.........................GA..........................A.A.
82                  ......................G.........A....T...A................GA.......C.A..............T.A.A.
B4                  ...A.....G............G....G....A.................C.......GA....T....C................A.A.
B19                 ......................GC...G....A.........................GA.........C................A.A.
K44                 .........G............G....G....A....A.....C.........................C...T.A..........A.A.
Q0                  ......................G.........AG........................CAT...T......N.N.............A.A.
801                 .........G............G.........A.........................GA.........A................A.A.
217                 ......................G....G....A.........................GA.........A..................A.
181                 ...............C......GC........A..........C......G.......GA.........G..................A.
2851                ...........T...C...........G...CA.........................GA..........................A.A.
2626                ..........A...........G....G....A.....AC..........G...................................A.A.
2821                ...............G......G....G....A.........................GA..........................A.A.
Clustal Consensus   *** *****    ** *****   *** ***   ***   *   ******  ******   *** ** * * * * ******** *   *
```
