# Supplementary material for: Transmitted/founder (T/F) HIV-1 derived from sexual contact exhibits greater transmission fitness in human cervical tissue than T/F HIV-1 from blood-to-blood contact: Unique glycan profiles on T/F envelopes associated with transmission phenotypes
Source: PLoS Pathog. 2025 May 23;21(5):e1013177. doi: 10.1371/journal.ppat.1013177 (PMC12140434; doi:10.1371/journal.ppat.1013177)
Supplement: S3 Fig — (PDF) [file ppat.1013177.s003.pdf]

```
                           10        20        30        40        50        60        70        80        90       100
TF HIV-1 Env       ....|....|....|....|....|....|....|....|....|....|....|....|....|....|....|....|....|....|....|....|
1                  AEQAWVTVYYGVPVWKEANTTLFCASDAKAYDPEAHNVWATHACVPTDPNPQELPVLNVTENFNMWKNNMVEQMHEDIISLWDQSLKPCVKLTPLCVTLN
37                 TGRL...........................ST.V..................IKLE.........Q.......N.........................
40                 T.KL...I..........T.............T.V..................IELG..........................................K
41                 TGKL..............T...............V..................VKLE....K..A.N.S...............................
B7                 .GNL..............T.............T.V..................VVLG...........................................
78                 .G.L..............T.............T.V..............G...VTLE................I..........................
79                 TGNL..............T.............T.V..............S...V.LE..........................E................
82                 V..L..............T.............T.V..................VNLK.I.......E............N....F.........V..A..
B4                 ..NL..............T.............T.V..................VVLE...........................................
B19                .DNL...........R..T.............T....................VELK.........E.................................
K44                T..L..............T............E..V..................VVLG...........D..............................D
Q0                 ..KM...........................ST.V..............S...VVLG....E.....................................H
801                ..KL..............T.............T.V..................MELA......................E....................
217                ...L..............T.............T.V..................VVLE...........................................
181                -TEL..............T.............T................S...VILE....D.........D............................
2626               TDKL..............V.............T.V..............D...VVLE...........................................
2821               T.NL..............T.............T.V..................VVLE.............................Q...R.........
2851               T.KL..............T............ET.V...................VLE....Y.D....................................
Clustal Consensus      . ***:*******:** ************..*.**************.***: : *:** *: *:*.**:*::****.***: *:***:***:**:*.

                          110       120       130       140       150       160       170       180       190       200
                   ....|....|....|....|....|....|....|....|....|....|....|....|....|....|....|....|....|....|....|....|
1                  CTDVETKRTN----ATDTNATTTRTTSTPSTNSSGVKLEERGEIKNCSFNITTDMRDKVRKEYALFYKLDVIQIDNR-------TDSNDSYRLISCNTSV
37                 ...LRNE-----------SD.NPTISPGKK--------M.A..M......V..SV...R...N....N..IVK...KDEDNNDS.TD.T....T....T.
40                 .N.T.-----------V..T..NT..NSTG-------KM.QEGMT........SI...WQ........I.LVS.N---------KTD.T...........
41                 ...MV.NT.GNS-----------SALNMT------------..V.........EI.N..T.Q.........MP..------------.T..I..N....T
B7                 ...-----------------VNGTSANVT.--------I.K............TI....Q.A..T..S...VP...D------QDN.SS....TN.....
78                 ..ELSN----------ANYT.NSSREEMNN---------G.E.M.........SL..R.T.......S...VP..E--------GSN.T....T......
79                 ..ELS--------------T.NSS----------------...M.....K.A.KL.NRM........S..IVP..ED----KKNNTN.T...........
82                 ...LNV----------.L.VNN.INS.LG-----------EM.M.......SIKT.S.AQ..........IVP..E--------NNN.T...........
B4                 ..N.NV----------TNLKNE.N.N.SSG-----GEKM.E..M......V..LI.N.RKT..........MP..----------HD.T..T..N..S.T
B19                ...LGN-------------V.N.TNSNGEM--------M.K..V.....K....IK.RT............VP.N------------DTR...V......
K44                ...LN.TISKNATN--SR.T.N.TSS.WET--------M.K............RR...MK....F..NF.IVKVKDDD---TNT.NTST....T......
Q0                 ..N.T.------------...N.TSD.-------------WEDM.........SI....T...........VP.GK----------DKN..T..N.....
801                ...LKN----------T..SN..N...SRGETMEKGE.M.K............SI.S.MQ...........VP..D--------DN..T...........
217                ...LGN----------A.TTNNATNINSS.I.G--GLKM.K..M.........SR.GRMQ...........VS...----------D.T...........
181                ...YGNDTNTNSGND.S.KGNN.TSGN.T.S---NWWQM.K..M.........NI..RRQ....F......VP..ED-------NTN.T...........
2626               ...YNN------------T..N.TSSA.TTAS.ANKTAK.EAVM.........NV....KR......N...VKLEE----------DET....V......
2821               ...Y------------VK.V.NATS.NAT.--------S.G..M......V..N....KQ.......N..IV....--------DNA.N...........
2851               ...WTNG--------..W.T.NSNN.TISKE-----ETI.G..M.........ATG..-K..R.F......AP...----------..T...........
Clustal Consensus  *.:                                    :.****:::    .:     * **.:*:  :            .  * *  .**::.

                          210       220       230       240       250       260       270       280       290       300
                   ....|....|....|....|....|....|....|....|....|....|....|....|....|....|....|....|....|....|....|....|
1                  LTQACPKVSFEPIPIHYCAPAGFAILKCNNESFNGTGPCTNVSTVQCTHGIKPVVSTQLLLNGSLSKE-DVIIRSKNFSENTDIIIVQLNTSVTISCIRP
37                 V............................DKKY....A.K...........R.............AE.-E.V...A...D.DQN.....KE..E.T.T..
40                 I.....................YG....KDKK.......K...........R.............AE.-E.VL..A...N.AKT...H..K..E.N.T.I
41                 I...........................RDKG.....S.K.........................AE.-..VV..A..TN.AKT.....KDP.V.N.T..
B7                 I........................L...DKK...................R.............AE.-E.V...E..TD.AKT......N..V.N.T..
78                 I...............L............DKK.......K...........R.............AE.-E.V...E..TN.AKS......E..V.N.T..
79                 I...........................KDKK...................R.............AE.-E.V...E..TN.AKT......E..V.N.T..
82                 I...............F............DKK.......K...........R.............AE.-E.V...E..TD.VKT......E..E.K.T..
B4                 I............................DKK...K...K...........R.............AE.-.IV...E..TD.AKN......V.IE.N.T..
B19                I............................DKQ.I.................R.............AE.-E.V...V...D.AKT......K..E.T.T..
K44                I............................DKK...................R.............AEN-ETV...A..TD.AKT......EP.N.T.M..
Q0                 I............................DKK...S.Q.............R.............AEK-E.....A...D.GKT...H..K..E.N....
801                I............................DKK.......K...........R.............AEK-E.V...E..TD.AKT......K..E.E.V..
217                I............................DKK.....I.N...........R.............AE.-E.....V...D.AKT......E..E.N.T..
181                I............................DKK.....S...........................AE.-EIV...E.LTN.AKT..IH..E..P.H....
2626               V......IT.....................KT.................................AEGGE.M...A..TN.AKT.....SK..A.N.T..
2821               I.........................R..DKK...................R.............AE.-E.V......TN.AN.......D..E.N.T..
2851               I......I.....................DKK.....S.............R.............AE.-E.V.......D.AK.......E..P.N.T..
Clustal Consensus   :******::******* *****:.:*:*.:: : *.* *.***********:*************:: : ::** *:::* . **::*. .: * * *

                          310       320       330       340       350       360       370       380       390       400
                   ....|....|....|....|....|....|....|....|....|....|....|....|....|....|....|....|....|....|....|....|
1                  NNNTRKSIPMGPGRAFFTTGDIIGDIRQAHCNLSRTDWNNTLKQVAAKLR-KQFGNNKTIIFQPSSGGDPEIERFNFNCGGEFFYCNSTQLFN-------
37                 .......VHT...G.LYA......K........NK.Q..D..Q.IVIQ..-.E.RE....A.S..T.....V.MHS...........T.P...-------
40                 ........HI......YA..E...N.K.......AA..H.....IVS...--EQFG......NQ........VMH..............P...-------
41                 F....R...I.....I.Q....T....K.Y....FVS.....RK..I...--EQFG....T.NQ........VMHS...........T.....-------
B7                 .........I...SV.Y.-.E.............N..........VI...--EQFE....V.NQ.A......VLHS...........A.....-------
78                 ......G.HI......YA..E.............GAA......KIVI..K--EQFG....V.NR........VMHS...R.......T.....-------
79                 ......G.HI...K..YA..G..............AA......KIVI...--EQFG....V.NQ........AMHS...........T.....-------
82                 ......G.HI...K..YA..E............NG.A.K....KIVI...--EQFGD.....NQ.....L..VMHS...R.......T.....-------
B4                 ......K.TL....VLY...E......R........S.......IVE...EIKQFK....V.KQ........VMHS.................-------
B19                .............K..YAR...T....K.Y.EING.E.HS...L.VE...---EQY....V.NR........VMYS.............K...-------
K44                ........TI......Y...E...........ITEVE.YKA..KIVI...--EQF.....V.NH.......VV.HS..........D......-------
Q0                 ......G.HL...G.LYA..A........Y..I..AE..D..R.I....H-AE.-E....A.NH........VMHT...........T.K...-------
801                .S......HI......YA..E...NP......ITKGN.TEA.QWI.E...--EQFG....V.NQ........VMH..................-------
217                .....R..HI.A...IY...A........Y.Q.NKAE..E...K......--EQFE......NQ.........MHS.................-------
181                ......G.NLVQ...W.NS-EVV.N.......IT.AR..D..EKIVR...--EQFPGS..S.NK........VTHS.................-------
2626               ....S...H....G...A..R......K.Y.TVNG.E..T..R.IVE.FK-....E....V.K..A......VTHS...........T.N...SSSTELN
2821               .........I......Y...E...........I.KSK..D..Q.IVK...--EQFK....V.TH........VMHS...........T.....-------
2851               H.......HI.....WYA.........K.Y..I.EAK.......ITE..K--EQF.KTI.V.NQ.......VTMHS...........TSK...-------
Clustal Consensus    .** : :   * . :   : *. ::*:* :.  * :*. :. :::     . * *  *:*** *:  ..*** ******::: ***
```

```
                          410       420       430       440       450       460       470       480       490       500
                  ....|....|....|....|....|....|....|....|....|....|....|....|....|....|....|....|....|....|....|....|
1                 STWYANGTQKN----TVGDDPIILPCRIKQIINMWQGVGKAMYAPPSRGLTNCTSNITGLLLTRDGG-----VNQTEY---NETFRPAGGNMKDNWRSEL
37                ...QF.N.WNIT--NGTE.EN.T.S.....................IS..IK........I......------.NN.TNT-T.....H..D.........
40                ...NM...NET---SSTEN--.M.Q.K......L..E.........IK..IR.S.............------.NQS---QT.....G..D.R.......
41                .S....D.AELNI-TKGPN.T.T.............E.........IQ..I..S.............NT----.N.----ES.I......D.R.......
B7                ...NI...WNGTNGT.ESN.T.T.............E.........I..QIR.S.............NN----.TS----DT.I...G..D.R.......
78                ...N---STEGS---KSTNET.T.............E.........IA.QIS.S......I......------.N..S--NT.I...G..D.........
79                .S.PE.E.REGP---DNNGEN.T...K.R.......E.........I...IR.S.............------.N.GN--NT.I...G..D.R.......
82                ...EN--STWGS---NSTEQN.T.....R.......E.........IA.QIT.L......I......------KLNGS--GP.I...G............
B4                ...H....W..---TEGA.NN.T..........R..E.........IE.QIR.L.............------SSE.N--QT.I...G............
B19               ...PW.D.KG----SHDTNGTL....K...................IE.KIR.S.............------YESNE--TD.I...G..D.R.......
K44               .....TDNTTSNNRS----IN.TIL.....VV.T..E.........I..QI..A.........V...IG----.D.DN--ST.I...G..D.R.......
Q0                ...FG.E.R--E--TNRTNEN.T................R......IS.QIR.S.............------.NVTNNTTP.I...Q............
801               YSVLV..SF.PPLDNSTRNGT.T.............E.........I..YIR.L.............------.NKTN--DT.....G..D.R.......
217               ...ND.S.ENDT---RGSNET.T.............E.........IS.QIK...............------TNQTDRNET.....E....R.......
181               ....T...W.S---DKRD.EN.T.............E.........I..QIR.S.............LE----.K.GDENKT.....G..D.R.......
2626              ...SG.SN------D.GKN.T.T.............Q.........IS.KI..L.............SDGGSK.SSKNETGT.I...G..D.R.......
2821              ....I.N.GNGAKGS-DNT.T.K.............E.........I..QIR.S......I......NN----.D.NN--DT.V...G..D.R.......
2851              G..NSTKRAN------NTEGI...Q........R..E.........IE.QIK.S.............------KT.NN--TT.F...G............
Clustal Consensus    :                : : *:*:*::* ** **:****** *   * ******:*.****            * *** **:*:*******

                          510       520       530       540       550       560       570       580       590       600
                  ....|....|....|....|....|....|....|....|....|....|....|....|....|....|....|....|....|....|....|
1                 YKYKVVRIEPLGIAPTKAKRRVVQREKRAIGVFGAMFLGFLGTAGSTMGAASLTLTVQARQLLSGIVQQQSNLLRAIEAQQHLLQLTVWGIKQLQARVLA
37                ......KV....V................VT-L.........A...........................N.............................
40                ......K......................V.-M..L......A.................L.........N.............................
41                ......K.....V................A--L..L......A.................L.........N...........M.................
B7                ......K......................V.TI.........A.................L.........N.............................
78                ......K......................V.-L..L......A.........V.................N.............................
79                ......K.....V.....R..........V.-I..L......A...........................N.............................
82                ......K.....V................V.-I..L......A.........VA..A...........................................
B4                ......K.....V................V.ML.........A.........M.......L.........N.............................
B19               ......K.....V................F.-L..V......A.........I.................N.............................
K44               ......K.....L................V.-I..L......A.......V.M.......L.........N..........................I..
Q0                ......K.....V.................T-M..L......A.........M.................N...K.........................
801               ......K...I.V................V.-I...IF....A.........M.....................K.........................
217               ......K.....V..................-I.........A.........I.................N.............................
181               ......Q.....V................V.-I..L......A...........................N.......................R..I..
2626              ............V........A......DL.-L..L......A.........V.................N..........................L..
2821              ......K.....V................V.-I..L......A.........V.................N...K.........................
2851              ............V..................-I..V......A.........I.......K.........N.............................
Clustal Consensus  ******::**:*:*****:**.******    :**:::****:*******.*::**.***  *********.***:*******:***********:**:**

                          610       620       630       640       650       660       670       680       690       700
                  ....|....|....|....|....|....|....|....|....|....|....|....|....|....|....|....|....|....|....|....|
1                 VERYLQDQQLLGIWGCSGKLICTTTVPWNTSWS-NRTVKYIWENMTWMQWEKEIENYTGAIYTLIEESQIQQKKNEQELLKLDTWASLWNWFSITNWLWY
37                .....R..................A........-.K.LNQ..D...........D....I..S......N..E.......E..K.....S..D.......
40                .....K..................A....A...-..SLNQ..D.....E..R.......L....L....F..E...KD..E..K........D..K....
41                .....R..................N........-.KSLSD..N....K..DE..S...NT..S......N..E...KD..E..K..N.....D.......
B7                .....R.......................A...-.KSLN...D.......DR..N...TL....L.DA.N..E...H...E..K........D..H....
78                .....H..................A....A...-.KSLDK..K.....E..R..D....L..N.L....N..E.......E..K........D.......
79                .....H..................N....V...-.KNLSQ..D.......DR..N...NT..N.L.......E...KD..E..K........D.......
82                .....K....M.......R.....A........-.KSLED..N.....E.....S....L......K..S..E.......E..K.SN.....D..Q....
B4                .....K.......................A...-..SQD...N.....E..R..N....L..N......N..E.......E..K.....T..D.S.....
B19               .....K...........................-.KSLEQ..D.....E..R..D....Y..Q......N..E.......A..K........D.......
K44               .....R...........................-.KSLNE..D....IE..R..D...SL....L.N..N..E.......E..K.T..............
Q0                .....H.......L..........A....A...-.KSYNE..N...........D...SL......Q..N..E.......E..K........H.S.XX..
801               .....R..................A........-.KSLDK..Q.....E..R..D....L..N......N..E.......E..K........D..K....
217               ..A..K..................A....A...-.KSLDQ..G....KE..S..D...DL..R...K..N..E.......E..K........D..K....
181               .....K..................A........-.KSLDD..H.......D...NK..DE..N......N..E....D..A..K.....S..D..K....
2626              .....K.......................N...K.K.YE...N....IE..R..D....L..N...K..N..E...K...E..K.D...S..........
2821              .....K..................A....A...-.KSLNE..D...........D...SL....L.Q..N..E.......E..K........D.......
2851              .....R..................A....S...-.KSLDT..N...........D....L............E...LD.....Q........D.......
Clustal Consensus  ** **:****:** ****:***** **** *** *:. . ** **** :*: **.:** ** *:*.:* **:*** :** ** * .**.** *::  **

                  ..
1                 IR
37                .K
40                .K
41                .K
B7                .K
78                .K
79                .K
82                .K
B4                .K
B19               .K
K44               .K
Q0                .K
801               .K
217               ..
181               ..
2626              .K
2821              .K
2851              .K
Clustal Consensus *:
```
