# Supplementary material for: Transmitted/founder (T/F) HIV-1 derived from sexual contact exhibits greater transmission fitness in human cervical tissue than T/F HIV-1 from blood-to-blood contact: Unique glycan profiles on T/F envelopes associated with transmission phenotypes
Source: PLoS Pathog. 2025 May 23;21(5):e1013177. doi: 10.1371/journal.ppat.1013177 (PMC12140434; doi:10.1371/journal.ppat.1013177)
Supplement: S1 Table — (PDF) [file ppat.1013177.s024.pdf]

S24 Table. Lectin Microarray Information

	Description*
**1. Sample: Glycan-containing sample (e.g. glycan, glycoprotein, cell lysate, cell, glycopeptide, etc.)**	
Description of Sample	HIV-1 Env lysates.
Sample preparation protocol	HIV-1 Env lysate samples were ready for labeling prior to analysis on the lectin microarrays, and thus required no sample preparation.
Labeling protocol for sample detection	Samples are labelled with Alexa Fluor 555-NHS (Thermo Fisher).
Two-color reference (if used)	A pooled reference of all samples labeled with Alexa Fluor 647-NHS (Thermo Fisher).
Assay protocol	Lectin microarrays are blocked with blocking buffer (50 mM ethanolamine and 100 mM boric acid) for one hour at room temperature. Slides are rinsed once with PBST (0.01%) and once with PBS for 5 minutes each, then dried using a slide spinner. Each slide is mounted on a 24-well format hybridization cassette (Arrayit), in which each well contains a subarray. To each well, 5 µg of sample and pooled reference are added, then diluted with PBST to reach the final volume (100uL) and concentration (0.005%). Slides are incubated on an orbital shaker for one hour at room temperature in the dark. After hybridization, arrays are washed with PBST (0.01%) once for one minute, then once for five minutes. Arrays are lastly washed once with PBS for 1 minute, then once for ten minutes. Once finished, slides are removed from the cassette and briefly immersed in ultrapure water, then dried using a slide spinner.
**2. Lectin Library**	
General description of the lectin library used in the array	Lectin microarrays are generated in house.
List of lectins and/or glycan-binding proteins, their source, concentration, and buffer	Please see **S21 Table**.
Modification of lectins	N/A
**3. Immobilization Surface; e.g., Microarray Slide**	

Immobilization surface	Nexterion Slide H Barcoded 3D Hydrogel Coated.
Manufacturer	Schott North America.
Custom preparation of the surface	N/A
**4. Array Production**	
Description of Arrayer	Nano-Plotter 2.1 piezoelectric printer (GeSim, Germany) with cooled microwell plate holder and cooled printing deck.
Lectin deposition	Triplicates of each lectin are printed onto each subarray.
Printing conditions	Dilute lectins to the pre-determined concentrations in the print buffer (final concentration of print buffer: 1 mM monosaccharide in PBS, 5 ng/mL Atto 532; Please see Supplemental Table 1 for the concentrations of lectins). Load the mixed solution to the microplate. Before printing, check the humidity of the print chamber. The humidity should be kept around 50% throughout the entire print. Ensure both microwell plate holder and printing deck are cooled. Adjust the cooling temperature based on ambient temperature and the temperature of the cooled slide deck surface, preventing moisture building up inside the print chamber. Once printing is complete, allow the slides to dry for at least one hour.
Array layout	Each microarray contains 21 subarrays (3 columns and 7 rows). In each subarray, triplicates of a lectin are printed, with six lectins in each row. The number of columns is 18, and the row number depends on how many lectin probes are printed on the arrays (i.e. 132 lectins require 22 rows).
Quality control	The well-characterized glycoproteins glycophorin, ovalbumin, asialofetuin, 1:1 A549:HEK293T cell lysate, human serum, fetuin, and RNase B are used for quality assurance of the printed microarrays.
**5. Detector and Data Processing**	
Instrument (scanner, flow cytometer)	Fluorescent Slide Scanner GenePix 4400A (Molecular Devices).
Instrument settings	A low resolution preview scan of the slide is performed to adjust photomultiplier tube (PMT) gain for each channel (Alexa Fluor 555: 532nm, Alexa Fluor 647: 635nm) so that the signals are not saturated and within the linear detection range. After adjusting PMT gain, slides are scanned in each channel at 5 µm resolution.
Image analysis software	GenePix Pro 7 (Molecular Devices).
Data processing and statistical analysis	Extracted data is processed for quality checks using Grubbs outlier test with $\alpha = 0.05$. Log2 values of the average signals are median-normalized over the individual subarray in each channel.

6. Lectin Microarray Data Presentation	
Data presentation and interpretation	Hierarchical clustering of the processed data is performed using Pearson Correlation coefficient, and volcano plots generated using R (v3.6.1) and RStudio (Build 576).
**7. Data Location**	
Data Location	https://doi.org/10.7303/syn64297909
