# Supplementary material for: Transmitted/founder (T/F) HIV-1 derived from sexual contact exhibits greater transmission fitness in human cervical tissue than T/F HIV-1 from blood-to-blood contact: Unique glycan profiles on T/F envelopes associated with transmission phenotypes
Source: PLoS Pathog. 2025 May 23;21(5):e1013177. doi: 10.1371/journal.ppat.1013177 (PMC12140434; doi:10.1371/journal.ppat.1013177)
Supplement: S2 Table — (PDF) [file ppat.1013177.s025.pdf]

S25 Table. Lectins used in microarrays

**Lectin**	**Species/Origin**	**Print Conc. (μg/mL)**	**Rough Specificity /Inhibitory monosaccharide**	**Vendor/Source**
AAL	*Aleuria aurantia*	2000	Fucose	Medicago/Vector
ACA	*Amaranthus Caudatus*	2000	Gal-β1,3-GalNAc	EY
AIA	*Artocarpus integrifolia*	2000	β1,3-GalNAc	EY/Glycomatrix/Vector
AMA	*Allium moly*	2000	Oligo mannose	Vector
Anti-B.G. Lewis A	MAb mouse IgG1 [7LE]	undiluted	Lewis A	Abcam/Sigma
Anti-B.G. Lewis B	MAb mouse IgM [2-25LE]	undiluted	Lewis B	Abcam/Sigma
Anti-B.G. Lewis X	MAb mouse IgM [P12]	undiluted	Lewis X	Sigma
Anti-B.G. Lewis Y	MAb mouse IgM [F3]	undiluted	Lewis Y	Abcam
Anti-Blood Group B	MAb mouse IgM [HEB-29]	undiluted	Blood Group B	Abcam/ Thermo Fisher
Anti-Blood Group H	MAb mouse IgG3 [17-206]	undiluted	Blood Group H	Thermo Fisher
Anti-B.G. Sialyl Lewis A	MAb mouse IgG1 [GT933]	undiluted	Sialyl Lewis A	GeneTex
Anti-IgA	MAb IgG1 [KT41]	undiluted	IgA	Abcam
Anti-IgG	MAb IgG2a [KT131]	undiluted	IgG	Abcam
Anti-IgM	MAb IgG1 [KT16]	undiluted	IgM	Abcam
Anti-MBL	MAb IgG1 [3B6]	undiluted	Mannan Binding Lectin	Abcam
AOL	*Aspergillus oryzae*	2000	Fucose	TCI America
ASA	*Allium sativum*	2000	Mannose	EY
BanLec H84T	*Musa acuminate*	2000	High Mannose	Gift from Dr. David Markovitz
BC2L-A	*Burkholderia cenocepacia*	2000	Mannose	Elicityl
BPA/BPL	*Bauhinia purpurea*	2000	β-Gal / β-GalNAc	Vector
CA	*Colchicum autumnale*	2000	Bi-antennary N-linked glycans	EY
ConA	*Canavalia ensiformis*	2000	Tri-mannose core	Thermo Fisher/Vector
DBA	*Dolichos biflorus*	2000	GalNAc	Vector
diCBM40	engineered NanI from *Clostridium perfringe ns*	1500	α Sialylation	Generated in house

DSA	*Datura stramonium*	2000	LacNAc	Vector
ECA	*Erythrina cristagalli*	2000	LacNAc	EY/Vector
GNA/GNL	*Galanthus nivalis*	2000	Oligo mannose	Sigma/Vector
GS/GSL-I	*Griffonia simplicifoia-I*	2000	α-Gal / Lac	Vector
GS/GSL-II	*Griffonia simplicifoia-II*	2000	GlcNAc	Vector
HHL	*Hippeastrum hybrid*	2000	Oligo/High mannose	BioWorld
HPA	*Helix pomatia*	2000	Blood Group A	Sigma
LcH	*Lens culinaris*	2000	Core Fucose	Aniara Diagnostica/EY/Medicago
LEA/LEL	*Lycopersicon esculentum*	2000	GlcNAc	Vector
LTL Lotus	*Lotus tetragonolobus*	2000	Fucose	EY/Vector
MAA/MAL-I	*Maackia amurensis-I*	2000	Sialylation/Sulfation	EY/Vector
MAA/MAL-II	*Maackia amurensis-II*	2000	Sialylation/Sulfation	Vector
MNA-G	*Morus nigra Morniga G*	2000	GalNAc	EY
MNA-M	*Morus nigra Morniga M*	2000	Oligo mannose / Gal	EY
MPA/MPL	*Maclura pomifera*	2000	β1,3-GalNAc	Vector
NPA/NPL	*Narcissus pseudonarcissus*	2000	Oligo mannose	EY
PA-IIL	*Pseudomonas aeruginosa*	2000	Fucose	Elicityl
PHA-E	*Phaseolus vulgaris Erythroagglutinin*	2000	Bisecting GlcNAc	Sigma/Vector
PHA-L	*Phaseolus vulgaris Leukoagglutinin*	2000	β1,6 Branching N-Link glycans	Medicago/Vector
PhoSL	*Pholiota squarrosa*	2000	Core Fucose	Bio Basic
PNA	*Arachis hyogaea*	2000	Gal-β1,3-GalNAc	EY/Vector
Recombinant Protein A	*Staphylococcus aureus*	2000	Immunoglobulins	Thermo Fisher
Recombinant Protein G	*Streptococcus* Group G	2000	Immunoglobulins	Thermo Fisher
Recombinant Protein L	*Peptostreptococcus magnus*	2000	Immunoglobulins	Thermo Fisher
PSA	*Pisum sativum*	2000	Core Fucose	Vector
PSL	*Polyporus squamosus*	2000	α2,6 sialylation	TCI America
PTA/PTL-I	*Psophocarpus tetragonolobus-I*	2000	α2 Fucose	BioWorld

PTA/PTL-II	*Psophocarpus tetragonolobus-II*	2000	α2 Fucose	BioWorld/Glycomatrix
RCA120	*Ricinus Communis Agglutinin I*	2000	Gal / Lac	Vector
rGRFT	*recombinant Griffithsin*	1700	High mannose	Generated in house
Ricin B Chain	*Ricinus communis*	2000	Gal	Vector
RCA120	*Ricinus communis*	2000	Gal	Vector
RPA	*Robinia pseudoacacia*	2000	Multiantennary N-glycans with bisecting GlcNAc	EY
SBA	*Glycine max*	2000	LacdiNAc	Vector
SLBR-B	*Streptococcus gordonii M99*	2300	α2,6 sialylation	Generated in house
SLBR-H	*Streptococcus gordonii DL1*	2000	α2,3 sialylation	Generated in house
SLBR-N	*Streptococcus gordonii UB10712*	2000	α2,3 sialylation	Generated in house
SNA	*Sambucus nigra*	2000	α2,6 sialylation	EY/Vector
SNA-II	*Sambucus nigra-II*	2000	α2 Fucose /oligo mannose	EY
STL	*Solanum tuberosum Agglutinin*	2000	LacNAc	Vector
TJA-II	*Trichosanthes japonica-II*	2000	α2 Fucose	Aniara Diagnostica/Medicago
TL	*Tulipa sp.*	2000	GlcNAc	EY
UDA	*Urtica dioica*	2000	GlcNAc / Oligo mannose	EY
UEA-I	*Ulex europaaeus-I*	2000	α2 Fucose	BioWorld/GeneTex/Vector
UEA-II	*Ulex europaaeus-II*	2000	GlcNAc	EY/Vector
VVA	*Vicia villosa*	2000	Terminal GalNAc	Vector
WFA	*Wisteria floribunda*	2000	GalNAc-β1,4	Vector
WGA	*Triticum vulgare*	2000	GlcNAc	GeneTex
